# Comparative proteomic analysis of eggplant (*Solanum melongena* L.) heterostylous pistil development

**DOI:** 10.1371/journal.pone.0179018

**Published:** 2017-06-06

**Authors:** Yikui Wang, Ake Liu, Wenjia Li, Yaqing Jiang, Shiwei Song, Yan Li, Riyuan Chen

**Affiliations:** 1 College of Horticulture, South China Agricultural University, Guangzhou, China; 2 Institute of Vegetable Research, Guangxi Academy of Agricultural Sciences, Nanning, China; 3 State Key Laboratory of Genetic Engineering, School of Life Sciences, Fudan University, Shanghai, China; University of Naples Federico II, ITALY

## Abstract

Heterostyly is a common floral polymorphism, but the proteomic basis of this trait is still largely unexplored. In this study, self- and cross-pollination of L-morph and S-morph flowers and comparison of embryo sac development in eggplant (*Solanum melongena* L.) suggested that lower fruit set from S-morph flowers results from stigma-pollen incompatibility. To explore the molecular mechanism underlying heterostyly development, we conducted isobaric tags for relative and absolute quantification (iTRAQ) proteomic analysis of eggplant pistils for L- and S-morph flowers. A total of 5,259 distinct proteins were identified during heterostyly development. Compared S-morph flowers with L-morph, we discovered 57 and 184 differentially expressed proteins (DEPs) during flower development and maturity, respectively. Quantitative real time polymerase chain reactions were used for nine genes to verify DEPs from the iTRAQ approach. During flower development, DEPs were mainly involved in morphogenesis, biosynthetic processes, and metabolic pathways. At flower maturity, DEPs primarily participated in biosynthetic processes, metabolic pathways, and the formation of ribosomes and proteasomes. Additionally, some proteins associated with senescence and programmed cell death were found to be upregulated in S-morph pistils, which may lead to the lower fruit set in S-morph flowers. Although the exact roles of these related proteins are not yet known, this was the first attempt to use an iTRAQ approach to analyze proteomes of heterostylous eggplant flowers, and these results will provide insights into biochemical events taking place during the development of heterostyly.

## Introduction

In flowering plants, different strategies have evolved to avoid selfing and promote outcrossing, of which heterostyly is one of the most effective mechanisms. Heterostyly, a complex floral polymorphism, can aid in environmental adaptations of plants and accelerate species diversification [1,2]. Heterostyly has arisen independently in at least 20 lineages and is present in 199 genera, distributed among 28 families in 15 orders [1,3]. Heterostylous plants usually include two (distyly) or three (tristyly) genetic morphs with reciprocal displacement of sexual organs (stigmas and anthers) within an individual [4]. For example, in eggplant (*Solanum melongena* L.), plants produce two types of flowers (distyly): either long-styled flowers with anthers attached midway along the floral tube (L-morph or pin), or short-styled flowers with anthers attached at the top of the floral tube (S-morph or thrum). This character promotes outcrossing between morphs via delivery and uptake of pollen by pollinators [5].

Although many angiosperms are heterostylous, only a few differentially expressed genes (DEGs) have been detected for the condition, and the regulatory molecular mechanisms are not well understood. Ushijima *et al*. [6] elaborated molecular differences by comparing transcripts and proteins in the thrum and pin flowers of *Linum grandiflorum* Desf. These floral phenotypes were known to be regulated by the S locus and differed in style length, pollen size, and anther length [7]. Four genes, *TSS1*, *AP1*, *MYB21*, and *SKS1*, were predicted to be related to heterostyly development [6]. However, there was no difference in messenger ribonucleic acid accumulations of these four genes, indicating that they were controlled by floral morph-specific post-transcriptional regulation [6]. Transcriptome analysis for both *Primula veris* L. and the closely related species *P*. *vulgaris* Huds. demonstrated that 113 candidate heterostyly genes showed significant floral morph-specific differential expression [8].

Since the development of RNA sequencing (RNA-seq) technology, it has been easy to document changes in gene expression at the transcriptional level. However, the transcriptional changes are not always directly related to expression of the corresponding proteins because of posttranslational regulatory mechanisms [9,10], alternative splicing, and protein degradation [11]. Proteomic approaches provide valuable tools for monitoring developmental profiles directly at the protein level and therefore have been widely used [12–14]; approaches include two dimensional gel electrophoresis (2-DE), differential in gel electrophoresis (DIGE), tag-based labeling of proteins (isotope-coded affinity tags (ICAT)), stable isotope labeling with amino acids in cell culture (SILAC), isobaric tags for relative and absolute quantification (iTRAQ), protein-protein interaction, and protein modifications [15]. iTRAQ is a powerful technology that allows identification of numerous proteins between different samples [13]. Furthermore, if iTRAQ returns a sufficient number of differentially expressed proteins (DEPs), pathway and protein-protein interaction analyses can be conducted [13,14,16].

iTRAQ provides advantages in labeling of complex samples, with comparatively high throughput and identification of low-abundance proteins in complex samples [17,18]. Since Ross *et al*. [19] first published an approach using iTRAQ to examine the global protein expression of a wild-type yeast strain, this technique has been widely used to document quantum changes in DEPs in plants and animals [12–14]. Meng *et al*. [20] then applied iTRAQ in a proteomic study of blood cells infected with *Spiroplasma eriocheiris*. Yang *et al*. [14] discovered genes related to grain development in an analysis of wheat grain protein expression at different stages. The study of regulated protein expression levels in different eggplant flower morphs should provide insights into heterostyly developmental mechanisms.

Eggplant is a heterostylous plant that is widely cultivated [21]. The S-morph flowers generally possess a small and highly reduced gynoecium, and are often functionally staminate, limiting production. Therefore, understanding the molecular genetics that regulate heterostyly could lead to improved selection for better production. In this study, we performed an iTRAQ-based quantitative proteome analysis of pistils of two flower morphs from the budding to the blooming stage. The general workflow is shown in S1 Fig. Our results provide information about differences in proteins during heterostylous development and highlight the value of proteomics in characterizing complex biochemical processes.

## Materials and methods

### Floral measurements

Eggplant B3-3 lines were grown at the Guangxi Academy of Agricultural Sciences and cultivated in a field using conventional methods. Plants produced two morphologically different types of flowers: long morph (L-morph) and short morph (S-morph). We randomly selected 30 S-morph and 40 L-morph flowers from budding to blooming to measure the pistil length and bud length with a Vernier caliper and conducted a correlation analysis using Microsoft Excel 2016.

### Stigma-pollen interactions

The flowers were emasculated in bud and bagged to exclude pollinators before and after hand pollination. The stigmas were collected at 1, 2, 4, 6, 8, 12, 24, or 48 h after pollination and fixed for more than 24 h in a mixture of Formalin-acetic acid-ethanol. The samples were washed with distilled water and immersed in 2 mol·L^−1^ NaOH in a 60°C water bath for 12 h. Specimens were washed again with distilled water and were stained with 0.1% aniline blue for 4 h, and then mounted on glass slides. The samples were covered with 80% glycerin and observed under an inverted fluorescence microscope (Olympus BX51, Japan).

### Female gametophyte development

To investigate whether differences in embryo sac development reduced successful fertilization, we sectioned flowers embedded in paraffin. From budding to blooming, we collected S-morph and L-morph pistils every 2 days and fixed them in a Formalin-acetic acid-ethanol mixture. The samples were dehydrated in an ethanol series (10 min each in 35%, 55%, 75%, 85%, 95%, and 100% [v/v]), cleared in a xylene series (10 min each in 35%, 55%, 75%, 85%, 95%, and 100% [v/v]), and embedded in paraffin (melting point: 54–56°C) for 48 h at 56°C. Embedded specimens were serially sectioned at a thickness of 6 μm and mounted on glass slides. They were prepared with a 2% ferrovanadium mordant for 30 min, stained with 5% hematoxylin for 1.5 h, and then destained with saturated picric acid for 1.5 h. Finally, coverslips were mounted with neutral balsam and the slides were observed under an OLYMPUS BX-51 light microscope (Olympus Co. Ltd., Japan).

### Protein extraction

We divided flower development into five stages from budding to blooming: 0, 3, 6, 10, or 13 days after budding (DAB). The flowers at 0, 3, and 6 DAB were considered development samples. We collected mixed samples of pistils at 0, 3, and 6 DAB from S-morph and L-morph flowers. Pistils collected at 13 DAB were kept separately as mature flower samples. All tissue samples were stored at −80°C liquid nitrogen until protein extraction.

The pistil samples were ground into powder under liquid nitrogen and extracted with lysis buffer A (7 M urea, 2 M thiourea, 4% 3-[(3-cholamidopropyl)dimethylammonio]-1-propanesulfonate, 40 mM Tris-HCl, pH 8.5) containing 1 mM phenylmethylsulfonyl fluoride and 2 mM ethylenediaminetetraacetic acid. After 5 min, 10 mM dithiothreitol was added. After sonication and centrifugation, the suspension was mixed well with a 5-fold volume of chilled acetone containing 10% trichloroacetic acid and incubated overnight at −20°C. After centrifugation (4°C, 30,000 ×*g*), the precipitate was washed three times with chilled acetone. The pellet was air-dried and dissolved in lysis buffer B (7 M urea, 2 M thiourea, 4% nonylphenol ethoxylate (NP-40), and 20 mM Tris-HCl, pH 8.5). The suspension was sonicated for 15 min and centrifuged at 4°C and 30,000 ×*g* for 15 min. Subsequently, 10 mM dithiothreitol was added to reduce disulfide bonds in proteins in the supernatant, and the solution was incubated at 56°C for 1 h. Next, 55 mM iodoacetamide was added to bind to cysteines and the solution was incubated for 1 h in the dark. The supernatant was mixed well with a 5-fold volume of chilled acetone for 2 h at −20°C. After centrifugation (30,000 ×*g* for 20 min), the pellet was air-dried for 5 min, then dissolved in 500 μL of 0.5 M triethylammonium bicarbonate and sonicated for 15 min. Finally, after centrifugation at 4°C and 30,000 ×*g* for 15 min, the supernatant was transferred into a new tube and quantified by Bradford’s method [22]. The proteins in the supernatant were stored at −80°C for further analysis.

### iTRAQ labeling and chromatography fractionation

Total protein (100 μg) taken from each sample solution was digested with Trypsin Gold (Promega, USA) with a 20:1 ratio of protein:trypsin at 37°C for 4 h. After trypsinization and drying by vacuum centrifugation, peptides were redissolved using 0.5 M triethylammonium bicarbonate and iTRAQ reagent (Applied Biosystems, USA) according to the manufacturer’s instructions. Each group of peptides was marked by different iTRAQ tags and incubated at room temperature for 2 h. We mixed all groups of tagged peptides, purified them using a strong cation exchange chromatography column (Phenomenex, USA), and separated them by liquid chromatography (LC) using a LC-20AB high pressure LC pump system (Shimadzu, Japan). Then we redissolved tagged mixed peptides with 4 mL of buffer A (25 mM NaH_2_PO_4_ in 25% acetonitrile (ACN), pH 2.7) and loaded them onto a 4.6 × 250 mm Ultremex strong cation exchange column containing 5 mm particles (Phenomenex). Gradient elution was applied to peptides at a flow rate of 1 mL min^−1^, in which we initially used buffer A for 10 min elution and then progressively interfused 5–35% buffer B (25 mM NaH_2_PO_4_, 1 M KCl in 25% ACN, pH 2.7) for 11 min elution. Finally, we conducted 1 min elution with 35–80% buffer B. The entire elution process was monitored by measuring the absorbance at 214 nm, and each component was desalted with a Strata X C18 column and vacuum dried.

### LC-electrospray ionization-tandem mass spectrometry analysis

A nanoACQuity (Waters, USA) rapid separation LC system connected with the mass spectrometer, including a Symmetry C18 column (5 μm, 180 um × 20 mm), was used for peptide absorption and desalting, and a BEH130 C18 column (1.7 μm, 100 um × 100 mm) was used for separation. Both mobile phase buffer A (98:2:0.1 H_2_O:ACN:HCOOH) and buffer B (2:98:0.1 H_2_O:ACN:HCOOH) were added with a certain ratio of correction fluid (Thermo Fisher Scientific, USA). A 2.25 μg (9 μl) amount was loaded each time. Peptide absorption and desalting were carried out with buffer A at a flow rate of 2 μL min^−1^ for 15 min elution. The samples were loaded with 5% buffer B at 300 nL min^−1^ for 1 min, and then a 40 min gradient was run starting with 5–35% buffer B, followed by 5 min of linear gradient to 80%, followed by 5 min of maintenance at 80%, and a final 2 min at 5%.

Data were acquired with a TripleTOF 5600 System (AB SCIEX, Concord, ON) fitted with a Nanospray III source (AB SCIEX), with a pulled quartz tip as the emitter (New Objectives, Woburn, MA), controlled by the software program Analyst 1.6 (AB SCIEX). The following mass spectrometry conditions were used: 2.5 kV ion spray voltage, 30 psi curtain gas, 15 psi nebulizer gas, and 150°C interface heater temperature. The resolution was approximately 30,000. For independent data acquisition, survey scans were acquired in 250 ms and as many as 30 product ion scans were collected if they exceeded a threshold of 120 counts s^−1^ and had a 2+ to 5+ charge state. The total cycle time was fixed at 3.3 s. The second quadrupole transmission window was 100 Da for 100%. Four time bins were summed for each scan at a pulsed frequency value of 11 kHz through monitoring of a 40 GHz multichannel time-to-digital detector with four anode channels. An adjusted iTRAQ rolling collision energy was applied to all precursor ions for collision-induced dissociation. Dynamic exclusion was set for 1/2 of peak width (15 s), and then the precursor was refreshed off the exclusion list.

### Protein identification and bioinformatics analysis

Raw mass spectrum files were converted into Mascot generic files and protein identification was conducted using Mascot (version 2.3.02) to search for predicted proteins in the Eggplant Genome DataBase (http://eggplant.kazusa.or.jp/). For further functional analysis, differential expression of proteins was analyzed for significant downregulation or upregulation. A change in expression was determined by comparing the S-morph and L-morph flower pistils during two developmental stages, and *t*-tests were used to identify significant (*p* < 0.05) differences. The proteins with an average fold-change ≥ 1.5 or ≤ 0.667, and unique proteins with at least two peptide matches, were confidently defined as differentially expressed proteins (DEPs).

Proteins were classified by Gene Ontology (GO) analysis with AmiGO 2 (http://amigo.geneontology.org/amigo) based on three categories: biological process, cellular component, and molecular function. The Kyoto Encyclopedia of Genes and Genomes (KEGG) was used to annotate pathways in the KEGG pathway database (http://www.genome.jp/kegg/pathway.html) [23]. The statistical significance of GO terms or KEGG pathway enrichment was determined by a hypergeometric test following Yu *et al*.[24]. Additionally, protein-protein interaction networks for DEPs were explored using the publicly available Search Tool for the Retrieval of Interacting Genes/Proteins (STRING) database [25].

### Quantitative real-time PCR analysis

We selected genes from the DEPs of developing and mature flowers to validate the high throughput data at a transcriptional level by qRT-PCR. GAPDH (glyceraldehyde-3-phosphate dehydrogenase) was used as a reference gene and the primers used in this study are listed in S1 Table. We added 10 μL 2× Master Mix, 0.6 μL 10 μM forward primer, 0.6 μL 10 μM reverse primer, 0.8 μL complementary DNA (cDNA) template reverse transcripted to a PCR reaction system (Fermentas), and enough diethyl pyrocarbonate water for a total volume of 20 μL. A Stepone Plus thermocycler was used (Applied Biosystems, Foster City, CA). The amplification protocol consisted of 95°C denaturation for 10 min; 40 cycles of 95°C for 15 s, 60°C for 30 s, and 72°C for 15 s, and 95°C for 15 s, 60°C 1 min, and 95°C 15 s for a melting curve and PCR specificity test. The analysis was performed using two independent cDNA preparations in triplicate PCR reactions. The relative expression ratio was calculated using the 2^-ΔΔCt^ method [26] with eggplant *GAPDH* as the internal reference gene.

## Results

### Phenotypic differences between L-morph and S-morph flowers in eggplant

**Fig 1:**
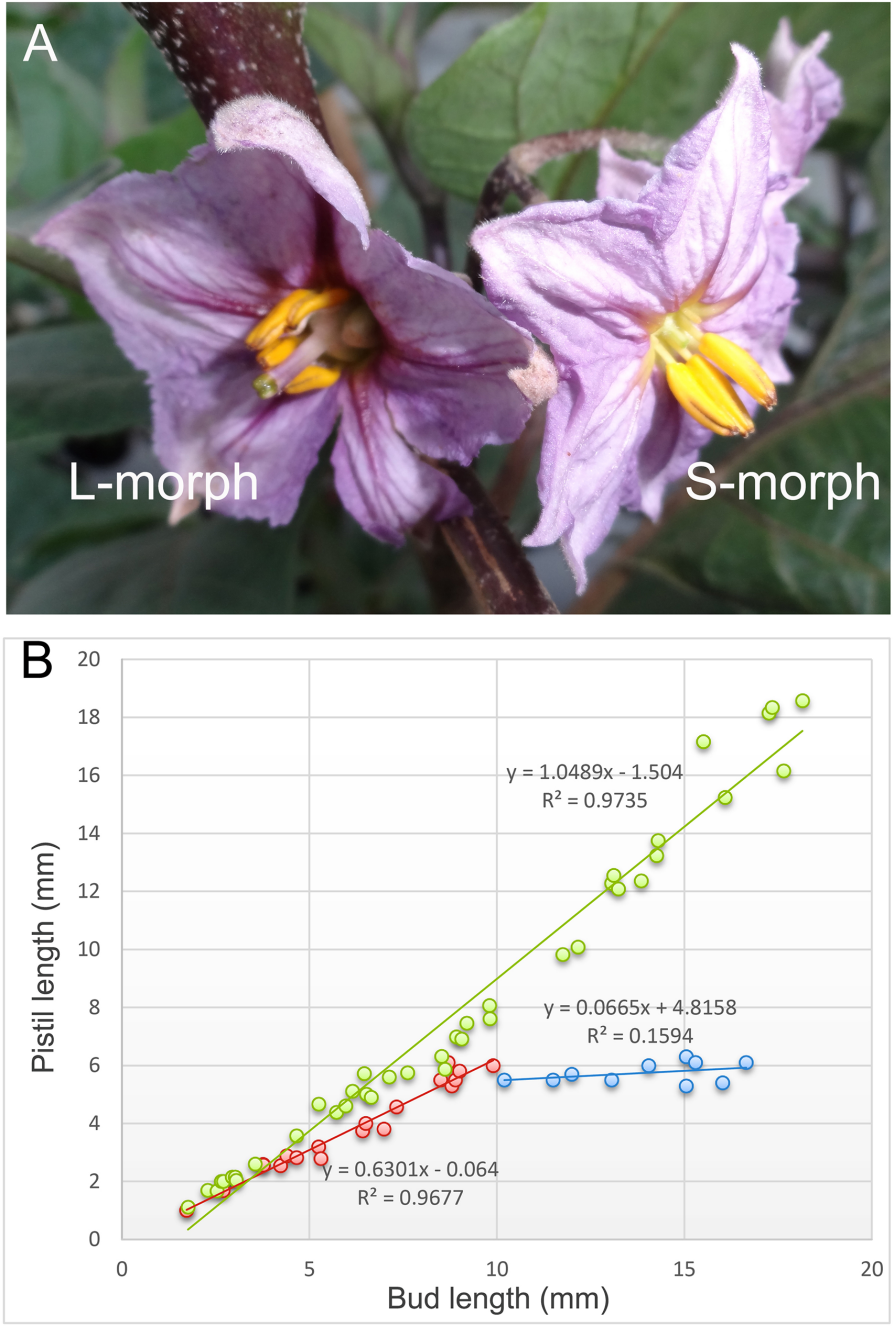
L-morph and S-morph flowers and correlation between pistil length and flower bud length during development. (A) Overview of L-morph and S-morph flowers. (B) The relationship between pistil length and flower bud in L-morph and S-morph flowers. The green dots indicate the relationship between bud length and pistil length in L-morph flowers. In S-morph flowers, the relationship is indicated in red when bud length < 10 mm and blue when bud length > 10 mm.

Heterostyly is a type of flower polymorphism that leads to separation of the stigma and anthers (hercogamy), preventing self-pollination, and is widely distributed in angiosperms [1,27,28]. In this study, two morphologically different types of flowers, L-morph and S-morph, were observed at different developmental stages in eggplant. As shown in Fig 1A, the flowers are clearly different, especially in their pistil length. During flower development, the pistil length increased linearly with flower bud length in L-morph flowers (R^2^ = 0.974) (Fig 1B). Although the S-morph flowers experienced similar growth when the pistil length was less than 10 mm (R^2^ = 0.968), the pistil generally did not elongate when the buds were more than 10 mm long (Fig 1B, indicated in blue). Accordingly, we divided the pistil development of S-morph flowers into two stages, development and maturity. The former stage was present until approximately 10 DAB, after which pistils were considered mature. During the maturity stage, the S-morph flowers were significantly different from the L-morph flowers, regardless of bud length.

### Characterization of self- and cross-pollination of L-morph and S-morph flowers

**Fig 2:**
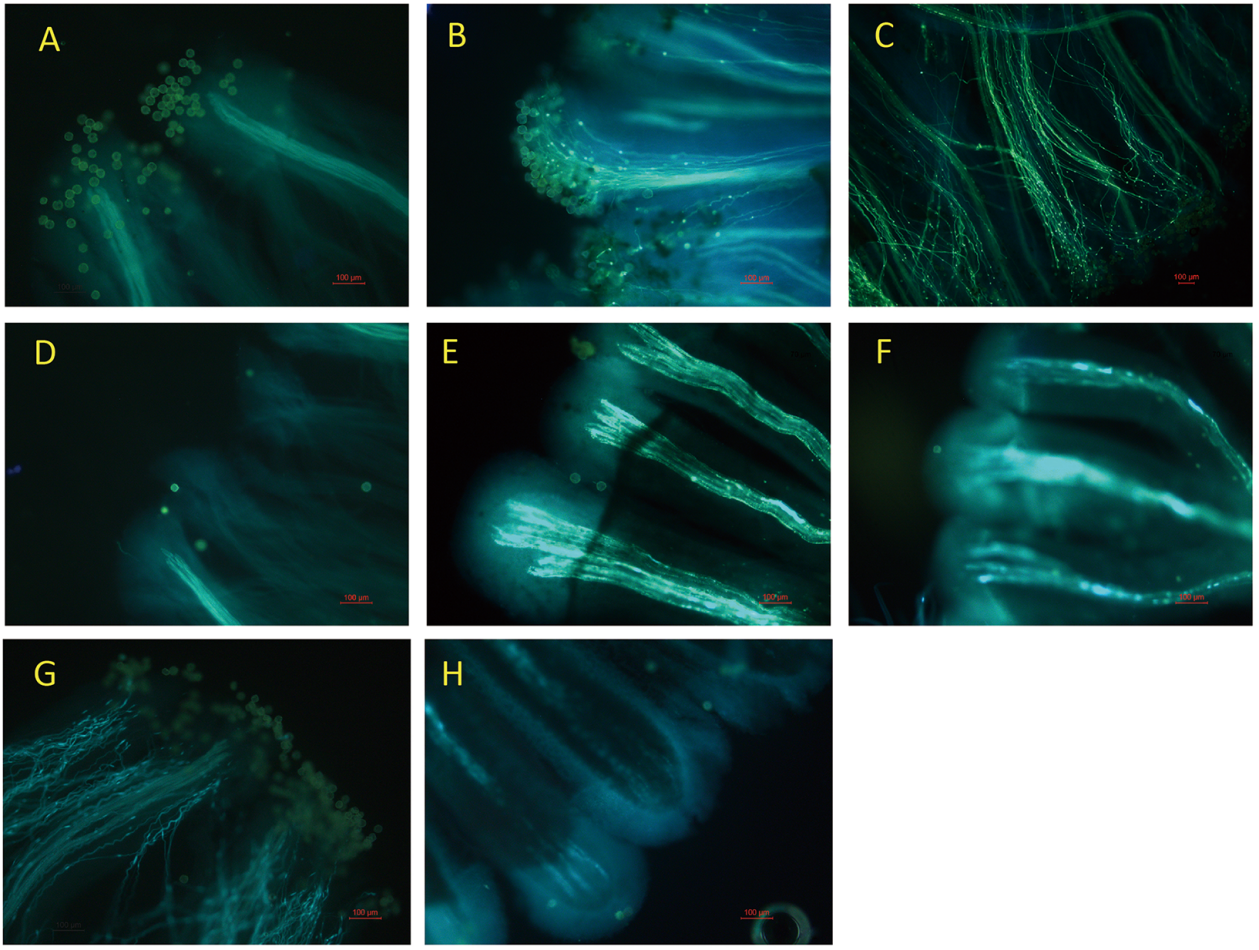
Microspore germination on stigmas after self- or cross-pollination. A, B and C show microspore germination on stigmas at 1 h, 4 h, or 24 h after L-morph flower self-pollination. D, E and F show microspore germination on stigmas at 1 h, 4 h, or 24 h after S-morph flower self-pollination. G show microspore germination on stigmas at 24 h after cross-pollination of S-morph pollen to L-morph stigma. H show microspore germination on stigmas at 24 h after cross-pollination of L-morph pollen to S-morph stigma. Scale bar = 100 μm.

Fig 2A shows that no obvious pollen germination occurred 1 h after self-pollination of L-morph flowers, but the pollen adhered well to the stigma. Pollen tubes germinated well 4 h after pollination (Fig 2B). With time, pollen tube elongation and germination increased (Fig 2C). However, after self-pollination of S-morph flowers, the pollen failed to germinate, and not as much pollen adhered to the stigma (Fig 2D, 2E and 2F), indicating incompatibility in self-pollination. Hence, compared with the L-morph flowers, pollen does not adhere as well to the stigma of S-morph flowers.

To determine whether the germination failure of pollen in S-morph flowers originated with the pollen or pistil, we reciprocally cross-pollinated L-morph and S-morph flowers. Germination occurred 24 h after cross-pollination of S-morph pollen to L-morph stigmas (Fig 2G). S-morph pollen germinated and pollen tubes grew normally on the stigmas of L-morph flowers, which suggested that of pollen from S-morph flowers can function normally. However, no obvious germination occurred 24 h after cross-pollination of L-morph pollen to S-morph stigmas (Fig 2H). No penetration of pollen tubes into the short style was detected, nor did any pollen grains germinate on the stigma. Given that the pollen of L-morph flowers can effectively germinate on L-morph flowers, this may indicate that the failure of pollen germination stems from the S-morph stigmas. Therefore, we inferred that the structure of the stigmatic surface in S-morph flowers may inhibit pollen germination, and the lower fruit set of S-morph flowers may reflect a shift toward functionally staminate flowers.

### Structural observations of pistils in L-morph and S-morph flowers

**Fig 3:**
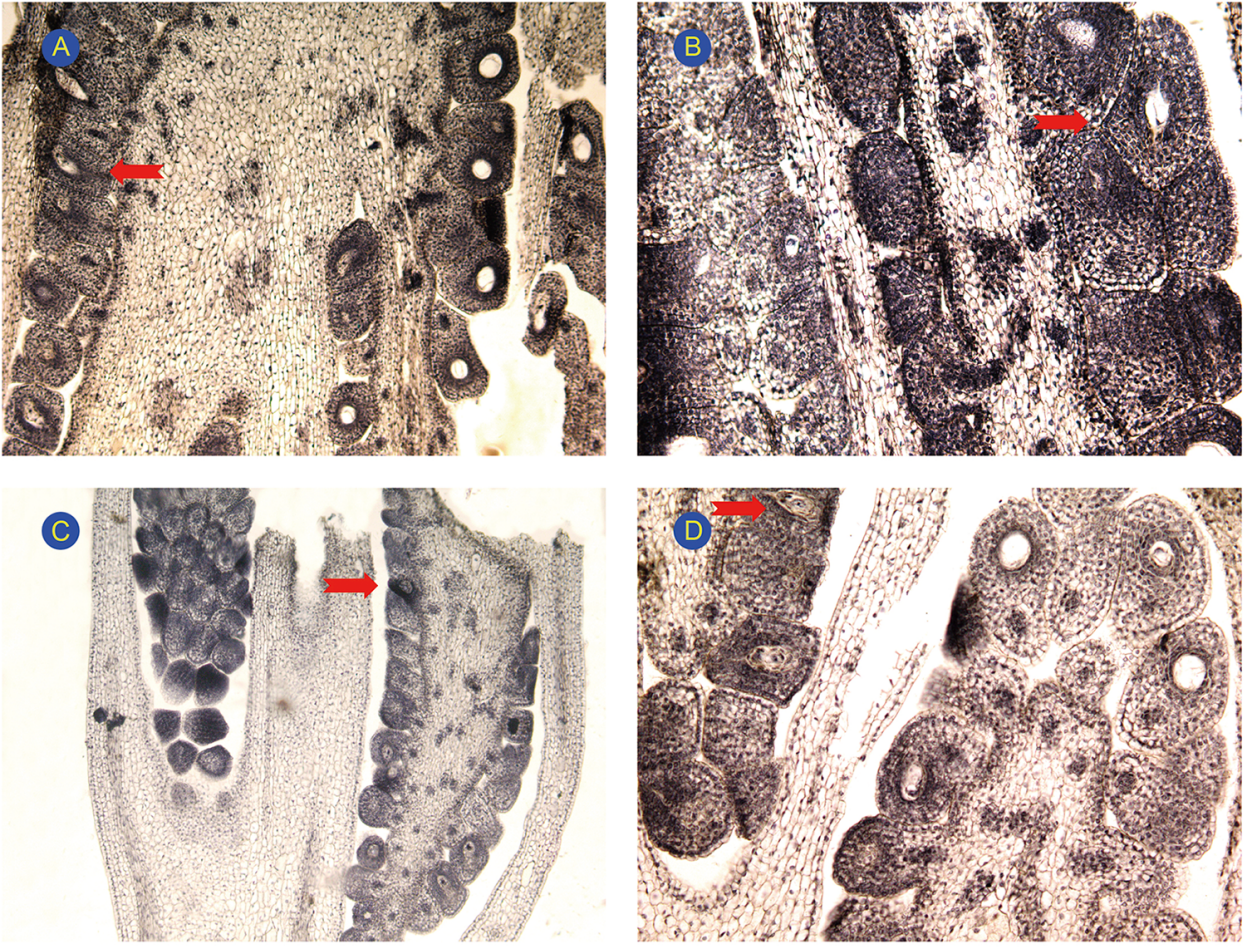
Observation of the pistil development in L-morph and S-morph flowers. (A) Central cells and disintegration of antipodal cells in maturing embryo sac of L-morph flower. (B) The two synergids in maturing embryo sac of L-morph flower. (C) Mitosis prophase of megasporocyte in S-morph flower. (D) The two synergids, one egg cell, and central cell in maturing embryo sac of S-morph flower.

We examined eggplant embryo sacs of both S-morph and L-morph flowers from budding to blooming to better understand early embryonic development and compare the structural integrity of egg cells, central cells, antipodal cells, and synergids (Fig 3). The egg cell and two synergids were located at the micropylar end of the nucellus. The central cell occupied the majority of the embryo sac. Three smaller antipodal cells were located at the chalazal end. The structure of embryo sacs from S-morph and L-morph flowers did not differ; we therefore inferred that low fertilization rates of S-morph flowers probably resulted from stigmatic incompatibility, rather than defects in the embryo sac.

### Protein identification overview

We conducted a comparative proteome survey using the iTRAQ technique to detect molecular expression differences between S-morph and L-morph flowers during heterostyly development. We identified 16,460 high-quality unique peptides from 346,625 secondary spectra. We searched the Eggplant Genome DataBase (http://eggplant.kazusa.or.jp/) [29] and compared the peptides with predicted proteins using Mascot 2.3.02, identifying 4,728 proteins (S2A Fig). We classified 16,460 peptides in 42 categories based on peptide length; the most frequent category (~10%) was 9–11 amino acids (S2B Fig). The majority of the 4,728 identified proteins included fewer than 10 peptides, and as the peptide number increased, the number of corresponding proteins decreased (S2C Fig). All the proteins with a false discovery rate (FDR) less than 1% were included in downstream analyses including (GO, Cluster of Orthologous Groups of proteins (COG) and KEGG Pathway).

COG is a database that is generated by comparing predicted and known proteins in all completely sequenced genomes to infer sets of orthologs. We compared proteins in our study with the COG database to predict possible functions and conduct a functional classification analysis (S3A Fig). The 4,728 identified proteins were categorized as involved in post-translational modification, protein turnover, chaperone functions, translation, ribosomal structure and biogenesis, carbohydrate metabolism and transport, energy production and conversion, and amino acid metabolism and transport. Additionally, many proteins (≥ 100) were involved in lipid transport and metabolism, transcription, replication, recombination and repair, signal transduction mechanisms, cell wall/membrane/envelope biogenesis, secondary metabolite biosynthesis, transport, and catabolism.

GO is a community-based bioinformatics resource that standardizes descriptions of functions and classifies gene product functions through the use of structured, controlled vocabularies for associated biological processes, cellular components, and molecular functions. As shown in S3B Fig, the highest percentages of GO terms related to biological processes were cellular progress, metabolism progress, and single-organism process, while those related to cellular components were cell, cell part, and organelle. The highest percentages of GO terms related to molecular functions were binding and catalytic activity.

The KEGG pathway database is a collection of pathway maps representing our knowledge of molecular interaction and reaction networks [23]. A total of 135 pathways were annotated for 3,380 of the 4,728 proteins. More than 5% of the identified proteins belonged to pathways of metabolism, biosynthesis of secondary metabolites, carbon metabolism, and biosynthesis of amino acids (S3C Fig).

### Analysis of protein expression patterns in L-morph and S-morph flowers during development and maturity

Here, we defined proteins with expression level fold changes > 1.5 and *p*-values < 0.05 as DEPs. We analyzed protein expression levels in the pistils of both L-morph and S-morph flowers during development (0–6 DAB) and discovered 24 downregulated and 33 upregulated proteins in S-morph flowers compared with L-morph flowers (Tables 1 and 2). Alanine-glyoxylate aminotransferase 2 (AGT2) homolog 3 (mitochondrial-like) had the greatest downregulation. AGT2 is a peroxisomal photorespiratory enzyme that catalyzes transamination reactions with multiple substrates [30]. Agamous-like MADS-box protein AGL61-like had the greatest upregulation. During maturity (13 DAB), 83 proteins were upregulated and 101 were downregulated in S-morph flowers compared with L-morph flowers (Tables 3 and 4). Polyubiquitin-like had the greatest downregulation. Interestingly, pistil extension-like protein (Sme2.5_02187.1_g00002.1) was downregulated in S-morph flowers during maturity, which may indicate that the short pistil of S-morph flowers is related to its lower expression level. Pectinesterase 2-like was the top differentially upregulated protein. Additionally, during flower maturity, the expression levels of cysteine proteinase and peroxidase, which are involved in senescence and programmed cell death [31], were upregulated in S-morph flowers, which might contribute to the failure of S-morph flowers to set fruit. We found that methionine sulfoxide reductase A3 in S-morph flowers, which is involved in the response to oxidative stress [32], was downregulated during both flower development and maturity compared with L-morph flowers. Moreover, the cysteine proteinase expression level increased in S-morph pistils and was probably related to cell apoptosis.

**Table 1:** Upregulated proteins in pistils of S-morph flowers with a 1.5-fold change compared with L-morph flowers during development.

Protein_ID	Description	Mass	Coverage	Peptide	Fold change	Qvalue
Sme2.5_03854.1_g00004.1	uncharacterized protein LOC543817	25718.33	0.037	1	2.974	0.047
Sme2.5_04037.1_g00003.1	glutelin type-A 2-like	28656.76	0.342	2	1.546	0.002
Sme2.5_06391.1_g00003.1	cyclic nucleotide-gated ion channel 1-like	13069.66	0.088	1	3.793	0.035
Sme2.5_05614.1_g00005.1	lysosomal beta glucosidase-like	37057.35	0.226	5	1.557	0.002
Sme2.5_13401.1_g00002.1	uncharacterized protein At1g47420, mitochondrial-like isoform 2	31502.39	0.301	1	3.326	0.002
Sme2.5_04699.1_g00005.1	profilin-1-like	17863.69	0.390	4	2.005	0.050
Sme2.5_00368.1_g00010.1	4-coumarate—CoA ligase-like 1-like	61656.04	0.215	9	1.587	0.002
Sme2.5_14644.1_g00002.1	predicted protein	12132.46	0.339	3	2.163	0.002
Sme2.5_09858.1_g00002.1	ribulose-1,5-bisphosphate carboxylase/oxygenase large subunit	21021.44	0.074	1	1.552	0.006
Sme2.5_00346.1_g00019.1	chalcone synthase-like	44007.60	0.295	8	1.528	0.016
Sme2.5_01611.1_g00010.1	H1 histone-like protein	21647.19	0.095	2	1.861	0.003
Sme2.5_00572.1_g00010.1	alpha-DOX2	63593.44	0.034	2	1.607	0.028
Sme2.5_12240.1_g00001.1	uncharacterized protein LOC101306013	114772.3	0.028	2	4.052	0.017
Sme2.5_04401.1_g00002.1	protein HOTHEAD-like	54469.65	0.216	8	1.985	0.002
Sme2.5_08304.1_g00001.1	profilin-1	14120.02	0.546	5	1.789	0.002
Sme2.5_07601.1_g00002.1	hypothetical protein VITISV_035070	47141.44	0.017	1	1.512	0.002
Sme2.5_04937.1_g00002.1	agamous-like MADS-box protein AGL61-like	13234.40	0.068	1	7.349	0.002
Sme2.5_03454.1_g00001.1	probable sarcosine oxidase-like	45726.07	0.169	6	1.971	0.003
Sme2.5_00048.1_g00028.1	uncharacterized protein LOC101250105	63267.26	0.227	12	1.661	0.002
Sme2.5_04309.1_g00005.1	HMG1/2-like protein-like isoform 2	15804.77	0.486	2	1.592	0.012
Sme2.5_03497.1_g00004.1	uncharacterized protein LOC101243982	21283.66	0.043	1	1.937	0.006
Sme2.5_04891.1_g00002.1	tetraketide alpha-pyrone reductase 2-like	30261.27	0.078	2	1.686	0.002
Sme2.5_07446.1_g00003.1	ribulose-1,5-bisphosphate carboxylase/oxygenase large subunit	21375.91	0.117	1	1.585	0.005
Sme2.5_03231.1_g00008.1	Ribulose bisphosphate carboxylase small chain 8B	20647.18	0.494	1	1.524	0.005
Sme2.5_00468.1_g00005.1	GDSL esterase/lipase At2g31540-like	39023.43	0.038	1	3.477	0.043
Sme2.5_04260.1_g00006.1	heterodimeric geranylgeranyl pyrophosphate synthase small subunit, chloroplastic-like isoform 1	34534.52	0.166	4	1.580	0.002
Sme2.5_05063.1_g00001.1	polygalacturonase QRT3-like	104455.70	0.198	15	1.976	0.002
Sme2.5_04696.1_g00006.1	subtilisin-like protease-like	65871.21	0.191	10	1.787	0.002
Sme2.5_21483.1_g00001.1	cysteine-rich receptor-like protein kinase 10-like	40578.25	0.019	1	1.756	0.010
Sme2.5_06878.1_g00001.1	cytochrome P450 704C1-like	59002.25	0.060	3	1.800	0.002
Sme2.5_00049.1_g00003.1	early nodulin-like protein 1-like	18748.54	0.179	2	2.689	0.006
Sme2.5_09669.1_g00005.1	beta-glucosidase 40-like	57747.89	0.222	11	2.028	0.002
Sme2.5_31247.1_g00001.1	hypothetical protein VITISV_027379	23536.02	0.173	1	1.709	0.023

**Table 2:** Downregulated proteins in pistils of S-morph flowers with a 1.5-fold change compared with L-morph flowers during development.

Protein_ID	Description	Mass	Coverage	Peptide	Fold change	Qvalue
Sme2.5_03906.1_g00010.1	bifunctional monodehydroascorbate reductase and carbonic anhydrase nectarin-3-like	29618.23	0.110	1	0.400	0.003
Sme2.5_24823.1_g00001.1	uncharacterized protein At1g47420, mitochondrial-like isoform 2	24316.70	0.388	1	0.501	0.008
Sme2.5_03184.1_g00003.1	Putative gag-pol polyprotein, identical	173316.10	0.009	1	0.149	0.020
Sme2.5_04111.1_g00005.1	citrate binding protein	21111.52	0.228	4	0.621	0.002
Sme2.5_04984.1_g00003.1	proteinase inhibitor II	25168.62	0.333	5	0.576	0.002
Sme2.5_10015.1_g00002.1	basic 30 kDa endochitinase-like	38015.66	0.162	3	0.556	0.002
Sme2.5_03742.1_g00003.1	Glycosyl hydrolases family 17 protein	44096.32	0.087	3	0.663	0.006
Sme2.5_08975.1_g00004.1	uncharacterized protein LOC101252371	94859.44	0.011	1	0.291	0.048
Sme2.5_00235.1_g00003.1	ribonuclease 3-like	24947.69	0.156	4	0.642	0.002
Sme2.5_02047.1_g00009.1	miraculin-like, partial	23253.65	0.103	2	0.623	0.002
Sme2.5_01085.1_g00002.1	probable leucine-rich repeat receptor-like protein kinase At1g35710-like	71005.38	0.224	11	0.656	0.002
Sme2.5_00512.1_g00007.1	bifunctional monodehydroascorbate reductase and carbonic anhydrase nectarin-3-like	28298.46	0.586	9	0.562	0.002
Sme2.5_00275.1_g00003.1	desiccation-related protein PCC13-62-like	34154.37	0.070	2	0.617	0.021
Sme2.5_00345.1_g00027.1	alanine—glyoxylate aminotransferase 2 homolog 3, mitochondrial-like	28097.52	0.044	1	0.100	0.013
Sme2.5_08282.1_g00001.1	DNA-damage-repair/toleration protein DRT100-like	40864.38	0.198	6	0.450	0.002
Sme2.5_00008.1_g00037.1	probable inactive receptor kinase At1g48480-like	70601.67	0.131	5	0.489	0.014
Sme2.5_02193.1_g00001.1	cysteine protease inhibitor 8-like	34677.99	0.144	3	0.423	0.002
Sme2.5_31478.1_g00001.1	acidic 27 kDa endochitinase precursor	18447.83	0.182	1	0.555	0.008
Sme2.5_28714.1_g00002.1	class II chitinase	27594.14	0.268	3	0.550	0.002
Sme2.5_03276.1_g00004.1	trypsin proteinase inhibitor precursor	25387.60	0.330	5	0.573	0.002
Sme2.5_01810.1_g00004.1	ubiquilin-2-like	35661.56	0.061	1	0.281	0.047
Sme2.5_00001.1_g00016.1	wound-inducible carboxypeptidase precursor	50648.16	0.052	2	0.664	0.002
Sme2.5_07601.1_g00001.1	methionine sulfoxide reductase A3	35147.93	0.030	1	0.455	0.013
Sme2.5_00745.1_g00004.1	peroxidase 17-like	35460.50	0.280	8	0.542	0.002

**Table 3:** Upregulated proteins in pistils of S-morph flowers with a 1.5-fold change compared with L-morph flowers during maturity.

Protein_ID	Description	Mass	Coverage	Peptide	Fold change	Qvalue
Sme2.5_02083.1_g00006.1	putative pectinesterase/pectinesterase inhibitor 28-like	60660.48	0.078	4	3.158	0.001
Sme2.5_00076.1_g00003.1	leucine-rich repeat extensin-like protein 3-like	56883.38	0.064	3	1.630	0.003
Sme2.5_00016.1_g00015.1	hypothetical protein VITISV_019164	13394.11	0.234	2	1.921	0.001
Sme2.5_00188.1_g00008.1	ATP synthase subunit delta', mitochondrial-like isoform 1	14509.35	0.188	2	1.607	0.001
Sme2.5_02533.1_g00006.1	olee1-like protein-like	19924.59	0.114	2	5.121	0.001
Sme2.5_00086.1_g00012.1	SlArf/Xyl3	69479.43	0.198	9	3.777	0.001
Sme2.5_05092.1_g00005.1	fasciclin-like arabinogalactan protein 14-like	18313.54	0.124	2	2.038	0.001
Sme2.5_00944.1_g00019.1	Blue copper protein precursor, putative	25228.61	0.071	1	1.510	0.001
Sme2.5_05878.1_g00004.1	unknown	27148.47	0.029	1	2.164	0.001
Sme2.5_06660.1_g00003.1	uncharacterized protein LOC101266493 isoform 1	20375.65	0.173	3	2.523	0.001
Sme2.5_00377.1_g00016.1	subtilisin-like protease-like	83322.67	0.147	10	1.527	0.001
Sme2.5_03383.1_g00005.1	late embryogenesis abundant protein 1-like	9375.45	0.409	2	4.441	0.001
Sme2.5_05238.1_g00003.1	unknown	16150.91	0.071	1	2.397	0.001
Sme2.5_02369.1_g00001.1	peroxidase N-like isoform 1	29230.45	0.151	2	1.855	0.038
Sme2.5_06507.1_g00004.1	serine carboxypeptidase-like 45-like	57255.26	0.032	1	3.267	0.003
Sme2.5_00817.1_g00004.1	endonuclease 2-like	32347.73	0.283	6	1.657	0.001
Sme2.5_05614.1_g00005.1	lysosomal beta glucosidase-like	37057.35	0.226	5	2.105	0.001
Sme2.5_07124.1_g00003.1	beta-D-xylosidase 1 precursor	84376.62	0.081	4	2.834	0.001
Sme2.5_07880.1_g00001.1	glucan endo-1,3-beta-glucosidase 8-like	85413.96	0.037	3	3.656	0.001
Sme2.5_01196.1_g00005.1	ribokinase-like	43914.26	0.098	3	1.611	0.004
Sme2.5_13401.1_g00002.1	uncharacterized protein At1g47420, mitochondrial-like isoform 2	31502.39	0.301	1	5.725	0.001
Sme2.5_03722.1_g00006.1	anther-specific protein LAT52-like	18412.04	0.057	1	6.641	0.001
Sme2.5_04699.1_g00005.1	profilin-1-like	17863.69	0.390	4	2.996	0.001
Sme2.5_06227.1_g00005.1	late embryogenesis abundant protein D-34-like	22191.38	0.611	10	1.554	0.001
Sme2.5_01431.1_g00003.1	Putative retrotransposon protein, identical	86414.53	0.044	3	1.980	0.001
Sme2.5_05048.1_g00002.1	uncharacterized protein LOC101265833	9975.86	0.182	1	2.263	0.001
Sme2.5_07288.1_g00002.1	UMP/CMP kinase-like	22910.57	0.236	4	1.682	0.001
Sme2.5_02324.1_g00010.1	GDSL esterase/lipase At4g01130-like	40249.09	0.091	3	1.751	0.020
Sme2.5_04547.1_g00002.1	probable LRR receptor-like serine/threonine-protein kinase At1g06840-like	106411.20	0.040	3	1.508	0.001
Sme2.5_00701.1_g00012.1	germin-like protein subfamily 1 member 15-like	24697.72	0.507	7	1.626	0.001
Sme2.5_12877.1_g00001.1	subtilisin-like protease precursor	112544.00	0.101	5	1.502	0.004
Sme2.5_00223.1_g00004.1	polygalacturonase-like	72678.90	0.078	4	4.050	0.011
Sme2.5_02955.1_g00005.1	unknown	38980.04	0.623	9	1.640	0.001
Sme2.5_05245.1_g00001.1	uncharacterized protein LOC101249738	37417.26	0.074	2	4.382	0.001
Sme2.5_00827.1_g00004.1	uncharacterized protein At4g13230-like	13245.75	0.107	1	2.598	0.002
Sme2.5_01764.1_g00007.1	GDSL esterase/lipase APG-like	39003.76	0.312	7	1.712	0.001
Sme2.5_03252.1_g00002.1	probable polygalacturonase-like	49657.64	0.051	2	5.815	0.001
Sme2.5_14644.1_g00002.1	predicted protein	12132.46	0.339	3	2.409	0.001
Sme2.5_00588.1_g00013.1	uncharacterized protein LOC101253861	27398.90	0.232	4	1.517	0.049
Sme2.5_00015.1_g00020.1	flavanone 3-hydroxylase	41376.09	0.331	9	1.680	0.001
Sme2.5_14501.1_g00004.1	uncharacterized protein At5g39570-like	26376.08	0.096	2	2.237	0.001
Sme2.5_04720.1_g00004.1	GDSL esterase/lipase At1g29670-like	37336.80	0.024	1	6.425	0.036
Sme2.5_02824.1_g00005.1	Anther-specific protein LAT52	18507.87	0.180	3	5.440	0.002
Sme2.5_00188.1_g00003.1	pectinesterase 2-like	41033.75	0.128	2	7.812	0.001
Sme2.5_07102.1_g00002.1	unknown	20846.97	0.077	1	1.570	0.024
Sme2.5_02098.1_g00007.1	uncharacterized protein LOC101243814 isoform 1	16188.35	0.140	1	1.959	0.006
Sme2.5_02947.1_g00002.1	uncharacterized protein LOC101267484	96934.38	0.014	1	1.640	0.004
Sme2.5_00864.1_g00008.1	oryzain alpha chain-like	20763.01	0.364	5	1.525	0.001
Sme2.5_08304.1_g00001.1	profilin-1	14120.02	0.546	5	2.950	0.001
Sme2.5_19379.1_g00002.1	non-specific lipid-transfer protein 2-like isoform 1	8960.47	0.171	2	2.615	0.001
Sme2.5_00041.1_g00026.1	non-specific lipid-transfer protein-like protein At2g13820-like	17325.40	0.048	1	1.987	0.008
Sme2.5_12729.1_g00004.1	lysosomal beta glucosidase-like	66678.06	0.121	5	2.821	0.001
Sme2.5_01937.1_g00005.1	uncharacterized protein LOC101247575	80431.09	0.334	18	1.523	0.001
Sme2.5_00086.1_g00011.1	SlArf/Xyl3	84328.53	0.245	12	1.618	0.001
Sme2.5_01618.1_g00012.1	probable pectinesterase/pectinesterase inhibitor 51-like	59777.45	0.082	2	1.824	0.049
Sme2.5_05314.1_g00003.1	Putative gag-pol polyprotein, identical	116417.80	0.005	1	2.118	0.029
Sme2.5_00768.1_g00018.1	cysteine proteinase 3-like	40377.92	0.253	8	2.372	0.001
Sme2.5_03583.1_g00006.1	brassinosteroid-regulated protein BRU1	29952.77	0.086	1	2.474	0.002
Sme2.5_00740.1_g00010.1	denticleless protein homolog A-like	167057.90	0.042	6	1.534	0.036
Sme2.5_08226.1_g00002.1	glycine-rich RNA-binding protein-like	15970.37	0.362	3	1.560	0.001
Sme2.5_00225.1_g00038.1	somatic embryogenesis receptor kinase 3B precursor	64125.90	0.040	2	1.572	0.012
Sme2.5_00188.1_g00007.1	ATP synthase subunit delta', mitochondrial-like isoform 1	14320.49	0.272	2	1.684	0.001
Sme2.5_00019.1_g00028.1	vicilin-like antimicrobial peptides 2-2-like	51626.04	0.236	9	1.693	0.001
Sme2.5_01638.1_g00005.1	anthocyanin synthase	47089.86	0.151	4	1.715	0.006
Sme2.5_04773.1_g00002.1	uncharacterized protein LOC101263984	17506.54	0.213	3	1.566	0.001
Sme2.5_05293.1_g00002.1	L-ascorbate oxidase homolog isoform 1	62790.74	0.155	6	2.669	0.001
Sme2.5_00915.1_g00003.1	peptidyl-prolyl cis-trans isomerase-like	18253.00	0.390	4	1.886	0.001
Sme2.5_00188.1_g00004.1	LOW QUALITY PROTEIN: pectinesterase 1-like	34995.54	0.200	3	4.202	0.001
Sme2.5_02047.1_g00006.1	Kunitz-type enzyme inhibitor S9C11	23565.76	0.310	5	1.553	0.001
Sme2.5_06455.1_g00005.1	anther-specific protein LAT52-like	19151.32	0.214	3	4.474	0.002
Sme2.5_10801.1_g00001.1	UTP—glucose-1-phosphate uridylyltransferase-like	47977.96	0.394	9	1.638	0.001
Sme2.5_21483.1_g00001.1	cysteine-rich receptor-like protein kinase 10-like	40578.25	0.019	1	2.946	0.002
Sme2.5_04696.1_g00001.1	expansin11 precursor	28233.41	0.156	4	1.544	0.001
Sme2.5_10874.1_g00002.1	uncharacterized protein At5g39570-like	34605.10	0.951	19	2.100	0.001
Sme2.5_02148.1_g00009.1	UDP-glycosyltransferase 75D1-like	48331.87	0.167	6	1.658	0.001
Sme2.5_00170.1_g00013.1	uncharacterized protein LOC101251668	14895.89	0.104	1	2.611	0.002
Sme2.5_10869.1_g00001.1	uncharacterized protein LOC101258533	6962.28	0.273	1	3.316	0.003
Sme2.5_26344.1_g00001.1	uncharacterized protein LOC101260800	55494.69	0.042	2	2.110	0.001
Sme2.5_00100.1_g00024.1	polygalacturonase inhibiting protein	119649.20	0.172	16	1.630	0.001
Sme2.5_02584.1_g00008.1	8-hydroxygeraniol dehydrogenase	39249.74	0.231	3	1.585	0.012
Sme2.5_31247.1_g00001.1	hypothetical protein VITISV_027379	23536.02	0.173	1	3.330	0.001
Sme2.5_00813.1_g00013.1	uncharacterized protein LOC101258525	92237.69	0.035	3	1.696	0.009
Sme2.5_24838.1_g00001.1	methionine sulfoxide reducatase	9728.64	0.429	3	2.178	0.005

**Table 4:** Downregulated proteins in pistils of S-morph flowers with a 1.5-fold change compared with L-morph flowers during maturity.

Protein_ID	Description	Mass	Coverage	Peptide	Fold change	Qvalue
Sme2.5_01984.1_g00005.1	uncharacterized protein LOC100259659	20913.66	0.166	2	0.467	0.034
Sme2.5_00179.1_g00004.1	probable protein phosphatase 2C 27-like	67361.47	0.151	8	0.596	0.006
Sme2.5_00758.1_g00014.1	bifunctional purple acid phosphatase 26-like	53891.56	0.197	6	0.527	0.002
Sme2.5_30554.1_g00001.1	cell wall peroxidase	15930.86	0.083	1	0.430	0.006
Sme2.5_00676.1_g00001.1	60S ribosomal protein L7-4-like	27859.18	0.396	2	0.568	0.007
Sme2.5_12039.1_g00002.1	uncharacterized protein At5g01610-like isoform 2	23425.97	0.100	2	0.617	0.019
Sme2.5_24823.1_g00001.1	uncharacterized protein At1g47420, mitochondrial-like isoform 2	24316.70	0.388	1	0.431	0.017
Sme2.5_03582.1_g00003.1	proliferation-associated protein 2G4-like	44958.26	0.282	7	0.499	0.001
Sme2.5_21139.1_g00001.1	uncharacterized protein LOC101257658	47536.02	0.039	1	0.513	0.028
Sme2.5_01559.1_g00002.1	Histone H1	30859.06	0.268	6	0.371	0.001
Sme2.5_00343.1_g00001.1	40S ribosomal protein S28-like isoform 1	14716.98	0.094	1	0.659	0.006
Sme2.5_30393.1_g00001.1	predicted protein	9834.22	0.182	2	0.407	0.011
Sme2.5_01689.1_g00009.1	LEA1-like protein	21992.52	0.107	2	0.620	0.015
Sme2.5_01918.1_g00003.1	40S ribosomal protein S15-like	17241.40	0.318	3	0.539	0.004
Sme2.5_03836.1_g00005.1	ubiquitin extension protein	17859.54	0.391	1	0.280	0.018
Sme2.5_06391.1_g00003.1	cyclic nucleotide-gated ion channel 1-like	13069.66	0.088	1	0.183	0.046
Sme2.5_02104.1_g00006.1	uncharacterized protein LOC101260453	21674.39	0.111	1	0.391	0.001
Sme2.5_00097.1_g00005.1	methionine synthase	84904.88	0.656	33	0.646	0.001
Sme2.5_01494.1_g00003.1	60S ribosomal protein L19-2-like	24901.74	0.299	2	0.646	0.027
Sme2.5_00026.1_g00018.1	ribosomal protein PETRP-like	15530.16	0.422	3	0.658	0.001
Sme2.5_00001.1_g00048.1	cinnamic acid 4-hydroxylase	58331.85	0.244	12	0.640	0.001
Sme2.5_09948.1_g00002.1	60S ribosomal protein L27a-3-like	16468.95	0.236	2	0.663	0.027
Sme2.5_00079.1_g00001.1	uncharacterized protein LOC101244722	21014.85	0.219	3	0.561	0.007
Sme2.5_07653.1_g00001.1	Threonine dehydratase biosynthetic, chloroplastic	67242.71	0.444	17	0.607	0.001
Sme2.5_02268.1_g00004.1	60S ribosomal protein L21-2-like	18730.14	0.354	1	0.549	0.005
Sme2.5_01701.1_g00006.1	6,7-dimethyl-8-ribityllumazine synthase, chloroplastic-like	25211.79	0.129	2	0.662	0.006
Sme2.5_00026.1_g00001.1	glyceraldehyde 3-phosphate dehydrogenase	38609.81	0.521	5	0.475	0.001
Sme2.5_01674.1_g00010.1	apoplastic invertase	58606.65	0.109	4	0.661	0.004
Sme2.5_23355.1_g00001.1	Probable linoleate 9S-lipoxygenase 4	79251.74	0.717	30	0.628	0.001
Sme2.5_00065.1_g00022.1	1,2-dihydroxy-3-keto-5-methylthiopentene dioxygenase 2-like	23574.49	0.250	3	0.662	0.031
Sme2.5_00942.1_g00003.1	ribosomal protein S14-like protein	16373.64	0.367	1	0.512	0.001
Sme2.5_03872.1_g00005.1	uncharacterized protein LOC101245049	40572.39	0.122	4	0.600	0.005
Sme2.5_00310.1_g00014.1	60S ribosomal protein L27a-3-like	16430.90	0.372	4	0.573	0.001
Sme2.5_06364.1_g00001.1	60S ribosomal protein L34-like isoform 1	15618.94	0.294	2	0.652	0.006
Sme2.5_02784.1_g00004.1	unknown	11296.75	0.118	1	0.122	0.032
Sme2.5_00401.1_g00004.1	thioredoxin H	15726.95	0.176	2	0.641	0.014
Sme2.5_00281.1_g00013.1	ribosomal protein L3	44797.00	0.393	8	0.534	0.003
Sme2.5_00265.1_g00010.1	40S ribosomal protein S25-2-like	11914.60	0.361	4	0.423	0.001
Sme2.5_00606.1_g00004.1	dehydrin-like protein	24181.16	0.563	8	0.623	0.001
Sme2.5_02632.1_g00002.1	40S ribosomal protein S9-2-like	23011.27	0.431	4	0.645	0.039
Sme2.5_00108.1_g00014.1	ly200 protein	15727.59	0.135	1	0.413	0.003
Sme2.5_06588.1_g00003.1	60S ribosomal protein L9-1-like	37687.69	0.473	6	0.657	0.001
Sme2.5_08282.1_g00001.1	DNA-damage-repair/toleration protein DRT100-like	40864.38	0.198	6	0.215	0.001
Sme2.5_13015.1_g00001.1	allene oxide cyclase	27112.76	0.125	1	0.468	0.009
Sme2.5_00162.1_g00020.1	threonine deaminase, partial	40661.78	0.445	3	0.591	0.001
Sme2.5_02984.1_g00002.1	polyubiquitin-like	44423.86	0.016	1	0.100	0.017
Sme2.5_00008.1_g00037.1	probable inactive receptor kinase At1g48480-like	70601.67	0.131	5	0.318	0.046
Sme2.5_11776.1_g00001.1	polyphenol oxidase F, chloroplastic-like	62932.33	0.093	2	0.586	0.016
Sme2.5_15018.1_g00001.1	Gag-pol protein, putative	66167.90	0.010	1	0.574	0.004
Sme2.5_12240.1_g00001.1	uncharacterized protein LOC101306013	114772.30	0.028	2	0.381	0.002
Sme2.5_05365.1_g00004.1	cytochrome c1-1, heme protein, mitochondrial-like	35772.94	0.317	2	0.598	0.013
Sme2.5_00118.1_g00007.1	alpha-glucosidase	61176.01	0.117	5	0.576	0.001
Sme2.5_09773.1_g00001.1	pectin methyl esterase	63762.58	0.261	11	0.581	0.001
Sme2.5_01136.1_g00003.1	ADP/ATP translocator-like	50164.96	0.248	5	0.653	0.001
Sme2.5_02193.1_g00001.1	cysteine protease inhibitor 8-like	34677.99	0.144	3	0.103	0.001
Sme2.5_01374.1_g00009.1	cytoplasmic ribosomal protein S13-like	18548.42	0.472	7	0.659	0.001
Sme2.5_03722.1_g00005.1	phosphoenolpyruvate carboxylase	106816.00	0.288	4	0.616	0.015
Sme2.5_25992.1_g00001.1	chloroplast polyphenol oxidase precursor	65196.35	0.403	16	0.518	0.001
Sme2.5_00594.1_g00001.1	60S ribosomal protein L10	24698.94	0.250	3	0.495	0.013
Sme2.5_00151.1_g00009.1	unnamed protein product	12388.67	0.142	2	0.197	0.015
Sme2.5_00396.1_g00018.1	serine/arginine-rich splicing factor RS2Z32-like isoform 1	40515.85	0.093	3	0.661	0.006
Sme2.5_00584.1_g00002.1	30S ribosomal protein S31, chloroplastic-like	11783.15	0.232	3	0.615	0.001
Sme2.5_00341.1_g00020.1	glutathione reductase, cytosolic	109158.20	0.091	6	0.653	0.006
Sme2.5_02308.1_g00006.1	40S ribosomal protein S26-2-like	15000.93	0.070	1	0.453	0.001
Sme2.5_09935.1_g00001.1	ribosome biogenesis regulatory protein homolog	61521.38	0.040	2	0.621	0.015
Sme2.5_00125.1_g00003.1	calnexin-like protein precursor	61569.78	0.571	23	0.588	0.001
Sme2.5_08981.1_g00001.1	RNase Phy3, partial	56609.13	0.067	3	0.168	0.032
Sme2.5_00183.1_g00014.1	proliferation-associated protein 2G4-like	43133.41	0.238	5	0.539	0.001
Sme2.5_04572.1_g00005.1	protein GPR107-like	49965.33	0.016	1	0.533	0.005
Sme2.5_00940.1_g00015.1	40S ribosomal protein S16-like	22450.26	0.266	2	0.597	0.021
Sme2.5_15806.1_g00001.1	40S ribosomal protein S16-like isoform 1	16792.15	0.374	2	0.629	0.020
Sme2.5_01826.1_g00003.1	ferredoxin—NADP reductase, root-type isozyme, chloroplastic-like	26544.13	0.462	3	0.607	0.011
Sme2.5_00423.1_g00008.1	ribosomal protein L11-like protein	20931.98	0.326	6	0.664	0.001
Sme2.5_00411.1_g00010.1	translation machinery-associated protein 22-like isoform 1	22542.69	0.176	3	0.625	0.016
Sme2.5_05142.1_g00002.1	sucrose synthase-like	91913.37	0.308	15	0.598	0.001
Sme2.5_04982.1_g00006.1	protein ASPARTIC PROTEASE IN GUARD CELL 1-like	52387.73	0.093	5	0.641	0.001
Sme2.5_00048.1_g00028.1	uncharacterized protein LOC101250105	63267.26	0.227	12	0.633	0.001
Sme2.5_00043.1_g00021.1	hypothetical protein ZEAMMB73_313798	11384.39	0.573	4	0.609	0.001
Sme2.5_00088.1_g00019.1	40S ribosomal protein S3a-like	33114.47	0.463	5	0.348	0.047
Sme2.5_04309.1_g00005.1	HMG1/2-like protein-like isoform 2	15804.77	0.486	2	0.131	0.009
Sme2.5_10467.1_g00001.1	histone H3	15100.29	0.173	1	0.449	0.001
Sme2.5_00188.1_g00020.1	chalcone—flavonone isomerase-like	23248.80	0.289	4	0.616	0.016
Sme2.5_01635.1_g00012.1	proline iminopeptidase-like	28944.77	0.081	3	0.589	0.011
Sme2.5_02187.1_g00002.1	pistil-specific extensin-like protein	48531.74	0.088	2	0.257	0.001
Sme2.5_00014.1_g00016.1	histone H1	28785.72	0.309	7	0.377	0.001
Sme2.5_00075.1_g00019.1	unnamed protein product	109506.70	0.018	1	0.385	0.006
Sme2.5_01984.1_g00017.1	uncharacterized protein LOC100793233	12077.29	0.087	1	0.500	0.024
Sme2.5_02192.1_g00004.1	histone H2A-like protein	15549.89	0.264	2	0.631	0.039
Sme2.5_01952.1_g00004.1	60S ribosomal protein L5-like	34877.01	0.118	3	0.492	0.009
Sme2.5_02393.1_g00005.1	zeatin O-xylosyltransferase-like	61266.97	0.011	1	0.532	0.041
Sme2.5_02262.1_g00006.1	uncharacterized protein LOC101250613	31072.98	0.445	9	0.469	0.006
Sme2.5_02324.1_g00008.1	uncharacterized protein At4g01150, chloroplastic-like isoform 2	17951.45	0.124	2	0.648	0.011
Sme2.5_07601.1_g00001.1	methionine sulfoxide reductase A3	35147.93	0.030	1	0.613	0.003
Sme2.5_13307.1_g00002.1	probable inactive purple acid phosphatase 27-like	49929.94	0.499	5	0.564	0.001
Sme2.5_00499.1_g00004.1	glutamate decarboxylase isoform2	60122.57	0.408	14	0.651	0.001
Sme2.5_00776.1_g00002.1	caffeoyl-CoA O-methyltransferase 6-like	27700.24	0.147	2	0.607	0.038
Sme2.5_03911.1_g00003.1	L-ascorbate oxidase homolog	60764.81	0.118	5	0.664	0.007
Sme2.5_00036.1_g00030.1	40S ribosomal protein S19-3-like	16111.41	0.517	1	0.650	0.006
Sme2.5_00014.1_g00037.1	Chain M, Localization Of The Large Subunit Ribosomal Proteins Into A 5.5 A Cryo-Em Map Of Triticum Aestivum Translating 80s Ribosome	15158.07	0.514	6	0.591	0.001
Sme2.5_01731.1_g00001.1	phospholipase A1-II 1-like isoform 1	44138.36	0.756	11	0.603	0.001
Sme2.5_00232.1_g00001.1	Luminal-binding protein 5	73542.99	0.596	9	0.555	0.001

We compared expression differences of L-morph and S-morph pistils between development and maturity. For L-morph flowers, there were 322 upregulated and 322 downregulated genes during maturity compared with development (S2 and S3 Tables). Among upregulated proteins, olee1-like protein-like, fasciclin-like arabinogalactan protein 14-like, profilin-1-like, profilin-1, and cysteine proteinase 3-like, had the highest expression differences (S2 Table). Pectinesterase 2-like was the top differentially upregulated protein. Other proteins, such as nonspecific lipid-transfer protein-like protein At2g13820-like were differentially downregulated (S3 Table). In S-morph flowers, there were 446 upregulated and 550 downregulated genes during maturity compared with development (S4 and S5 Tables). Olee1-like protein-like, nucleoside diphosphate kinase IV, pectinesterase 2-like, profilin-1, and other proteins were the top differentially upregulated proteins (S4 Table). Many downregulated proteins, such as histone H1, ubiquitin extension protein, and cyclic nucleotide-gated ion channel 1-like, had significant expression changes (S5 Table). Pistil extension-like protein was upregulated in L-morph flower pistils from development to maturity but showed no expression difference in S-morph flowers. In S-morph and L-morph flowers, suberization-associated anionic peroxidase 2, which may play an important role in cell wall suberization, superoxide elimination, and oxidative stress response [33,34], was upregulated. We also found that cinnamoyl-CoA reductase, which participates in lignin biosynthesis, was downregulated in both S-morph and L-morph flowers [35].

**Fig 4:**
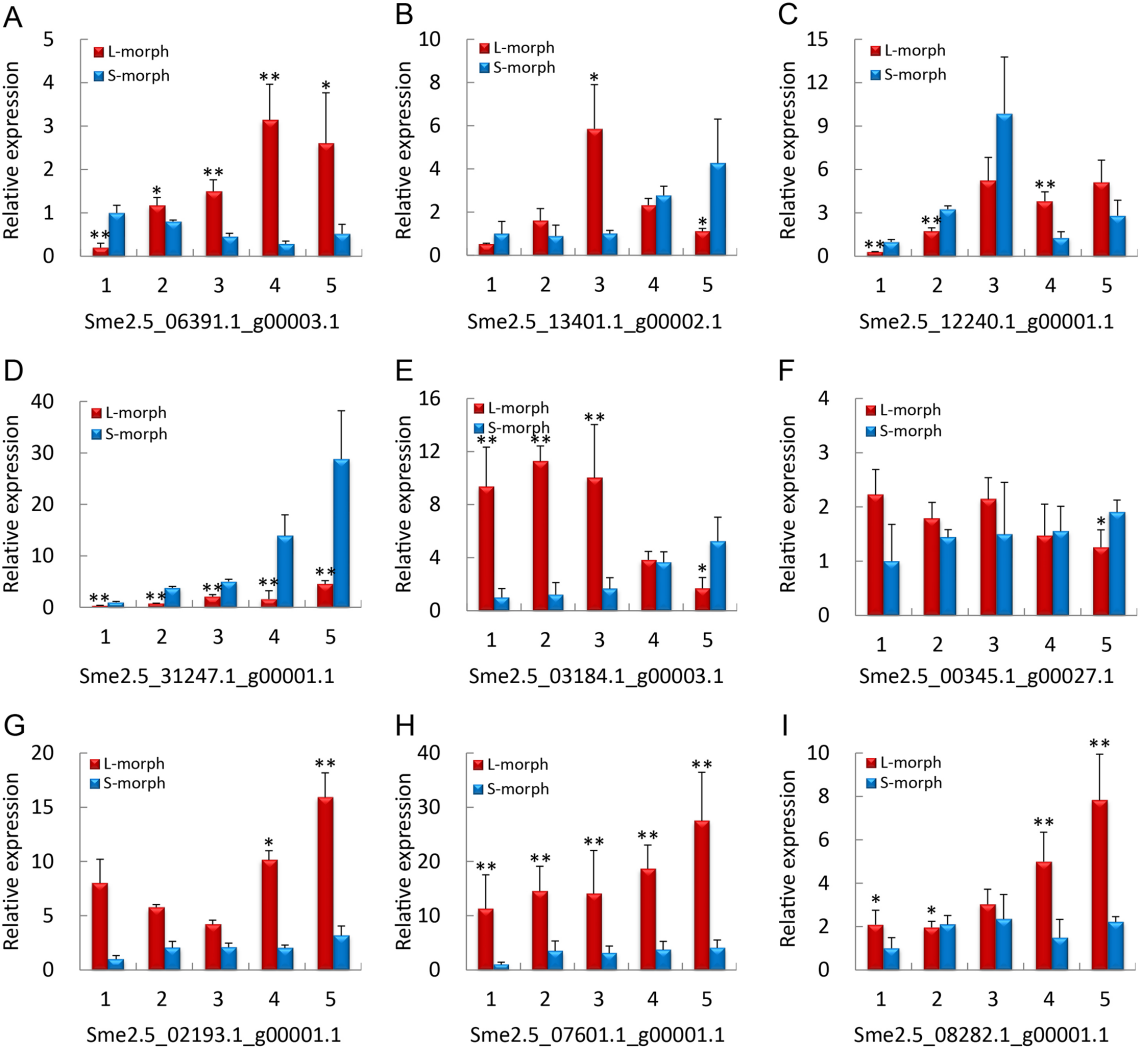
qRT-PCR transcription level of genes related to heterostyly in different stages of S-morph and L-morph flowers. Analysis of -expression of nine genes related to heterostyly in S-morph and L-morph flowers in eggplant by qRT-PCR at 0, 3, 6, 10, or 13 days after budding. Each bar represents the average of three samples ± standard error. Asterisks indicate significant differences (*, *p* < 0.05; **, *p* < 0.01).

To test pistil protein expression levels of messenger RNA (mRNA) transcript differences between L-morph and S-morph flowers and validate the iTRAQ results, we selected nine genes for mRNA qRT-PCR analysis from DAB 0, 3, 6, 10, and 13 of both L-morph and S-morph pistils. As shown in Fig 4, except for Sme2.5_06391.1_g00003.1 and Sme2.5_13401.1_g00002.1, the expression of the other seven genes agreed well with our iTRAQ results from developing and mature flowers, suggesting that most proteins were regulated directly at the transcriptional level. The expression patterns of the two exceptions at the protein and mRNA levels were not consistent with the iTRAQ results during development but were consistent during maturity. This may indicate that the abundance of these proteins depends not only on transcript levels but also on posttranslational modification.

### GO annotation and KEGG pathway analysis of DEPs

**Fig 5:**
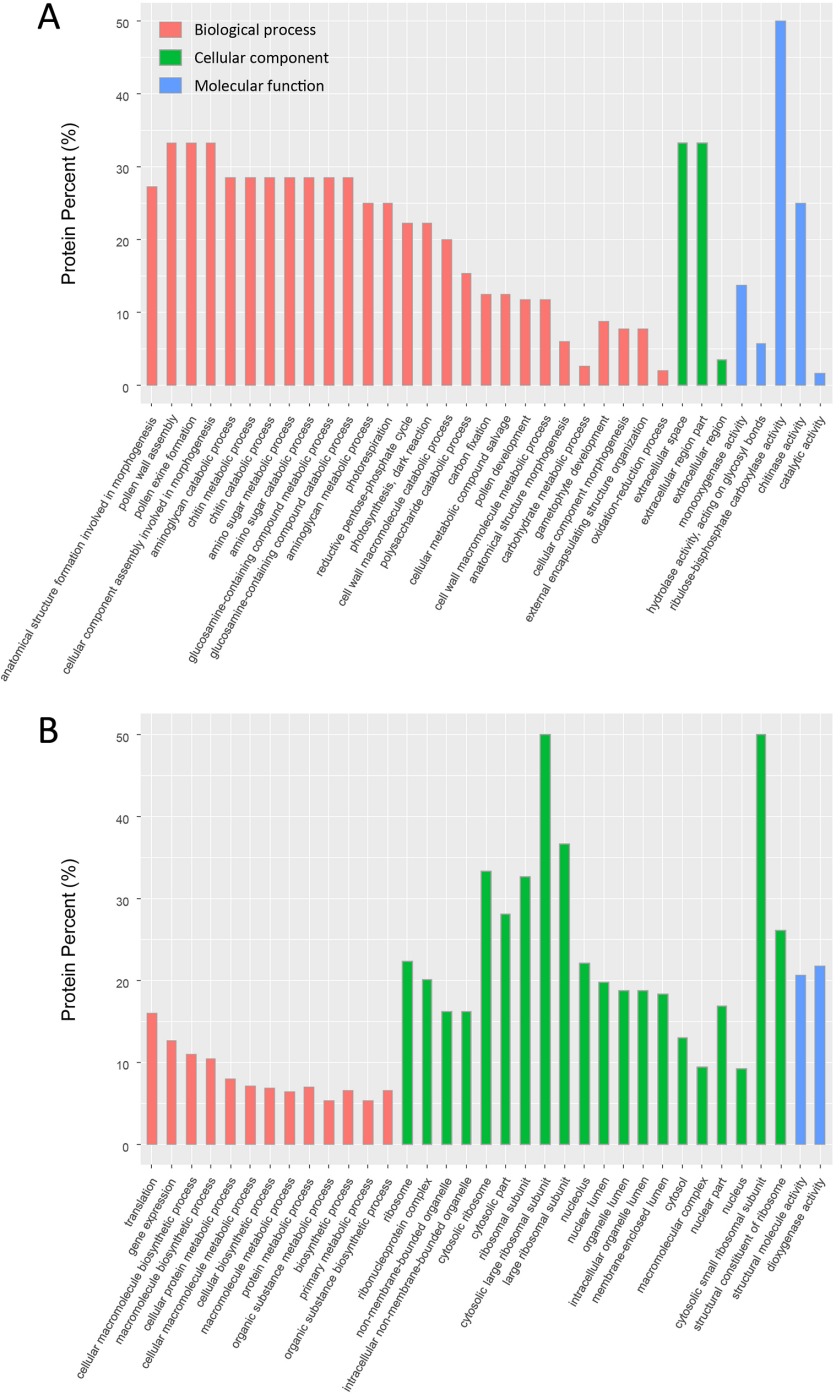
GO annotation of DEPs between L-morph and S-morph flowers at different stages. The distribution of the top 35 enriched GO terms of DEPs during flower development (A) and maturity (B) is shown.

We conducted GO enrichment and KEGG pathway analyses based on a hypergeometric test of all DEPs, with all proteins as the background. As shown in Fig 5, we itemized the respective top significant GO terms in Fig 5 during flower development and maturity. Fig 5A shows that significant biological processes during flower development concerned morphogenesis and metabolic processes. Significant biological processes varied greatly during flower maturity and included translation, gene expression, biosynthetic processes, and metabolic progress (Fig 5B). These findings are in agreement with current understanding of mature plant biochemical and physiological activities. For cellular components, terms related to the ribosome were enriched, suggesting that mature flowers had extensive and abundant translation, transcription, and regulation, more than in the developmental stage.

We also conducted a GO enrichment analysis for DEPs of L-morph and S-morph flowers between developing and mature stages. In the L-morph flowers, significant biological processes included primary metabolic processes and carbohydrate metabolic processes (S4A Fig). However, for the S-morph flowers, the significant enriched biological processes were primarily metabolic processes, translation, and gene expression (S4B Fig).

**Fig 6:**
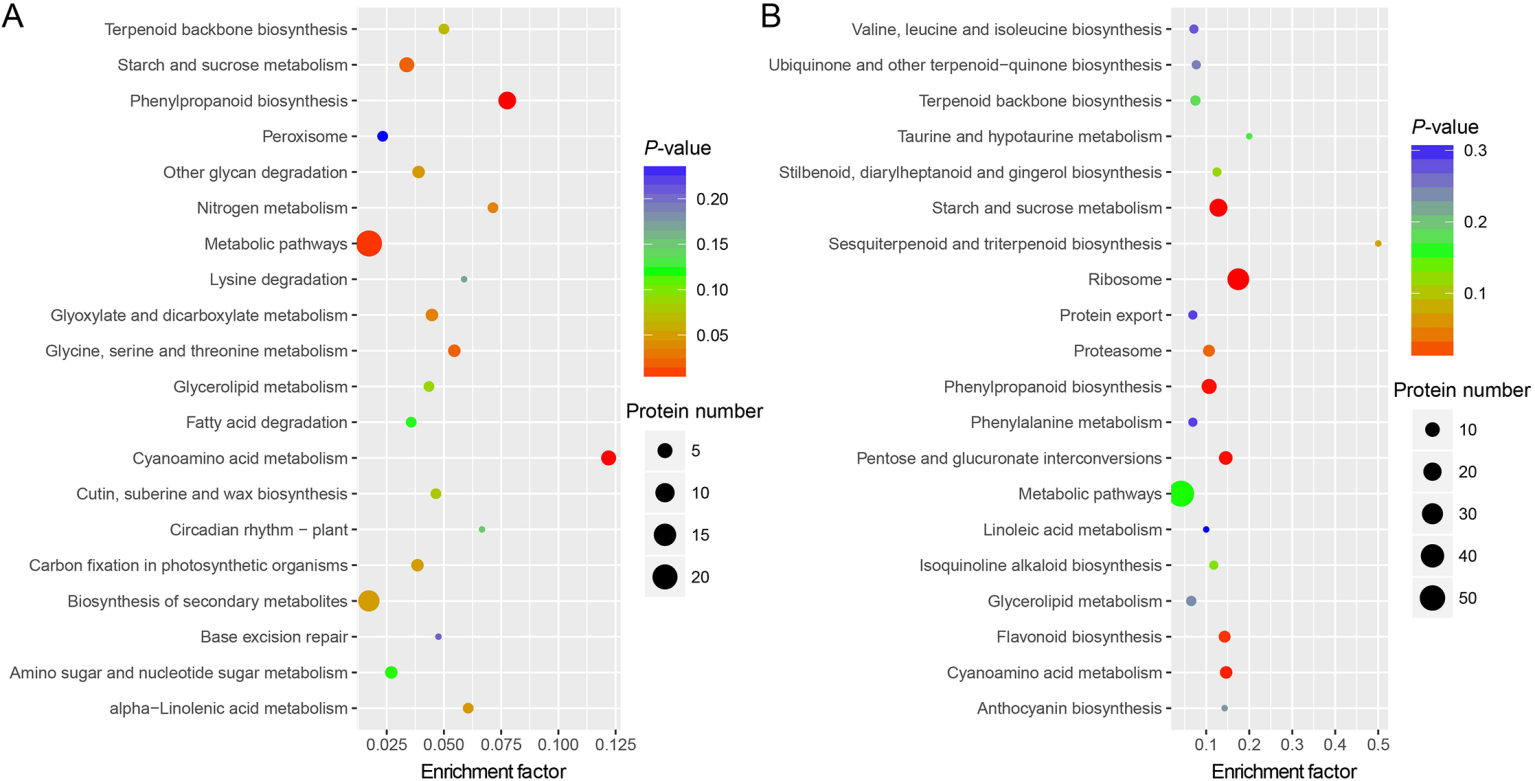
KEGG pathway enrichment of the DEPs at different stages. The distribution of the top 20 enriched KEGG pathways of DEPs during flower development (A) and maturity (B) is shown.

The top 20 significant KEGG pathways are illustrated in Figs 6 and S4. Phenylpropanoid biosynthesis and metabolic pathways were enriched during flower development (Fig 6A). The enriched pathways during flower maturity were ribosome formation, starch and sucrose metabolism, cyanoamino acid metabolism, pentose and glucuronate interconversions, phenylpropanoid biosynthesis, flavonoid biosynthesis, and proteasome formation (Fig 6B). Enriched DEP pathways in L-morph flowers during maturity relative to development included metabolic pathways, molecular biosynthesis, ribosome formation, carbon fixation, and ribonucleic acid (RNA) polymerase biosynthesis (S5A Fig). Enriched DEP pathways in S-morph flowers during maturity relative to development included carbon fixation, ribosome formation, metabolic pathways, proteasome biosynthesis, molecular biosynthesis, and aminoacyl-transfer RNA biosynthesis (S5B Fig).

### Protein-protein interaction analysis

**Fig 7:**
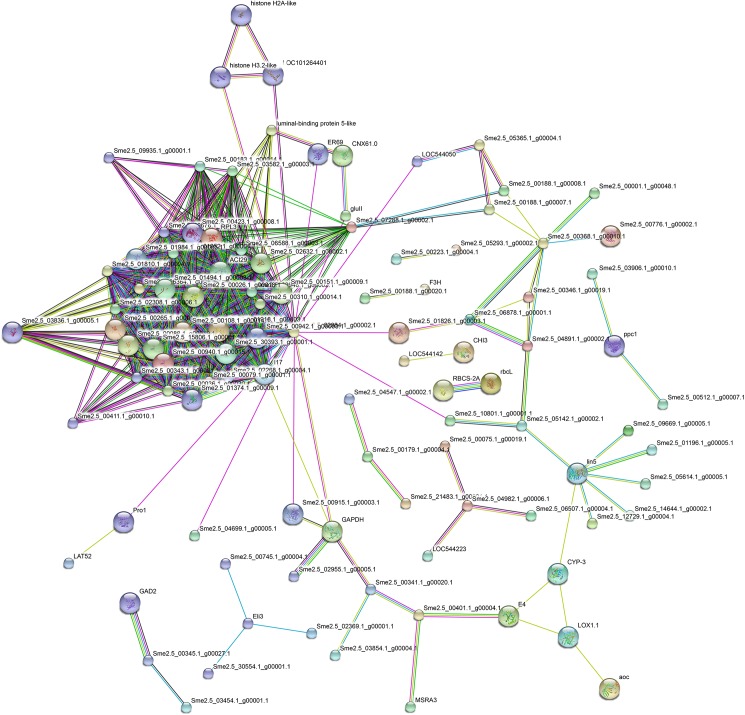
Protein-protein interaction network analyzed by STRING software. Network analysis results for significantly changed proteins between S-morph and L-morph flowers. The confidence score was set to ≥ 0.4 (medium). Different line colors represent the types of evidence for association. Known interactions: magenta = experimental evidence; light blue = database evidence. Predicted interactions: green = neighborhood evidence; red = fusion evidence; blue = co-occurrence evidence. Other: black = coexpression evidence; yellow = text-mining evidence; purple = protein homology.

Proteins in organisms do not act as single entities but rather form a variety of functional connections with each other, and these connections are fundamental in cellular processes. To uncover functional aspects associated with proteins in eggplant flowers, 225 proteins (Tables 1–4, with 16 overlapping DEPs between flower development and maturity) with significantly changed expression were analyzed by searching the STRING database. Fig 7 illustrates that six separate interaction networks were predicted. In each network, proteins that increased interacted with proteins that decreased to constitute a gene regulation network. These proteins were directly or indirectly related to pistil development differences of S-morph and L-morph flowers and were associated primarily with ribosome function, metabolic pathways, phenylpropanoid biosynthesis, starch and sucrose metabolism, and biosynthesis of secondary metabolites (Fig 7). Although these predicted interaction networks still need to be verified and further analyzed in future studies, the interactions between these proteins suggest that they have important roles in heterostylous development in eggplant.

## Discussion

Heterostyly, which produces a distinctive flower polymorphism and results in hercogamy, is widely distributed in angiosperms [1,27,28]. Heterostyly had important implications in plant adaptability and yield because it affects breeding systems. Phylogenetic analysis has shown that this phenotype has originated independently among the heterostylous species and arisen via distinct evolutionary developmental pathways [3]. However, eggplant heterostyly has not been well studied. The study of regulated gene expression levels in producing different flower morphs should give insights into mechanisms involved in heterostyly development. We found that buds of both L- and S-morph lengthened linearly during development. However, around 10 DAB, the L-morph pistils continued to increase but the S-morph pistils generally did not elongate. The self- and cross-pollination of L-morph and S-morph flowers indicated that the structure of the stigmatic surface in S-morph flowers may inhibit pollen germination.

A previous study [20] found that the pollination efficiency of L-morph flowers was the greatest, with a pollen germination percentage up to 100%. However, the percentage of pollen germination on the stigmas of S-morph flowers was much lower (< 5%). Another study found that the viability of pollen from L-morph and S-morph flowers exceeded 98% [36]. A third study indicated that there were differences in physiological and biochemical properties of stigmas between S-morph and L-morph flowers, and potassium levels, which may be influenced by auxin, were lower in S-morph flowers [37]. Rylski *et al*. [38] showed that the stigma of a short style differed from that of a long style in its smaller size, underdeveloped papillae, and lower sugar content. Structural observations of pistils may indicate that the low fertilization rates of S-morph flowers probably resulting from stigmatic incompatibility, rather than defects in the embryo sac. However, it is difficult to determine the mechanism underlying this difference in successful fruit set in S-morph and L-morph flowers.

iTRAQ is an advantageous technology used in comparative proteomic analysis that has provided greater reliability and accuracy for analysis in numerous studies [19,39]. In the present study, we used an iTRAQ approach to investigate the proteomic differences underlying heterostyly in eggplant. We identified 255 DEPs of S-morph and L-morph pistils during floral development and maturity. There were 33 upregulated and 24 downregulated genes during development, and 83 upregulated and 101 downregulated genes during maturity in S-morph compared with L-morph flowers (Tables 1–4). The results of qRT-PCR were highly consistent with our iTRAQ data. We characterized the differentially expressed proteins through GO, KEGG, and protein-protein interaction analyses. The protein expression differences were mainly involved in biosynthesis and metabolism, ribosomes, and expression regulation.

### Proteins involved in biosynthesis and metabolism

During development, molecular biosynthesis and metabolism, including amino acid and fatty acid biosynthesis, are generally upregulated to meet needs for growth. In this proteomic study, 86 proteins involved in biosynthesis and metabolism were differentially expressed between S-morph and L-morph flowers (S6 and S7 Tables), providing evidence that differences in storage and energy availability can play key roles in pistil development. DEPs were involved in the biosynthesis of amino acids including valine, leucine, and isoleucine. Other DEPs were involved in metabolic pathways including glycometabolism, glycerolipid metabolism, and phenylalanine metabolism. Pectin methyl esterase was downregulated in S-morph flowers during maturity; this enzyme is involved in structural modifications of the cell wall during growth and development and is involved in plant-pathogen interactions [40]. Most DEPs (15/19, S7 Table) involved in starch and sucrose metabolism were upregulated in S-morph flowers during maturity. We may infer here that differences in metabolic pathways may play important roles in the development of pistil length. In addition, many proteins were involved in other biosynthetic pathways, such as those for phenylpropanoids, flavonoids, isoquinoline alkaloids, terpenoids, anthocyanins, and other secondary metabolites.

### Proteins involved in ribosomes

The ribosome serves as the site of biological protein synthesis (translation), which links amino acids together in the order specified by messenger RNA molecules. Eukaryotes have 80S ribosomes, each consisting of a small (40S) and large (60S) subunit. In our study, the majority of both 60S and 40S ribosomal proteins was downregulated in S-morph flowers compared with L-morph flowers during maturity (Table 4). There were few differences in expression between the morphs during development (Tables 1 and 2), but the expression of ribosomal proteins was downregulated during maturity in S-morph flowers (Table 4). This may suggest that many proteins need to be translated during maturity as pollination and fruit development proceed.

### Proteins involved in expression regulation

Many binding proteins play important roles in complex and intricate regulatory networks. Here, we identified 14 differentially expressed proteins related to nucleotide binding (RNA-, deoxyribonucleic acid-, and protein-binding factors) between S-morph and L-morph flowers during maturity (Table 2) based on their putative molecular functions; these binding factors might regulate protein activity in complex biological processes. For instance, the expression level of glycine-rich RNA-binding protein (GRP) was upregulated in S-morph flowers during maturity (1.56-fold, Table 3); this protein produces robust circadian oscillations and can suppress expression of the FLOWERING LOCUS C (FLC) protein, thereby promoting flowering [41,42]. FLC expression is directly promoted by histone H2A and leads to delayed flowering during vegetative growth [43]. In S-morph flowers at maturity, the histone H2A expression level was downregulated (1.58-fold, Table 4). Hence, we may infer that GRP and histone H2A tend to downregulate the expression of FLC to promote flowering in S-morph flowers. Additionally, the expression level of profilin-1 tended to be upregulated in S-morph flowers during development and maturity and was also upregulated in L-morph flowers during maturity. Pandey *et al*. [34] demonstrated that constitutive overexpression of cotton profilin-1 in tobacco induced early flowering [44]. Our results suggest that the expression changes of these binding proteins could affect blooming in S-morph flowers. Therefore, proteins involved in RNA-, deoxyribonucleic acid-, or protein-binding may be connected with heterostyly and regulate the pistil length of eggplant flowers.

Our study presents the first proteomic analysis of heterostyly development in eggplant. We identified approximately 225 DEPs in both developing and mature S-morph and L-morph flowers, whose expression levels might be closely related to heterostyly. Differences in flower development stages of the morphs were apparent in morphogenesis and metabolic processes. We discovered that some proteins associated with senescence and programmed cell death were upregulated in S-morph pistils, which may prevent them from developing into fruit. Our results provide valuable information on heterostyly in eggplant, and future studies of other heterostylous species may find similar mechanisms.

## Conclusions

We performed self- and cross-pollination of L-morph and S-morph flowers and compared embryo sac development in eggplant, which suggested that S-morph flowers have stigma incompatibility features that inhibit pollen germination and subsequent fertilization. To explore the molecular mechanisms underlying heterostylous development, we conducted an iTRAQ-based proteomic analysis of eggplant pistils for L-morph and S-morph flowers. There were 225 DEPs in both developing and mature stages of S-morph and L-morph flowers whose expression levels might be closely related to heterostyly. We also conducted qRT-PCR for nine genes to verify DEPs from the iTRAQ data. Differences during flower development between the morphs were primarily observed in proteins related to morphogenesis and metabolic processes. Important biological processes, including translation, gene expression, biosynthetic processes, and metabolic progress, varied greatly during flower maturity. Additionally, we discovered that some proteins associated with programmed cell death were upregulated in S-morph pistils; these may be associated with low fruit set in these flowers. Our research provides important information for understanding eggplant heterostyly and establishes a foundation for the study of relevant mechanisms. It also highlights the importance of complex characters for understanding relationships between proteomic and phenotypic variation.

## Supporting information

S1 Fig General workflow and summary of the present study. The figure shows the workflow from sample collection to iTRAQ as well as the downstream analyses.
(TIF)

S2 Fig Identified proteins. (A) Protein identification coverage distribution. Total spectra = the total number of identified secondary spectra. Spectra = the number of spectra matched. Unique spectra = the number of unique peptide spectra. Peptide = the total number of identified peptides. Unique peptide = the number of identified unique peptides. Protein = the total number of identified proteins. (B) Peptide length distribution. The x-axis shows the peptide length and the y-axis shows the corresponding peptide percentage. (C) Unique peptide number distribution. The x-axis shows the unique peptide number of each protein and the y-axis shows the corresponding protein number.
(TIF)

S3 Fig Function annotation of identified proteins. (A) COG classification of identified proteins. The horizontal axis is the COG function class and the vertical axis is the number of proteins in each class. (B) GO annotation of all identified proteins. C. KEGG pathway analysis of all identified proteins.
(TIF)

S4 Fig GO annotation of DEPs of L-morph and S-morph flowers between different stages. The distribution of the top 35 enriched GO terms of DEPs for L-morph (A) and S-morph flowers (B) between flower development and maturity is shown.
(TIF)

S5 Fig KEGG pathway enrichment of the DEPs during different stages. The distribution of the top 20 enriched KEGG pathways of DEPs for L-morph (A) and S-morph flowers (B) between flower development and maturity is shown.
(TIF)

S1 Table The primers used for qRT-PCR in the experiment. (DOCX)

S2 Table Upregulated proteins in pistils of L-morph flowers during maturity with a 1.5-fold change compared with developmental stage. (DOCX)

S3 Table Downregulated proteins in pistils of L-morph flowers during maturity with a 1.5-fold change compared with developmental stage. (DOCX)

S4 Table Upregulated proteins in pistils of S-morph flowers during maturity with a 1.5-fold change compared with developmental stage. (DOCX)

S5 Table Downregulated proteins in pistils of S-morph flowers during maturity with a 1.5-fold change compared with developmental stage. (DOCX)

S6 Table DEPs between S-morph and L-morph flowers enriched in each pathway during development. (DOCX)

S7 Table DEPs between S-morph and L-morph flowers enriched in each pathway during maturity. (DOCX)
