# Supplementary figures and images for: Comparative proteomic analysis of eggplant (Solanum melongena L.) heterostylous pistil development
Source: PLoS One. 2017 Jun 6;12(6):e0179018. doi: 10.1371/journal.pone.0179018 (PMC5460878; doi:10.1371/journal.pone.0179018)

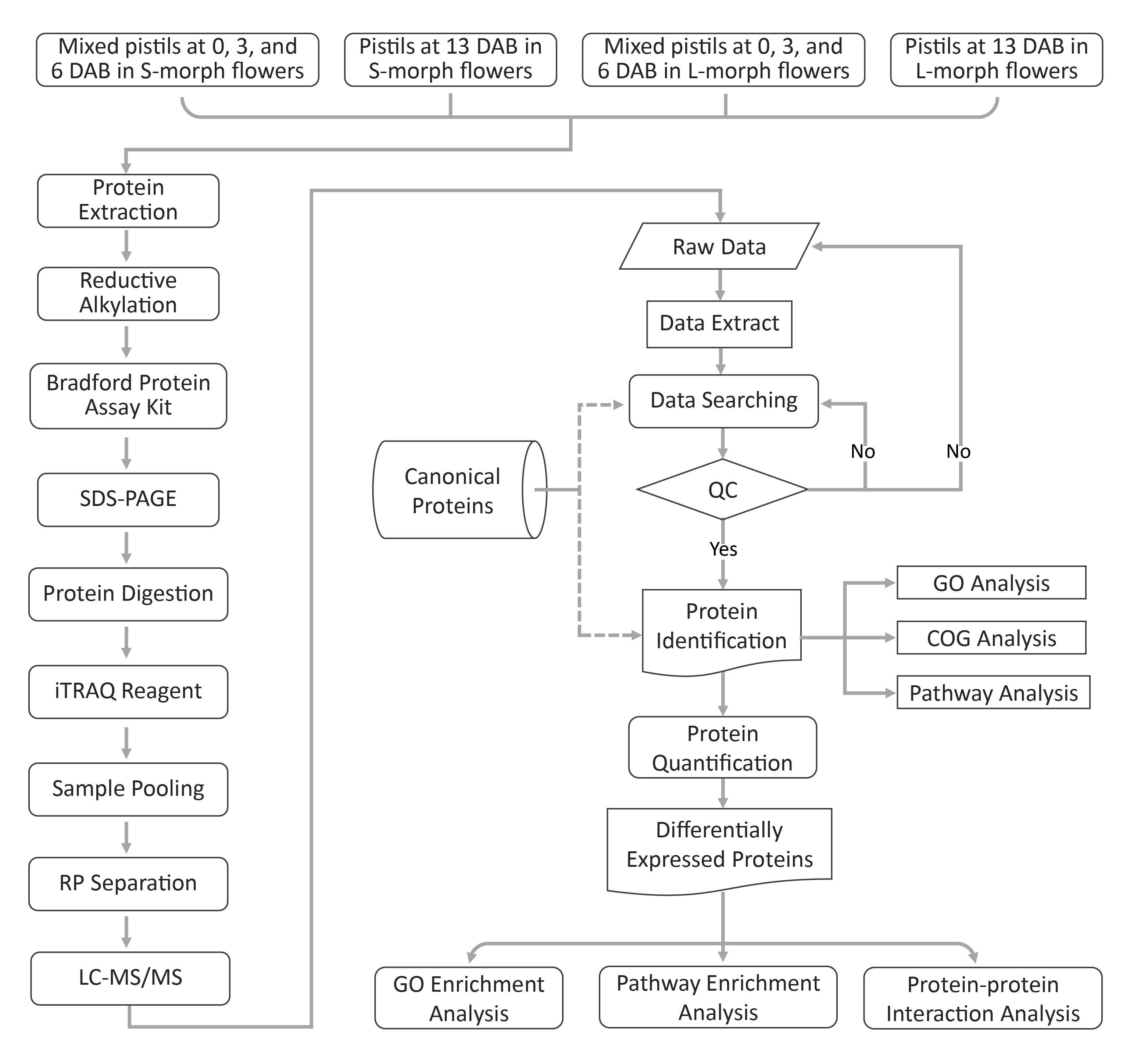

Supplement: S1 Fig — The figure shows the workflow from sample collection to iTRAQ as well as the downstream analyses. (TIF) [file pone.0179018.s001.tif]

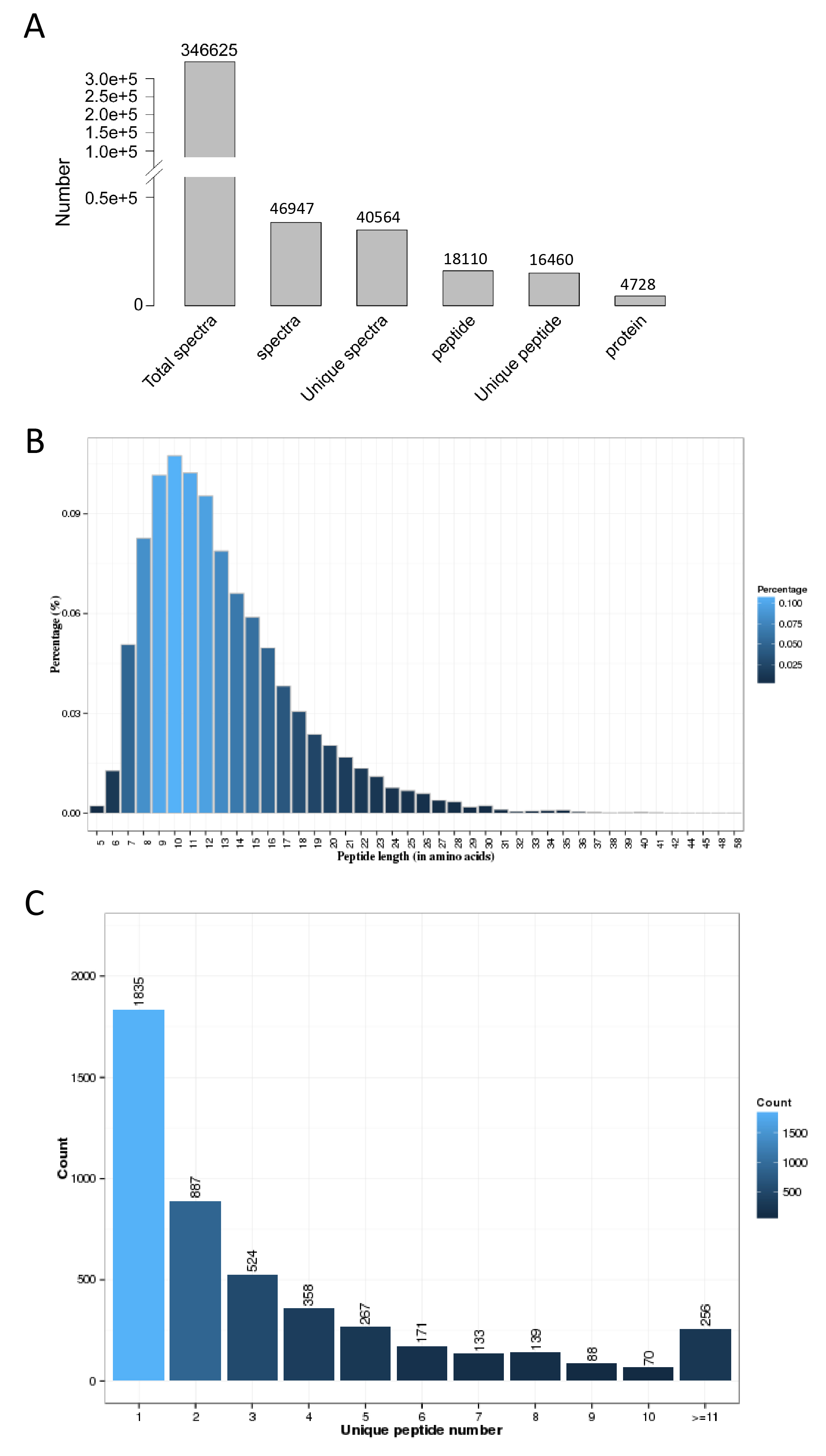

Supplement: S2 Fig — (A) Protein identification coverage distribution. Total spectra = the total number of identified secondary spectra. Spectra = the number of spectra matched. Unique spectra = the number of unique peptide spectra. Peptide = the total number of identified peptides. Unique peptide = the number of identified unique peptides. Protein = the total number of identified proteins. (B) Peptide length distribution. The x-axis shows the peptide length and the y-axis shows the corresponding peptide percentage. (C) Unique peptide number distribution. The x-axis shows the unique peptide number of each protein and the y-axis shows the corresponding protein number. (TIF) [file pone.0179018.s002.tif]

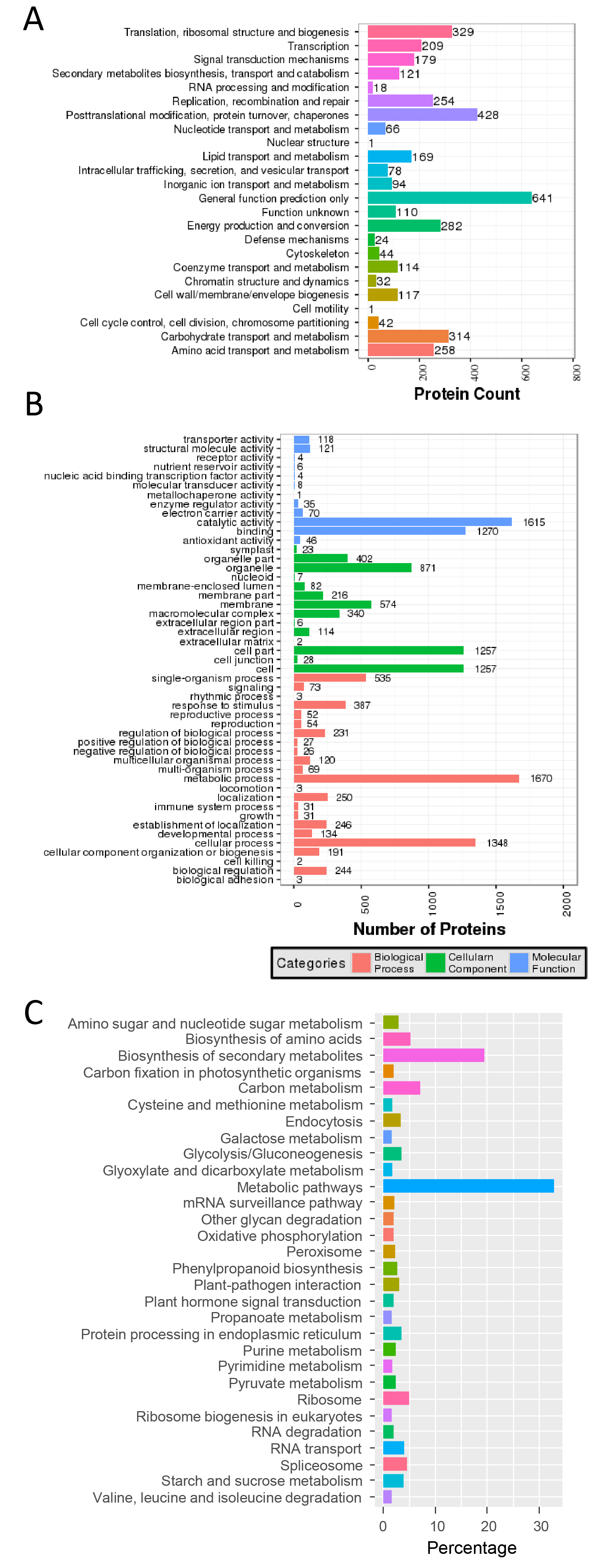

Supplement: S3 Fig — (A) COG classification of identified proteins. The horizontal axis is the COG function class and the vertical axis is the number of proteins in each class. (B) GO annotation of all identified proteins. C. KEGG pathway analysis of all identified proteins. (TIF) [file pone.0179018.s003.tif]

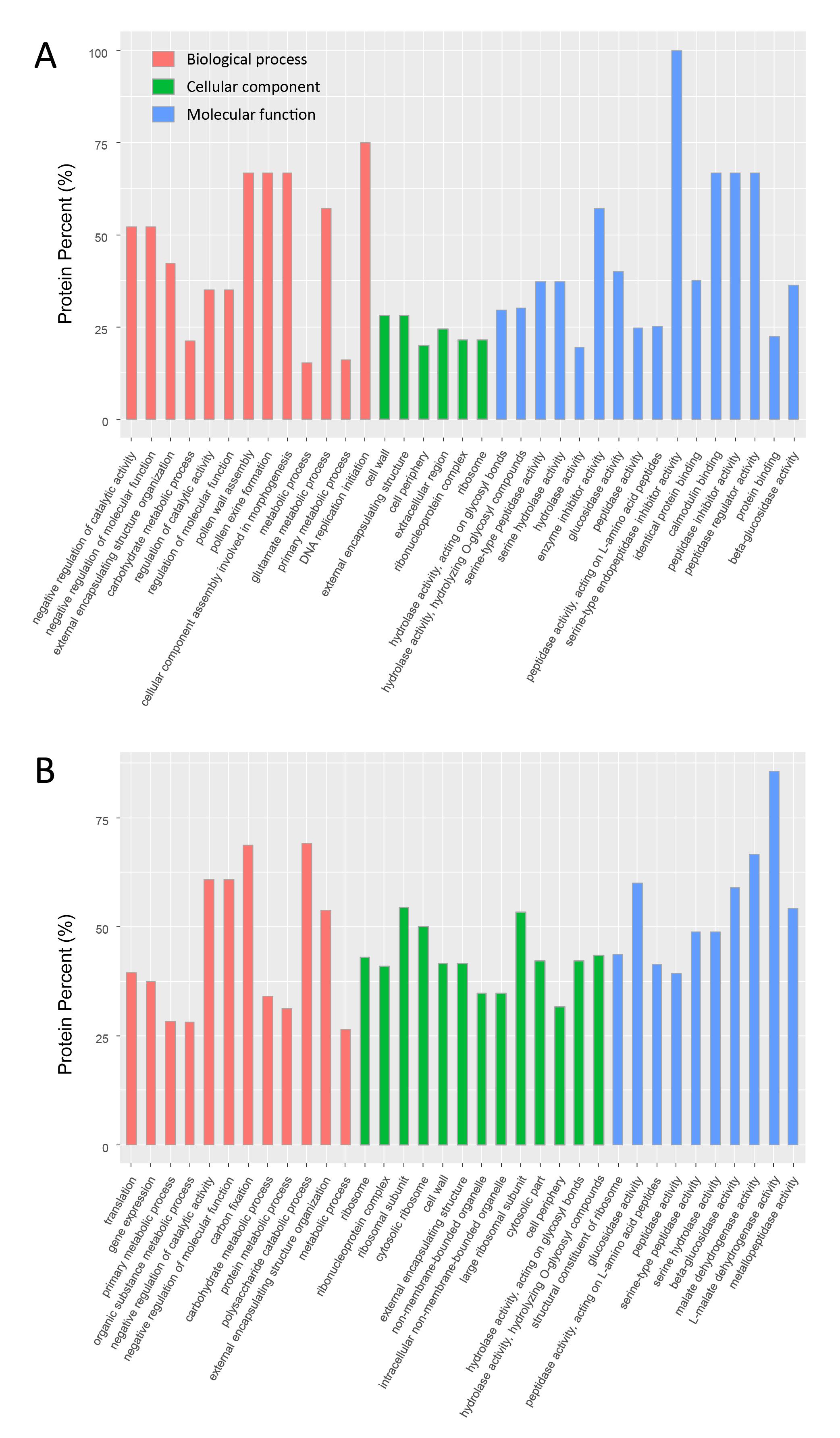

Supplement: S4 Fig — The distribution of the top 35 enriched GO terms of DEPs for L-morph (A) and S-morph flowers (B) between flower development and maturity is shown. (TIF) [file pone.0179018.s004.tif]

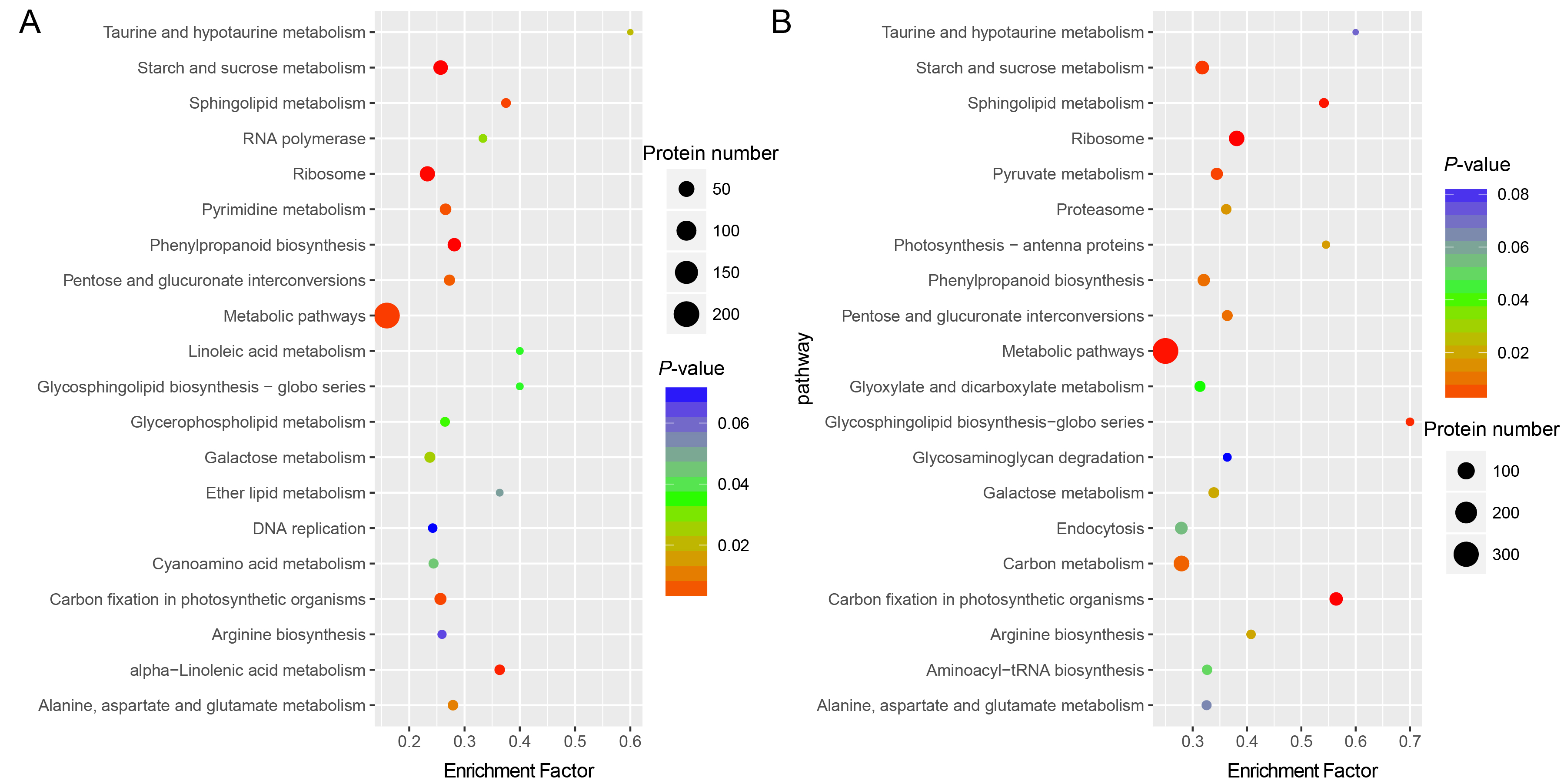

Supplement: S5 Fig — The distribution of the top 20 enriched KEGG pathways of DEPs for L-morph (A) and S-morph flowers (B) between flower development and maturity is shown. (TIF) [file pone.0179018.s005.tif]
