# Supplementary material for: Comparative proteomic analysis of eggplant (Solanum melongena L.) heterostylous pistil development
Source: PLoS One. 2017 Jun 6;12(6):e0179018. doi: 10.1371/journal.pone.0179018 (PMC5460878; doi:10.1371/journal.pone.0179018)
Supplement: S1 Table — (DOCX) [file pone.0179018.s006.docx]

**Table S1. The primers used for qRT-PCR in the experiment.**

| Gene ID | F (5’-3’) | R (5’-3’) |
| --- | --- | --- |
| Sme2.5_06391.1_g00003.1 | GTTTTAGCAAGAGGAGCAA | ATCCGTATGTAAAAGTGCC |
| Sme2.5_13401.1_g00002.1 | CTCACCCCAATCTTTCTCAG | AAGGCTTCACATCCTCACCA |
| Sme2.5_12240.1_g00001.1 | ATGTGAAGGGAAAGTGGAG | AAGGTATAGCTGACGCAAGG |
| Sme2.5_31247.1_g00001.1 | TCCCACATCGGTTGTTCTA | CAATATGCTTCAGCCTTTC |
| Sme2.5_03184.1_g00003.1 | TAGAGTCCGTTCTGATTGC | TCCTCACTTGATGATACGC |
| Sme2.5_00345.1_g00027.1 | GTAGTAGTAGGCGTTGCTGGTG | ACGGTCATAGTCTTCGGTGTCT |
| Sme2.5_08282.1_g00001.1 | TAACAGACTTTCAGGGAGCA | CGATAAATGTGGCAGACGAT |
| Sme2.5_02193.1_g00001.1 | CCAGTGGGCGGAGGACAAGTA | TTAGGTCTCGGCGTCACAAAT |
| Sme2.5_07601.1_g00001.1 | ACAAAGACTGAAGTTGGGTAT | TCAAGATGAAGGAAGCGAGAT |
| GAPDH (internal reference gene) | TCCACTCCATCACAGCCACTCAG | GAACGAAGCAGCTCTTCCACCTC |
