# Supplementary material for: Comparative proteomic analysis of eggplant (Solanum melongena L.) heterostylous pistil development
Source: PLoS One. 2017 Jun 6;12(6):e0179018. doi: 10.1371/journal.pone.0179018 (PMC5460878; doi:10.1371/journal.pone.0179018)
Supplement: S2 Table — (DOCX) [file pone.0179018.s007.docx]

**Table S2 Upregulated proteins in pistils of L-morph flowers during maturity with a 1.5-fold change compared with developmental stage**

| **Protein_ID** | **Description** | **Mass** | **Coverage** | **Peptide** | **Fold change** | **Qvalue** |
| --- | --- | --- | --- | --- | --- | --- |
| Sme2.5_02845.1_g00005.1 | neutral ceramidase-like isoform 1 | 88671.14 | 0.196 | 12 | 1.551 | 0.001 |
| Sme2.5_02083.1_g00006.1 | putative pectinesterase/pectinesterase inhibitor 28-like | 60660.48 | 0.078 | 4 | 1.875 | 0.004 |
| Sme2.5_04861.1_g00004.1 | subtilisin-like protease-like | 83522.91 | 0.163 | 7 | 1.714 | 0.001 |
| Sme2.5_05967.1_g00002.1 | NPL4-like protein 2-like | 44630.82 | 0.113 | 3 | 1.516 | 0.034 |
| Sme2.5_01170.1_g00006.1 | peroxidase 4-like isoform 2 | 16852.54 | 0.099 | 1 | 6.882 | 0.045 |
| Sme2.5_00076.1_g00003.1 | leucine-rich repeat extensin-like protein 3-like | 56883.38 | 0.064 | 3 | 3.526 | 0.031 |
| Sme2.5_08190.1_g00002.1 | probably inactive leucine-rich repeat receptor-like protein kinase At5g48380-like isoform 1 | 24805.47 | 0.204 | 4 | 1.627 | 0.001 |
| Sme2.5_02016.1_g00006.1 | unknown | 25367.63 | 0.036 | 1 | 2.058 | 0.011 |
| Sme2.5_03104.1_g00006.1 | probable pectinesterase/pectinesterase inhibitor 34-like | 62796.54 | 0.085 | 4 | 1.972 | 0.007 |
| Sme2.5_04901.1_g00003.1 | pollen-specific leucine-rich repeat extensin-like protein 1-like | 106873.70 | 0.028 | 2 | 2.423 | 0.032 |
| Sme2.5_03160.1_g00002.1 | uncharacterized protein LOC101252095 | 48968.80 | 0.093 | 3 | 2.583 | 0.001 |
| Sme2.5_03772.1_g00001.1 | vacuolar-processing enzyme | 53905.30 | 0.052 | 2 | 1.850 | 0.014 |
| Sme2.5_15548.1_g00003.1 | germin-like protein subfamily 1 member 14-like | 24586.66 | 0.439 | 5 | 1.510 | 0.001 |
| Sme2.5_08492.1_g00001.1 | inositol monophosphatase 3 | 31156.59 | 0.144 | 3 | 1.830 | 0.015 |
| Sme2.5_00737.1_g00004.1 | 3-ketoacyl-CoA thiolase 2, peroxisomal-like | 49560.29 | 0.411 | 12 | 2.047 | 0.001 |
| Sme2.5_00230.1_g00015.1 | nucleoside diphosphate kinase 1 | 25846.30 | 0.231 | 2 | 1.596 | 0.003 |
| Sme2.5_01542.1_g00007.1 | nuclease S1 | 34412.92 | 0.101 | 3 | 1.540 | 0.001 |
| Sme2.5_16290.1_g00002.1 | unknown | 6759.292 | 0.383 | 2 | 1.685 | 0.033 |
| Sme2.5_02877.1_g00002.1 | uncharacterized protein LOC101268115 | 39131.90 | 0.131 | 5 | 6.952 | 0.001 |
| Sme2.5_22194.1_g00001.1 | kirola-like isoform 1 | 14872.79 | 0.289 | 1 | 2.451 | 0.030 |
| Sme2.5_30554.1_g00001.1 | cell wall peroxidase | 15930.86 | 0.083 | 1 | 6.495 | 0.001 |
| Sme2.5_04037.1_g00003.1 | glutelin type-A 2-like | 28656.76 | 0.342 | 2 | 1.910 | 0.001 |
| Sme2.5_04102.1_g00013.1 | flavoprotein WrbA-like | 20347.23 | 0.505 | 5 | 2.001 | 0.001 |
| Sme2.5_01818.1_g00005.1 | regulator of ribonuclease-like protein 2-like | 18013.34 | 0.307 | 5 | 1.535 | 0.001 |
| Sme2.5_01333.1_g00010.1 | cysteine protease TDI-65 precursor | 52047.96 | 0.209 | 6 | 1.892 | 0.001 |
| Sme2.5_02596.1_g00006.1 | fasciclin-like arabinogalactan protein 9-like | 27164.06 | 0.276 | 6 | 3.215 | 0.001 |
| Sme2.5_02533.1_g00006.1 | olee1-like protein-like | 19924.59 | 0.114 | 2 | 10.000 | 0.043 |
| Sme2.5_01388.1_g00007.1 | beta-hexosaminidase 1 precursor | 67013.25 | 0.066 | 4 | 1.608 | 0.011 |
| Sme2.5_05247.1_g00010.1 | P69F protein | 72319.68 | 0.359 | 15 | 1.582 | 0.001 |
| Sme2.5_05302.1_g00007.1 | uncharacterized protein LOC101260427 | 33787.29 | 0.189 | 1 | 1.662 | 0.036 |
| Sme2.5_12387.1_g00002.1 | miraculin-like | 23795.25 | 0.303 | 5 | 2.385 | 0.001 |
| Sme2.5_05092.1_g00005.1 | fasciclin-like arabinogalactan protein 14-like | 18313.54 | 0.124 | 2 | 10.000 | 0.037 |
| Sme2.5_00276.1_g00015.1 | cytochrome b-c1 complex subunit Rieske, mitochondrial-like | 15039.59 | 0.281 | 3 | 1.699 | 0.028 |
| Sme2.5_00250.1_g00003.1 | subtilisin-like protease-like | 78178.35 | 0.063 | 2 | 2.163 | 0.001 |
| Sme2.5_01674.1_g00011.1 | apoplastic invertase | 56749.38 | 0.275 | 12 | 5.413 | 0.001 |
| Sme2.5_03478.1_g00004.1 | sister chromatid cohesion 1 protein 1-like | 123621.10 | 0.075 | 4 | 2.782 | 0.001 |
| Sme2.5_00944.1_g00019.1 | Blue copper protein precursor, putative | 25228.61 | 0.071 | 1 | 2.170 | 0.001 |
| Sme2.5_01689.1_g00009.1 | LEA1-like protein | 21992.52 | 0.107 | 2 | 1.655 | 0.037 |
| Sme2.5_02832.1_g00003.1 | glucan endo-1,3-beta-glucosidase 6-like | 52803.28 | 0.224 | 7 | 1.694 | 0.001 |
| Sme2.5_04067.1_g00002.1 | NADP-dependent malic enzyme-like | 75539.21 | 0.107 | 5 | 2.001 | 0.001 |
| Sme2.5_04459.1_g00001.1 | probable polygalacturonase-like | 52207.86 | 0.159 | 4 | 2.271 | 0.001 |
| Sme2.5_07722.1_g00003.1 | peptide-N4-(N-acetyl-beta-glucosaminyl)asparagine amidase A-like | 68964.34 | 0.105 | 6 | 1.543 | 0.001 |
| Sme2.5_00021.1_g00030.1 | phosphoserine phosphatase, chloroplastic-like isoform 1 | 32253.51 | 0.224 | 5 | 1.521 | 0.001 |
| Sme2.5_06660.1_g00003.1 | uncharacterized protein LOC101266493 isoform 1 | 20375.65 | 0.173 | 3 | 2.620 | 0.001 |
| Sme2.5_14121.1_g00001.1 | gamma aminobutyrate transaminase 1, mitochondrial | 86072.04 | 0.089 | 5 | 1.583 | 0.012 |
| Sme2.5_04222.1_g00005.1 | aspartic proteinase-like protein 2-like | 36880.22 | 0.069 | 2 | 1.687 | 0.033 |
| Sme2.5_01689.1_g00008.1 | water stress-induced ER5 protein | 17806.15 | 0.282 | 4 | 2.075 | 0.001 |
| Sme2.5_01148.1_g00003.1 | lysosomal beta glucosidase-like | 76951.12 | 0.272 | 13 | 2.154 | 0.001 |
| Sme2.5_03789.1_g00005.1 | serine carboxypeptidase-like 40-like | 56765.17 | 0.234 | 9 | 2.037 | 0.001 |
| Sme2.5_00622.1_g00026.1 | enoyl-[acyl-carrier-protein] reductase [NADH], chloroplastic-like | 41681.60 | 0.459 | 9 | 1.515 | 0.001 |
| Sme2.5_03383.1_g00005.1 | late embryogenesis abundant protein 1-like | 9375.45 | 0.409 | 2 | 4.592 | 0.027 |
| Sme2.5_00098.1_g00017.1 | dihydrolipoyllysine-residue succinyltransferase component of 2-oxoglutarate dehydrogenase complex 2, mitochondrial-like | 48117.14 | 0.131 | 5 | 2.089 | 0.001 |
| Sme2.5_01439.1_g00003.1 | hypothetical protein VITISV_001382 | 279077.90 | 0.043 | 10 | 1.828 | 0.001 |
| Sme2.5_28576.1_g00001.1 | major facilitator superfamily domain-containing protein 5-like | 24057.30 | 0.088 | 2 | 1.688 | 0.021 |
| Sme2.5_01170.1_g00010.1 | alpha-L-fucosidase 1-like | 54851.62 | 0.248 | 9 | 1.759 | 0.001 |
| Sme2.5_06265.1_g00002.1 | UDP-glucose:protein transglucosylase-like protein SlUPTG1 | 41627.73 | 0.387 | 4 | 1.502 | 0.001 |
| Sme2.5_04948.1_g00003.1 | probable ADP-ribosylation factor GTPase-activating protein AGD13-like isoform 1 | 18710.66 | 0.364 | 4 | 1.686 | 0.001 |
| Sme2.5_00204.1_g00008.1 | inositol-tetrakisphosphate 1-kinase 1-like | 37340.65 | 0.078 | 2 | 2.209 | 0.048 |
| Sme2.5_03712.1_g00005.1 | probable pectinesterase/pectinesterase inhibitor 40-like | 62315.90 | 0.121 | 6 | 1.768 | 0.001 |
| Sme2.5_00817.1_g00004.1 | endonuclease 2-like | 32347.73 | 0.283 | 6 | 2.377 | 0.001 |
| Sme2.5_23785.1_g00001.1 | probable inactive purple acid phosphatase 27-like | 48044.26 | 0.428 | 3 | 1.838 | 0.001 |
| Sme2.5_13245.1_g00002.1 | uncharacterized protein LOC101250259 | 21013.99 | 0.103 | 1 | 1.744 | 0.011 |
| Sme2.5_02464.1_g00004.1 | Rab-GDP dissociation inhibitor | 47405.76 | 0.153 | 4 | 5.509 | 0.010 |
| Sme2.5_00771.1_g00005.1 | uncharacterized protein LOC101250239 | 25095.80 | 0.160 | 3 | 2.521 | 0.001 |
| Sme2.5_02047.1_g00012.1 | serine protease inhibitor 1-like | 24337.43 | 0.359 | 2 | 2.029 | 0.001 |
| Sme2.5_00881.1_g00010.1 | peptide-N4-(N-acetyl-beta-glucosaminyl)asparagine amidase A-like | 67435.44 | 0.097 | 5 | 1.776 | 0.001 |
| Sme2.5_13039.1_g00002.1 | uncharacterized protein LOC100254752 | 16761.07 | 0.240 | 3 | 1.75 | 0.027 |
| Sme2.5_05614.1_g00005.1 | lysosomal beta glucosidase-like | 37057.35 | 0.226 | 5 | 2.062 | 0.001 |
| Sme2.5_07680.1_g00001.1 | peroxidase 4-like | 33154.25 | 0.285 | 7 | 3.612 | 0.002 |
| Sme2.5_00558.1_g00009.1 | heparanase-like protein 3-like | 60996.76 | 0.286 | 11 | 1.951 | 0.001 |
| Sme2.5_07124.1_g00003.1 | beta-D-xylosidase 1 precursor | 84376.62 | 0.081 | 4 | 1.654 | 0.016 |
| Sme2.5_01674.1_g00010.1 | apoplastic invertase | 58606.65 | 0.109 | 4 | 3.000 | 0.001 |
| Sme2.5_00423.1_g00005.1 | aspartate aminotransferase, mitochondrial-like | 47519.68 | 0.165 | 5 | 1.736 | 0.001 |
| Sme2.5_00498.1_g00006.1 | peroxisomal fatty acid beta-oxidation multifunctional protein AIM1-like | 84851.89 | 0.240 | 12 | 1.668 | 0.001 |
| Sme2.5_00622.1_g00028.1 | 18 kDa seed maturation protein-like | 14327.04 | 0.147 | 2 | 1.632 | 0.010 |
| Sme2.5_00039.1_g00009.1 | endo-1,4-beta-xylanase A-like | 114878.00 | 0.031 | 3 | 1.729 | 0.026 |
| Sme2.5_00284.1_g00001.1 | uncharacterized protein LOC101265345 | 52330.50 | 0.181 | 8 | 1.892 | 0.001 |
| Sme2.5_02956.1_g00008.1 | plastid lipid associated protein CHRC | 35277.50 | 0.245 | 8 | 1.665 | 0.001 |
| Sme2.5_07880.1_g00001.1 | glucan endo-1,3-beta-glucosidase 8-like | 85413.96 | 0.037 | 3 | 2.728 | 0.001 |
| Sme2.5_03146.1_g00004.1 | protein ASPARTIC PROTEASE IN GUARD CELL 1-like | 48528.85 | 0.258 | 6 | 1.674 | 0.001 |
| Sme2.5_00514.1_g00004.1 | uncharacterized protein At5g39865-like | 56190.35 | 0.018 | 1 | 1.705 | 0.001 |
| Sme2.5_00003.1_g00041.1 | cysteine proteinase precursor | 32165.38 | 0.306 | 6 | 1.518 | 0.001 |
| Sme2.5_23355.1_g00001.1 | Probable linoleate 9S-lipoxygenase 4 | 79251.74 | 0.717 | 30 | 2.228 | 0.001 |
| Sme2.5_07370.1_g00002.1 | mitochondrial processing peptidase | 59406.29 | 0.425 | 15 | 1.791 | 0.001 |
| Sme2.5_00556.1_g00020.1 | uncharacterized protein LOC101244100 | 43782.57 | 0.118 | 4 | 1.590 | 0.001 |
| Sme2.5_02846.1_g00004.1 | probable phospholipase D F09G2.8-like | 53905.97 | 0.074 | 3 | 1.752 | 0.023 |
| Sme2.5_04637.1_g00002.1 | Polyprotein, putative | 40073.04 | 0.030 | 1 | 9.799 | 0.032 |
| Sme2.5_05247.1_g00007.1 | subtilisin-like protease precursor | 76308.27 | 0.227 | 7 | 1.549 | 0.001 |
| Sme2.5_04397.1_g00006.1 | lysosomal beta glucosidase-like isoform 1 | 68785.35 | 0.197 | 9 | 1.702 | 0.003 |
| Sme2.5_03704.1_g00001.1 | endoglucanase 25-like | 67199.73 | 0.103 | 3 | 1.634 | 0.017 |
| Sme2.5_04699.1_g00005.1 | profilin-1-like | 17863.69 | 0.390 | 4 | 10.000 | 0.001 |
| Sme2.5_06227.1_g00005.1 | late embryogenesis abundant protein D-34-like | 22191.38 | 0.611 | 10 | 3.485 | 0.001 |
| Sme2.5_00225.1_g00028.1 | peroxidase | 34892.72 | 0.322 | 8 | 1.956 | 0.001 |
| Sme2.5_04990.1_g00001.1 | polygalacturonase At1g48100-like | 55302.28 | 0.048 | 2 | 1.696 | 0.001 |
| Sme2.5_03277.1_g00005.1 | L-ascorbate oxidase homolog | 60720.49 | 0.180 | 6 | 2.828 | 0.001 |
| Sme2.5_00001.1_g00040.1 | predicted protein | 8308.48 | 0.264 | 2 | 1.957 | 0.008 |
| Sme2.5_01750.1_g00004.1 | ABC transporter A family member 7-like | 135272.30 | 0.040 | 4 | 1.705 | 0.001 |
| Sme2.5_02784.1_g00004.1 | unknown | 11296.75 | 0.118 | 1 | 6.578 | 0.047 |
| Sme2.5_07915.1_g00002.1 | subtilisin-like protease-like | 81822.36 | 0.276 | 11 | 1.978 | 0.001 |
| Sme2.5_00133.1_g00007.1 | probable inactive purple acid phosphatase 29-like | 42031.89 | 0.232 | 4 | 1.713 | 0.027 |
| Sme2.5_28787.1_g00001.1 | serpin-ZX-like | 11643.97 | 0.734 | 2 | 1.781 | 0.007 |
| Sme2.5_14598.1_g00001.1 | cathepsin B-like | 82575.93 | 0.106 | 4 | 1.934 | 0.001 |
| Sme2.5_00211.1_g00005.1 | aspartic proteinase PCS1-like | 40341.31 | 0.070 | 2 | 5.319 | 0.042 |
| Sme2.5_05015.1_g00003.1 | beta-1,3-glucanase 23 | 51049.72 | 0.104 | 4 | 2.618 | 0.010 |
| Sme2.5_00161.1_g00026.1 | lamin-like protein-like | 19925.09 | 0.166 | 3 | 2.072 | 0.001 |
| Sme2.5_00235.1_g00003.1 | ribonuclease 3-like | 24947.69 | 0.156 | 4 | 2.045 | 0.001 |
| Sme2.5_00162.1_g00022.1 | Threonine dehydratase biosynthetic, chloroplastic | 53592.73 | 0.282 | 3 | 1.915 | 0.001 |
| Sme2.5_01186.1_g00004.1 | subtilisin-like protease-like | 101281.40 | 0.087 | 4 | 2.189 | 0.004 |
| Sme2.5_03899.1_g00003.1 | putative mitochondrial NAD-dependent malate dehydrogenase | 43909.83 | 0.588 | 7 | 1.533 | 0.001 |
| Sme2.5_01731.1_g00003.1 | phospholipase A1-IIgamma-like | 42657.86 | 0.623 | 12 | 1.728 | 0.001 |
| Sme2.5_10294.1_g00002.1 | endochitinase precursor (EC 3.2.1.14), partial | 21750.17 | 0.124 | 2 | 2.592 | 0.001 |
| Sme2.5_02263.1_g00001.1 | pentatricopeptide repeat-containing protein At1g62260, mitochondrial-like | 120403.30 | 0.008 | 1 | 1.588 | 0.047 |
| Sme2.5_05030.1_g00007.1 | protein notum homolog isoform 1 | 44176.03 | 0.298 | 8 | 3.314 | 0.001 |
| Sme2.5_01719.1_g00004.1 | probable methyltransferase PMT26-like | 175592.80 | 0.136 | 9 | 1.861 | 0.001 |
| Sme2.5_03695.1_g00003.1 | d-3-phosphoglycerate dehydrogenase, chloroplastic-like | 60247.72 | 0.074 | 2 | 2.240 | 0.007 |
| Sme2.5_01333.1_g00015.1 | subtilisin-like protease-like | 110426.90 | 0.122 | 7 | 1.726 | 0.001 |
| Sme2.5_02955.1_g00005.1 | unknown | 38980.04 | 0.623 | 9 | 1.634 | 0.001 |
| Sme2.5_02047.1_g00009.1 | miraculin-like, partial | 23253.65 | 0.103 | 2 | 2.114 | 0.001 |
| Sme2.5_01369.1_g00005.1 | ASR4 protein | 32352.42 | 0.323 | 6 | 1.660 | 0.001 |
| Sme2.5_00265.1_g00010.1 | 40S ribosomal protein S25-2-like | 11914.60 | 0.361 | 4 | 1.579 | 0.003 |
| Sme2.5_00817.1_g00007.1 | alpha-glucosidase | 105507.90 | 0.306 | 18 | 1.727 | 0.001 |
| Sme2.5_10801.1_g00002.1 | glutamate decarboxylase-like | 53681.37 | 0.129 | 2 | 3.305 | 0.015 |
| Sme2.5_01223.1_g00005.1 | geranylgeranyl pyrophosphate synthase 1 | 40464.48 | 0.101 | 2 | 2.425 | 0.032 |
| Sme2.5_01747.1_g00001.1 | farnesylcysteine lyase-like | 54803.23 | 0.119 | 5 | 1.575 | 0.001 |
| Sme2.5_00827.1_g00004.1 | uncharacterized protein At4g13230-like | 13245.75 | 0.107 | 1 | 2.711 | 0.021 |
| Sme2.5_00183.1_g00017.1 | pyruvate decarboxylase isozyme 2-like | 65173.42 | 0.088 | 4 | 2.566 | 0.006 |
| Sme2.5_11763.1_g00001.1 | beta-galactosidase 15-like | 92784.96 | 0.073 | 5 | 2.602 | 0.001 |
| Sme2.5_01764.1_g00007.1 | GDSL esterase/lipase APG-like | 39003.76 | 0.312 | 7 | 1.602 | 0.001 |
| Sme2.5_05758.1_g00003.1 | ADP,ATP carrier protein, mitochondrial-like | 40659.79 | 0.168 | 3 | 8.857 | 0.001 |
| Sme2.5_01657.1_g00005.1 | acidic endochitinase-like | 32651.76 | 0.184 | 3 | 2.097 | 0.001 |
| Sme2.5_03720.1_g00003.1 | xyloglucan specific endoglucanase inhibitor | 46164.17 | 0.125 | 4 | 4.992 | 0.001 |
| Sme2.5_14644.1_g00002.1 | predicted protein | 12132.46 | 0.339 | 3 | 2.794 | 0.001 |
| Sme2.5_04699.1_g00002.1 | hexokinase | 53841.67 | 0.329 | 10 | 1.932 | 0.002 |
| Sme2.5_00528.1_g00025.1 | universal stress protein MJ0531-like | 17740.88 | 0.170 | 2 | 2.238 | 0.044 |
| Sme2.5_14501.1_g00004.1 | uncharacterized protein At5g39570-like | 26376.08 | 0.096 | 2 | 2.534 | 0.014 |
| Sme2.5_02047.1_g00011.1 | Kunitz-type protease inhibitor precursor | 26448.45 | 0.280 | 4 | 1.976 | 0.002 |
| Sme2.5_00798.1_g00008.1 | peptidyl-prolyl cis-trans isomerase CYP19-3-like | 19123.56 | 0.309 | 5 | 1.552 | 0.001 |
| Sme2.5_04801.1_g00006.1 | chloroplast lipocalin | 49841.77 | 0.159 | 7 | 1.702 | 0.001 |
| Sme2.5_00661.1_g00010.1 | probable inactive purple acid phosphatase 27-like | 69337.55 | 0.255 | 9 | 1.505 | 0.001 |
| Sme2.5_00563.1_g00021.1 | subtilisin-like protease-like | 78897.38 | 0.210 | 12 | 1.763 | 0.001 |
| Sme2.5_08282.1_g00001.1 | DNA-damage-repair/toleration protein DRT100-like | 40864.38 | 0.198 | 6 | 2.456 | 0.001 |
| Sme2.5_00081.1_g00012.1 | galacturonosyltransferase 8-like | 65131.45 | 0.057 | 2 | 1.666 | 0.035 |
| Sme2.5_03398.1_g00005.1 | protein usf-like | 34756.58 | 0.351 | 8 | 1.582 | 0.001 |
| Sme2.5_09773.1_g00003.1 | alanine aminotransferase 2, mitochondrial-like | 60125.34 | 0.229 | 7 | 1.760 | 0.001 |
| Sme2.5_17566.1_g00001.1 | serine protease inhibitor 1-like | 22109.17 | 0.213 | 2 | 2.770 | 0.001 |
| Sme2.5_00169.1_g00003.1 | NADH-glutamate dehydrogenase | 44787.91 | 0.347 | 10 | 3.158 | 0.001 |
| Sme2.5_01822.1_g00002.1 | linoleate 9S-lipoxygenase B-like | 147390.40 | 0.060 | 2 | 1.802 | 0.007 |
| Sme2.5_00499.1_g00014.1 | dehydrogenase/reductase SDR family member 4-like isoform 1 | 26937.35 | 0.095 | 2 | 1.634 | 0.010 |
| Sme2.5_00162.1_g00020.1 | threonine deaminase, partial | 40661.78 | 0.445 | 3 | 1.826 | 0.003 |
| Sme2.5_29983.1_g00001.1 | xylose isomerase-like | 20389.09 | 0.324 | 5 | 1.550 | 0.001 |
| Sme2.5_04788.1_g00003.1 | N-carbamoyl-L-amino acid hydrolase-like | 54298.54 | 0.311 | 11 | 1.508 | 0.001 |
| Sme2.5_00044.1_g00019.1 | serine carboxypeptidase-like 27-like | 51460.39 | 0.138 | 5 | 1.675 | 0.001 |
| Sme2.5_01130.1_g00002.1 | probable LRR receptor-like serine/threonine-protein kinase At1g14390-like | 81676.77 | 0.075 | 4 | 2.203 | 0.001 |
| Sme2.5_00299.1_g00017.1 | xylem serine proteinase 1-like | 80846.63 | 0.089 | 5 | 3.607 | 0.001 |
| Sme2.5_00254.1_g00008.1 | L-ascorbate oxidase homolog | 61984.82 | 0.159 | 5 | 3.110 | 0.033 |
| Sme2.5_05807.1_g00004.1 | protein ASPARTIC PROTEASE IN GUARD CELL 2-like | 48127.42 | 0.121 | 5 | 3.771 | 0.001 |
| Sme2.5_13225.1_g00001.1 | DNA polymerase alpha catalytic subunit | 140215.50 | 0.072 | 6 | 2.056 | 0.001 |
| Sme2.5_16730.1_g00001.1 | neutral leucine aminopeptidase preprotein | 9523.93 | 0.161 | 1 | 5.671 | 0.001 |
| Sme2.5_02909.1_g00005.1 | serine carboxypeptidase-like 42-like | 52758.22 | 0.373 | 12 | 1.684 | 0.001 |
| Sme2.5_06010.1_g00002.1 | 21 kDa protein-like | 20884.77 | 0.225 | 3 | 1.561 | 0.003 |
| Sme2.5_04600.1_g00004.1 | vacuolar invertase | 99950.54 | 0.174 | 11 | 1.733 | 0.001 |
| Sme2.5_09773.1_g00001.1 | pectin methyl esterase | 63762.58 | 0.261 | 11 | 2.207 | 0.001 |
| Sme2.5_03399.1_g00004.1 | uncharacterized protein LOC101256838 | 26309.87 | 0.038 | 1 | 3.150 | 0.010 |
| Sme2.5_00544.1_g00006.1 | aspartic proteinase nepenthesin-1-like | 47122.38 | 0.151 | 3 | 2.187 | 0.001 |
| Sme2.5_05609.1_g00005.1 | plasma membrane-associated cation-binding protein 1-like isoform 1 | 23052.88 | 0.414 | 7 | 1.574 | 0.001 |
| Sme2.5_01683.1_g00005.1 | alcohol dehydrogenase 1-like | 45116.55 | 0.356 | 9 | 2.630 | 0.001 |
| Sme2.5_00545.1_g00008.1 | aspartic proteinase-like protein 2-like | 97822.40 | 0.075 | 5 | 1.508 | 0.002 |
| Sme2.5_02193.1_g00001.1 | cysteine protease inhibitor 8-like | 34677.99 | 0.144 | 3 | 3.865 | 0.001 |
| Sme2.5_00395.1_g00014.1 | uncharacterized protein LOC101245945 isoform 2 | 70726.16 | 0.086 | 5 | 1.593 | 0.001 |
| Sme2.5_02111.1_g00003.1 | subtilisin-like protease-like | 75293.36 | 0.406 | 15 | 2.125 | 0.001 |
| Sme2.5_00158.1_g00011.1 | phospholipase C 3-like | 59589.84 | 0.052 | 3 | 2.371 | 0.049 |
| Sme2.5_13449.1_g00001.1 | leucine-rich repeat extensin-like protein 4-like | 61751.69 | 0.084 | 3 | 1.814 | 0.017 |
| Sme2.5_00864.1_g00008.1 | oryzain alpha chain-like | 20763.01 | 0.364 | 5 | 4.436 | 0.001 |
| Sme2.5_05247.1_g00009.1 | subtilisin-like protease precursor | 71683.00 | 0.127 | 4 | 2.108 | 0.002 |
| Sme2.5_08781.1_g00001.1 | uncharacterized protein LOC101261020 isoform 2 | 34444.47 | 0.249 | 3 | 1.882 | 0.012 |
| Sme2.5_08304.1_g00001.1 | profilin-1 | 14120.02 | 0.546 | 5 | 10.000 | 0.001 |
| Sme2.5_00414.1_g00005.1 | probable inactive purple acid phosphatase 1-like | 69222.91 | 0.308 | 12 | 1.606 | 0.001 |
| Sme2.5_00381.1_g00003.1 | beta-galactosidase STBG5 | 95767.33 | 0.183 | 11 | 1.857 | 0.001 |
| Sme2.5_01564.1_g00006.1 | subtilisin-like protease-like | 82387.07 | 0.140 | 7 | 1.769 | 0.001 |
| Sme2.5_00294.1_g00012.1 | expansin A4 precursor | 28761.19 | 0.235 | 4 | 1.696 | 0.013 |
| Sme2.5_02975.1_g00002.1 | glutaredoxin | 11338.89 | 0.778 | 6 | 1.699 | 0.001 |
| Sme2.5_12729.1_g00004.1 | lysosomal beta glucosidase-like | 66678.06 | 0.121 | 5 | 1.528 | 0.001 |
| Sme2.5_01937.1_g00005.1 | uncharacterized protein LOC101247575 | 80431.09 | 0.334 | 18 | 2.230 | 0.001 |
| Sme2.5_00019.1_g00029.1 | vicilin-like antimicrobial peptides 2-2-like | 36523.35 | 0.143 | 4 | 10.000 | 0.001 |
| Sme2.5_00086.1_g00011.1 | SlArf/Xyl3 | 84328.53 | 0.245 | 12 | 1.796 | 0.001 |
| Sme2.5_00410.1_g00015.1 | probable pectinesterase/pectinesterase inhibitor 12-like | 75761.67 | 0.113 | 5 | 3.818 | 0.001 |
| Sme2.5_00555.1_g00008.1 | putative mitochondrial NAD-dependent malate dehydrogenase | 36531.25 | 0.711 | 8 | 1.801 | 0.001 |
| Sme2.5_02364.1_g00005.1 | MOSC domain-containing protein 2, mitochondrial-like | 34367.48 | 0.058 | 2 | 1.716 | 0.010 |
| Sme2.5_00086.1_g00013.1 | SlArf/Xyl4 | 83951.83 | 0.250 | 14 | 2.105 | 0.001 |
| Sme2.5_02785.1_g00004.1 | uncharacterized protein LOC101261175 | 45287.48 | 0.027 | 1 | 3.431 | 0.042 |
| Sme2.5_01379.1_g00015.1 | 9-divinyl ether synthase | 53919.86 | 0.040 | 2 | 2.389 | 0.001 |
| Sme2.5_26907.1_g00001.1 | acid phosphatase 1-like | 29750.11 | 0.664 | 3 | 1.548 | 0.001 |
| Sme2.5_06721.1_g00006.1 | aldo-keto reductase family 4 member C10-like | 35532.44 | 0.304 | 8 | 1.772 | 0.001 |
| Sme2.5_11580.1_g00002.1 | thioredoxin H-type 2 | 13068.71 | 0.381 | 3 | 1.631 | 0.041 |
| Sme2.5_05168.1_g00005.1 | subtilisin-like protease-like | 82148.15 | 0.242 | 12 | 1.794 | 0.001 |
| Sme2.5_09889.1_g00002.1 | glucan endo-1,3-beta-glucosidase 7-like | 48258.69 | 0.438 | 12 | 1.727 | 0.001 |
| Sme2.5_07574.1_g00004.1 | malate dehydrogenase, glyoxysomal-like isoform 1 | 37922.76 | 0.283 | 4 | 1.797 | 0.005 |
| Sme2.5_00172.1_g00019.1 | probable polyamine oxidase 4-like | 163629.60 | 0.052 | 6 | 2.113 | 0.001 |
| Sme2.5_00038.1_g00015.1 | cysteine proteinase RD21a-like | 57772.58 | 0.149 | 5 | 1.562 | 0.024 |
| Sme2.5_00768.1_g00018.1 | cysteine proteinase 3-like | 40377.92 | 0.253 | 8 | 10.000 | 0.001 |
| Sme2.5_02852.1_g00011.1 | brassinosteroid LRR receptor kinase-like | 41753.72 | 0.120 | 3 | 1.626 | 0.006 |
| Sme2.5_00539.1_g00002.1 | cytochrome c | 14747.50 | 0.170 | 2 | 2.932 | 0.001 |
| Sme2.5_08981.1_g00001.1 | RNase Phy3, partial | 56609.13 | 0.067 | 3 | 4.834 | 0.009 |
| Sme2.5_00740.1_g00010.1 | denticleless protein homolog A-like | 167057.90 | 0.042 | 6 | 2.251 | 0.012 |
| Sme2.5_08226.1_g00002.1 | glycine-rich RNA-binding protein-like | 15970.37 | 0.362 | 3 | 2.875 | 0.001 |
| Sme2.5_09844.1_g00002.1 | beta-1,3-glucanase 22 | 39677.05 | 0.391 | 10 | 1.770 | 0.001 |
| Sme2.5_00132.1_g00007.1 | protein ASPARTIC PROTEASE IN GUARD CELL 1-like | 52080.77 | 0.148 | 5 | 2.920 | 0.001 |
| Sme2.5_06530.1_g00007.1 | secologanin synthase-like | 88653.63 | 0.088 | 5 | 2.086 | 0.001 |
| Sme2.5_01031.1_g00012.1 | expansin-like protein precursor | 28955.26 | 0.130 | 4 | 2.828 | 0.003 |
| Sme2.5_00225.1_g00038.1 | somatic embryogenesis receptor kinase 3B precursor | 64125.90 | 0.040 | 2 | 1.670 | 0.034 |
| Sme2.5_06196.1_g00002.1 | pentatricopeptide repeat-containing protein At2g02980-like | 82179.71 | 0.033 | 2 | 2.468 | 0.003 |
| Sme2.5_11468.1_g00006.1 | pectin methyl esterase | 61016.08 | 0.435 | 5 | 2.057 | 0.001 |
| Sme2.5_00827.1_g00014.1 | tropinone reductase homolog At1g07440-like | 28906.60 | 0.204 | 5 | 1.862 | 0.001 |
| Sme2.5_01754.1_g00001.1 | leucine-rich repeat receptor protein kinase EXS-like isoform 1 | 110471.60 | 0.057 | 4 | 2.133 | 0.033 |
| Sme2.5_00601.1_g00001.1 | sialyltransferase-like protein | 30863.42 | 0.107 | 2 | 1.617 | 0.016 |
| Sme2.5_02289.1_g00008.1 | alpha-galactosidase precursor | 45500.69 | 0.303 | 10 | 1.961 | 0.001 |
| Sme2.5_04937.1_g00002.1 | agamous-like MADS-box protein AGL61-like | 13234.40 | 0.068 | 1 | 10.000 | 0.001 |
| Sme2.5_04906.1_g00004.1 | Suberization-associated anionic peroxidase 2 | 38020.82 | 0.251 | 4 | 1.846 | 0.001 |
| Sme2.5_00216.1_g00002.1 | protein notum homolog | 46986.90 | 0.140 | 6 | 3.503 | 0.001 |
| Sme2.5_03368.1_g00010.1 | uncharacterized protein LOC101262737 | 37344.99 | 0.318 | 10 | 2.253 | 0.001 |
| Sme2.5_00019.1_g00028.1 | vicilin-like antimicrobial peptides 2-2-like | 51626.04 | 0.236 | 9 | 9.007 | 0.001 |
| Sme2.5_00662.1_g00003.1 | leucine aminopeptidase | 55740.67 | 0.521 | 13 | 1.816 | 0.001 |
| Sme2.5_00225.1_g00039.1 | beta-xylosidase/alpha-L-arabinofuranosidase 2 | 86074.00 | 0.342 | 18 | 1.718 | 0.001 |
| Sme2.5_10832.1_g00002.1 | probable glucan endo-1,3-beta-glucosidase A6-like | 49864.66 | 0.104 | 3 | 1.614 | 0.001 |
| Sme2.5_04773.1_g00002.1 | uncharacterized protein LOC101263984 | 17506.54 | 0.213 | 3 | 3.396 | 0.001 |
| Sme2.5_00785.1_g00002.1 | protein notum homolog | 38073.23 | 0.098 | 3 | 1.505 | 0.030 |
| Sme2.5_02296.1_g00004.1 | peroxidase 12-like | 39040.66 | 0.342 | 8 | 1.733 | 0.001 |
| Sme2.5_00161.1_g00003.1 | putative expansin | 25361.05 | 0.177 | 3 | 2.375 | 0.003 |
| Sme2.5_00323.1_g00016.1 | allene oxide syntase | 46562.67 | 0.615 | 11 | 2.325 | 0.001 |
| Sme2.5_03551.1_g00005.1 | cytosolic malate dehydrogenase | 65165.68 | 0.252 | 11 | 1.862 | 0.001 |
| Sme2.5_00211.1_g00004.1 | glutamine synthetase-like | 39237.49 | 0.104 | 2 | 3.824 | 0.026 |
| Sme2.5_03425.1_g00001.1 | probable beta-D-xylosidase 6-like | 89051.72 | 0.181 | 9 | 1.545 | 0.002 |
| Sme2.5_00915.1_g00003.1 | peptidyl-prolyl cis-trans isomerase-like | 18253.00 | 0.390 | 4 | 2.609 | 0.001 |
| Sme2.5_00188.1_g00020.1 | chalcone--flavonone isomerase-like | 23248.80 | 0.289 | 4 | 2.354 | 0.002 |
| Sme2.5_02680.1_g00001.1 | subtilisin-like protease-like | 78377.44 | 0.182 | 8 | 2.005 | 0.001 |
| Sme2.5_03556.1_g00005.1 | peroxidase 63-like | 37151.43 | 0.303 | 9 | 1.971 | 0.001 |
| Sme2.5_11682.1_g00001.1 | actin-depolymerizing factor 10-like | 16071.95 | 0.468 | 4 | 5.487 | 0.001 |
| Sme2.5_00662.1_g00001.1 | leucine aminopeptidase | 87185.64 | 0.190 | 5 | 2.727 | 0.001 |
| Sme2.5_00816.1_g00004.1 | mitochondrial outer membrane protein porin 2-like | 26406.85 | 0.518 | 6 | 1.905 | 0.007 |
| Sme2.5_02187.1_g00002.1 | pistil-specific extensin-like protein | 48531.74 | 0.088 | 2 | 6.352 | 0.001 |
| Sme2.5_18223.1_g00001.1 | aldehyde dehydrogenase family 2 member B7, mitochondrial-like | 43319.37 | 0.253 | 7 | 1.963 | 0.001 |
| Sme2.5_00250.1_g00006.1 | serine protease | 74815.14 | 0.030 | 2 | 1.980 | 0.001 |
| Sme2.5_00166.1_g00014.1 | actin-depolymerizing factor 12-like | 15828.80 | 0.073 | 1 | 6.806 | 0.045 |
| Sme2.5_01892.1_g00001.1 | aspartate aminotransferase | 54051.20 | 0.275 | 5 | 2.183 | 0.001 |
| Sme2.5_00086.1_g00010.1 | SlArf/Xyl3 | 88741.37 | 0.107 | 5 | 3.547 | 0.001 |
| Sme2.5_01362.1_g00013.1 | probable methyltransferase PMT21-like isoform 1 | 69336.65 | 0.082 | 3 | 2.474 | 0.022 |
| Sme2.5_00057.1_g00033.1 | pathogenesis-related protein PR-1 precursor | 20662.76 | 0.281 | 3 | 1.611 | 0.001 |
| Sme2.5_02441.1_g00001.1 | glucan endo-1,3-beta-glucosidase 5-like | 51594.93 | 0.108 | 3 | 1.828 | 0.002 |
| Sme2.5_00787.1_g00005.1 | KDEL-tailed cysteine endopeptidase CEP1-like | 33641.49 | 0.091 | 2 | 3.829 | 0.033 |
| Sme2.5_02982.1_g00007.1 | protein ASPARTIC PROTEASE IN GUARD CELL 2-like | 47880.23 | 0.411 | 13 | 1.546 | 0.001 |
| Sme2.5_04482.1_g00001.1 | NADH-cytochrome b5 reductase-like protein-like | 86416.80 | 0.115 | 7 | 1.882 | 0.001 |
| Sme2.5_14636.1_g00001.1 | 21 kDa protein-like | 21242.76 | 0.089 | 2 | 3.069 | 0.006 |
| Sme2.5_05146.1_g00001.1 | putative fructokinase-5-like | 34891.81 | 0.270 | 7 | 5.014 | 0.001 |
| Sme2.5_00317.1_g00002.1 | probable phospholipid hydroperoxide glutathione peroxidase | 27006.72 | 0.209 | 5 | 1.629 | 0.001 |
| Sme2.5_00723.1_g00008.1 | plastid lipid-associated protein 3, chloroplastic-like | 51093.06 | 0.329 | 13 | 2.761 | 0.001 |
| Sme2.5_01695.1_g00003.1 | aspartic proteinase-like protein 2-like | 54026.90 | 0.066 | 3 | 2.199 | 0.001 |
| Sme2.5_01118.1_g00002.1 | fasciclin-like arabinogalactan protein 17-like isoform 1 | 44504.15 | 0.271 | 4 | 1.540 | 0.001 |
| Sme2.5_00140.1_g00002.1 | ectonucleotide pyrophosphatase/phosphodiesterase family member 3-like | 54869.59 | 0.213 | 7 | 1.558 | 0.001 |
| Sme2.5_01556.1_g00002.1 | alpha-galactosidase-like | 49895.50 | 0.124 | 4 | 1.705 | 0.001 |
| Sme2.5_04205.1_g00007.1 | 10 kDa chaperonin-like | 22864.81 | 0.233 | 4 | 1.555 | 0.001 |
| Sme2.5_04696.1_g00006.1 | subtilisin-like protease-like | 65871.21 | 0.191 | 10 | 2.697 | 0.001 |
| Sme2.5_02505.1_g00002.1 | probably inactive leucine-rich repeat receptor-like protein kinase At2g25790-like | 52598.34 | 0.504 | 17 | 1.874 | 0.001 |
| Sme2.5_06455.1_g00005.1 | anther-specific protein LAT52-like | 19151.32 | 0.214 | 3 | 7.796 | 0.013 |
| Sme2.5_01406.1_g00007.1 | isovaleryl-CoA dehydrogenase 2, mitochondrial-like | 47092.88 | 0.059 | 2 | 2.082 | 0.037 |
| Sme2.5_10801.1_g00001.1 | UTP--glucose-1-phosphate uridylyltransferase-like | 47977.96 | 0.394 | 9 | 3.134 | 0.001 |
| Sme2.5_02289.1_g00002.1 | outer envelope pore protein 16-2, chloroplastic-like | 26453.36 | 0.103 | 2 | 4.562 | 0.022 |
| Sme2.5_04861.1_g00008.1 | SBT4B protein | 85114.44 | 0.237 | 6 | 1.781 | 0.001 |
| Sme2.5_00484.1_g00015.1 | primary amine oxidase | 80672.74 | 0.128 | 8 | 1.687 | 0.001 |
| Sme2.5_00386.1_g00008.1 | delta-1-pyrroline-5-carboxylate dehydrogenase 12A1, mitochondrial-like | 61565.59 | 0.472 | 20 | 1.637 | 0.001 |
| Sme2.5_01373.1_g00001.1 | probable inactive purple acid phosphatase 27-like, partial | 25609.37 | 0.120 | 3 | 2.399 | 0.031 |
| Sme2.5_01588.1_g00002.1 | gamma-glutamyltranspeptidase 3-like | 153918.30 | 0.174 | 18 | 1.525 | 0.001 |
| Sme2.5_02303.1_g00004.1 | calumenin-B-like | 33982.00 | 0.138 | 3 | 1.578 | 0.010 |
| Sme2.5_01553.1_g00005.1 | probable methyltransferase PMT27-like | 105247.70 | 0.147 | 9 | 4.905 | 0.001 |
| Sme2.5_00087.1_g00012.1 | constitutive plastid-lipid associated protein | 19863.15 | 0.317 | 5 | 1.609 | 0.001 |
| Sme2.5_12734.1_g00002.1 | DNA-damage-repair/toleration protein DRT100-like | 39634.13 | 0.207 | 7 | 3.733 | 0.001 |
| Sme2.5_00127.1_g00019.1 | mitochondrial outer membrane protein porin 2-like isoform 1 | 41353.47 | 0.326 | 7 | 1.566 | 0.001 |
| Sme2.5_06382.1_g00003.1 | alanine aminotransferase 2, mitochondrial-like | 60210.61 | 0.248 | 9 | 1.510 | 0.001 |
| Sme2.5_04696.1_g00001.1 | expansin11 precursor | 28233.41 | 0.156 | 4 | 2.020 | 0.001 |
| Sme2.5_05316.1_g00003.1 | UDP-Glc-4-epimerase | 38107.46 | 0.241 | 5 | 2.180 | 0.016 |
| Sme2.5_14718.1_g00002.1 | subtilisin-like endoprotease precursor | 71003.62 | 0.207 | 6 | 1.829 | 0.001 |
| Sme2.5_03087.1_g00003.1 | subtilisin-like protease-like | 77739.26 | 0.033 | 2 | 2.558 | 0.001 |
| Sme2.5_03531.1_g00001.1 | clathrin light chain 1-like | 30189.96 | 0.092 | 2 | 3.199 | 0.036 |
| Sme2.5_00001.1_g00016.1 | wound-inducible carboxypeptidase precursor | 50648.16 | 0.052 | 2 | 3.928 | 0.001 |
| Sme2.5_05308.1_g00001.1 | unknown | 39453.08 | 0.179 | 4 | 1.854 | 0.026 |
| Sme2.5_00077.1_g00002.1 | peptidyl-prolyl cis-trans isomerase FKBP62-like | 118048.60 | 0.078 | 5 | 2.247 | 0.001 |
| Sme2.5_00323.1_g00013.1 | allene oxide syntase | 29363.12 | 0.454 | 3 | 2.121 | 0.001 |
| Sme2.5_04022.1_g00007.1 | acid phosphatase 1-like | 29796.42 | 0.713 | 4 | 2.414 | 0.001 |
| Sme2.5_10874.1_g00002.1 | uncharacterized protein At5g39570-like | 34605.10 | 0.951 | 19 | 8.081 | 0.001 |
| Sme2.5_02749.1_g00005.1 | xyloglucan galactosyltransferase KATAMARI1 homolog | 51129.41 | 0.068 | 3 | 1.941 | 0.034 |
| Sme2.5_00100.1_g00022.1 | uncharacterized protein LOC101262834 | 82925.78 | 0.296 | 15 | 1.508 | 0.001 |
| Sme2.5_00420.1_g00014.1 | inducible plastid-lipid associated protein | 18825.40 | 0.560 | 7 | 4.340 | 0.001 |
| Sme2.5_04861.1_g00002.1 | subtilisin-like protease | 85361.77 | 0.232 | 8 | 1.579 | 0.001 |
| Sme2.5_29077.1_g00001.1 | Endochitinase 2 | 26159.00 | 0.159 | 2 | 1.868 | 0.001 |
| Sme2.5_05172.1_g00003.1 | non-specific lipid-transfer protein-like | 19715.84 | 0.367 | 5 | 1.501 | 0.002 |
| Sme2.5_00059.1_g00001.1 | hypothetical protein PRUPE_ppa003031mg | 36999.38 | 0.184 | 3 | 1.840 | 0.041 |
| Sme2.5_05516.1_g00004.1 | uncharacterized protein LOC101260262 | 39987.52 | 0.099 | 3 | 2.620 | 0.001 |
| Sme2.5_05678.1_g00003.1 | fasciclin-like arabinogalactan protein 2-like | 44354.68 | 0.358 | 10 | 2.971 | 0.001 |
| Sme2.5_00049.1_g00003.1 | early nodulin-like protein 1-like | 18748.54 | 0.179 | 2 | 1.952 | 0.007 |
| Sme2.5_02262.1_g00001.1 | fasciclin-like arabinogalactan protein 1-like | 43762.27 | 0.246 | 7 | 2.662 | 0.001 |
| Sme2.5_10351.1_g00002.1 | serpin-ZX-like | 44078.57 | 0.405 | 5 | 1.658 | 0.001 |
| Sme2.5_26344.1_g00001.1 | uncharacterized protein LOC101260800 | 55494.69 | 0.042 | 2 | 3.038 | 0.001 |
| Sme2.5_07790.1_g00002.1 | Polyprotein, putative | 14121.93 | 0.089 | 1 | 9.377 | 0.033 |
| Sme2.5_04212.1_g00001.1 | glyceraldehyde-3-phosphate dehydrogenase, cytosolic-like | 36518.75 | 0.442 | 7 | 5.432 | 0.001 |
| Sme2.5_12982.1_g00001.1 | ACC oxidase | 36513.54 | 0.241 | 4 | 2.005 | 0.005 |
| Sme2.5_13307.1_g00002.1 | probable inactive purple acid phosphatase 27-like | 49929.94 | 0.499 | 5 | 1.988 | 0.001 |
| Sme2.5_00499.1_g00004.1 | glutamate decarboxylase isoform2 | 60122.57 | 0.408 | 14 | 1.531 | 0.001 |
| Sme2.5_06504.1_g00005.1 | gamma carbonic anhydrase-like 2, mitochondrial-like | 27902.62 | 0.286 | 6 | 1.579 | 0.004 |
| Sme2.5_10236.1_g00004.1 | flavoprotein WrbA-like | 21615.87 | 0.591 | 6 | 1.834 | 0.001 |
| Sme2.5_02584.1_g00008.1 | 8-hydroxygeraniol dehydrogenase | 39249.74 | 0.231 | 3 | 1.961 | 0.042 |
| Sme2.5_09669.1_g00005.1 | beta-glucosidase 40-like | 57747.89 | 0.222 | 11 | 3.877 | 0.001 |
| Sme2.5_00172.1_g00029.1 | uncharacterized protein LOC101252745 | 112047.00 | 0.087 | 8 | 1.581 | 0.001 |
| Sme2.5_02176.1_g00010.1 | carbonic anhydrase | 29774.10 | 0.116 | 3 | 2.694 | 0.001 |
| Sme2.5_03491.1_g00003.1 | aldose 1-epimerase-like isoform 1 | 39110.36 | 0.177 | 4 | 3.049 | 0.001 |
| Sme2.5_31247.1_g00001.1 | hypothetical protein VITISV_027379 | 23536.02 | 0.173 | 1 | 2.614 | 0.006 |
| Sme2.5_00813.1_g00013.1 | uncharacterized protein LOC101258525 | 92237.69 | 0.035 | 3 | 2.152 | 0.010 |
| Sme2.5_04037.1_g00005.1 | glutelin type-A 2-like | 38488.82 | 0.475 | 8 | 3.566 | 0.001 |
| Sme2.5_00983.1_g00014.1 | unknown | 16202.98 | 0.215 | 3 | 2.403 | 0.001 |
| Sme2.5_00323.1_g00015.1 | allene oxide syntase | 53858.84 | 0.055 | 2 | 1.747 | 0.001 |
| Sme2.5_01731.1_g00001.1 | phospholipase A1-II 1-like isoform 1 | 44138.36 | 0.756 | 11 | 2.621 | 0.001 |
| Sme2.5_00076.1_g00016.1 | aspartic proteinase nepenthesin-2-like isoform 1 | 50322.82 | 0.190 | 6 | 3.399 | 0.001 |
| Sme2.5_04312.1_g00006.1 | uncharacterized protein LOC101247696 | 57087.38 | 0.044 | 2 | 4.058 | 0.011 |
| Sme2.5_00845.1_g00001.1 | Beta-D-glucosidase | 64682.9 | 0.114 | 2 | 1.635 | 0.041 |
