# Supplementary material for: Comparative proteomic analysis of eggplant (Solanum melongena L.) heterostylous pistil development
Source: PLoS One. 2017 Jun 6;12(6):e0179018. doi: 10.1371/journal.pone.0179018 (PMC5460878; doi:10.1371/journal.pone.0179018)
Supplement: S3 Table — (DOCX) [file pone.0179018.s008.docx]

**Table S3 Downregulated proteins in pistils of L-morph flowers during maturity with a 1.5-fold change compared with developmental stage**

| **Protein_ID** | **Description** | **Mass** | **Coverage** | **Peptide** | **Fold change** | **Qvalue** |
| --- | --- | --- | --- | --- | --- | --- |
| Sme2.5_04880.1_g00004.1 | hypothetical protein PRUPE_ppa002546mg | 28764.55 | 0.208 | 5 | 0.590 | 0.001 |
| Sme2.5_01418.1_g00010.1 | 60S ribosomal protein L13-2-like | 23655.96 | 0.364 | 3 | 0.604 | 0.032 |
| Sme2.5_24492.1_g00002.1 | sulfur | 36086.98 | 0.239 | 6 | 0.601 | 0.004 |
| Sme2.5_00226.1_g00008.1 | deacetylase-like protein | 33633.09 | 0.149 | 5 | 0.615 | 0.001 |
| Sme2.5_00406.1_g00017.1 | uncharacterized protein LOC101251618 isoform 2 | 39956.94 | 0.248 | 8 | 0.549 | 0.001 |
| Sme2.5_04286.1_g00005.1 | chloroplast polyphenol oxidase precursor | 30827.24 | 0.138 | 2 | 0.572 | 0.001 |
| Sme2.5_00280.1_g00001.1 | 40S ribosomal protein S3-3-like | 26376.14 | 0.631 | 12 | 0.604 | 0.001 |
| Sme2.5_05137.1_g00005.1 | ketol-acid reductoisomerase, chloroplastic-like | 63615.31 | 0.159 | 4 | 0.582 | 0.001 |
| Sme2.5_05088.1_g00003.1 | replication factor C subunit 1-like | 42067.14 | 0.123 | 3 | 0.595 | 0.035 |
| Sme2.5_00236.1_g00008.1 | uncharacterized protein LOC101244097 | 68016.65 | 0.051 | 3 | 0.598 | 0.010 |
| Sme2.5_04790.1_g00002.1 | PSII 47kDa protein | 78067.33 | 0.020 | 1 | 0.620 | 0.001 |
| Sme2.5_00080.1_g00004.1 | uncharacterized GPI-anchored protein At1g27950-like | 25539.65 | 0.085 | 2 | 0.212 | 0.003 |
| Sme2.5_03475.1_g00009.1 | nucleolar protein 56-like | 62323.45 | 0.216 | 8 | 0.662 | 0.009 |
| Sme2.5_00204.1_g00004.1 | uncharacterized protein LOC101245557 | 26021.23 | 0.274 | 4 | 0.329 | 0.012 |
| Sme2.5_00512.1_g00006.1 | ruBisCO large subunit-binding protein subunit alpha, chloroplastic-like | 62339.15 | 0.447 | 17 | 0.636 | 0.001 |
| Sme2.5_01868.1_g00001.1 | ruvB-like 2-like | 52108.84 | 0.070 | 3 | 0.515 | 0.001 |
| Sme2.5_01102.1_g00001.1 | cullin-associated NEDD8-dissociated protein 1-like | 222514.00 | 0.038 | 6 | 0.600 | 0.001 |
| Sme2.5_03566.1_g00005.1 | adenylosuccinate lyase-like | 54119.85 | 0.048 | 2 | 0.533 | 0.010 |
| Sme2.5_06815.1_g00001.1 | spermidine synthase | 45811.67 | 0.259 | 8 | 0.639 | 0.001 |
| Sme2.5_02386.1_g00003.1 | carbonic anhydrase, partial | 35093.85 | 0.208 | 6 | 0.653 | 0.022 |
| Sme2.5_00864.1_g00011.1 | uncharacterized protein LOC101258386 | 92906.82 | 0.052 | 4 | 0.518 | 0.001 |
| Sme2.5_03906.1_g00010.1 | bifunctional monodehydroascorbate reductase and carbonic anhydrase nectarin-3-like | 29618.23 | 0.110 | 1 | 0.412 | 0.012 |
| Sme2.5_03252.1_g00006.1 | heat shock protein 83-like | 81455.33 | 0.349 | 5 | 0.505 | 0.001 |
| Sme2.5_00034.1_g00013.1 | transcription elongation factor SPT6-like | 186224.40 | 0.031 | 5 | 0.558 | 0.001 |
| Sme2.5_02996.1_g00005.1 | uncharacterized protein LOC101246515 | 104188.80 | 0.084 | 7 | 0.622 | 0.004 |
| Sme2.5_01489.1_g00008.1 | CASP-like protein RCOM_1206790-like | 19354.34 | 0.155 | 3 | 0.563 | 0.009 |
| Sme2.5_06238.1_g00003.1 | heat shock cognate protein 80 | 84351.99 | 0.418 | 1 | 0.536 | 0.001 |
| Sme2.5_00488.1_g00017.1 | 40S ribosomal protein S24-2-like isoform 1 | 15970.84 | 0.341 | 2 | 0.561 | 0.001 |
| Sme2.5_05225.1_g00004.1 | asparagine--tRNA ligase, cytoplasmic 1 | 67139.50 | 0.276 | 15 | 0.650 | 0.001 |
| Sme2.5_00100.1_g00018.1 | putative 40S ribosomal protein S8-like protein | 24962.45 | 0.491 | 2 | 0.568 | 0.001 |
| Sme2.5_01559.1_g00002.1 | Histone H1 | 30859.06 | 0.268 | 6 | 0.229 | 0.001 |
| Sme2.5_04171.1_g00002.1 | Polyprotein, putative | 58993.61 | 0.073 | 1 | 0.453 | 0.003 |
| Sme2.5_03753.1_g00003.1 | osmotin-like protein precursor | 27903.35 | 0.120 | 3 | 0.393 | 0.003 |
| Sme2.5_00650.1_g00013.1 | DNA replication licensing factor mcm4-like | 98128.02 | 0.116 | 9 | 0.542 | 0.007 |
| Sme2.5_00499.1_g00025.1 | 60S ribosomal protein L6-like | 25441.33 | 0.314 | 5 | 0.506 | 0.001 |
| Sme2.5_09735.1_g00002.1 | DNA-directed RNA polymerases I and III subunit RPAC1-like | 43602.60 | 0.062 | 2 | 0.624 | 0.045 |
| Sme2.5_01775.1_g00002.1 | DNA replication licensing factor mcm5-A-like | 82403.40 | 0.091 | 5 | 0.644 | 0.026 |
| Sme2.5_05121.1_g00005.1 | phospho-2-dehydro-3-deoxyheptonate aldolase 2, chloroplastic | 60074.43 | 0.267 | 8 | 0.611 | 0.001 |
| Sme2.5_04224.1_g00007.1 | phospholipase A1-IIgamma-like | 45100.01 | 0.186 | 3 | 0.583 | 0.038 |
| Sme2.5_04111.1_g00005.1 | citrate binding protein | 21111.52 | 0.228 | 4 | 0.378 | 0.001 |
| Sme2.5_06540.1_g00005.1 | ATP synthase CF1 alpha chain | 32186.68 | 0.322 | 8 | 0.588 | 0.001 |
| Sme2.5_03836.1_g00005.1 | ubiquitin extension protein | 17859.54 | 0.391 | 1 | 0.429 | 0.001 |
| Sme2.5_01046.1_g00003.1 | predicted protein | 19639.91 | 0.043 | 1 | 0.133 | 0.041 |
| Sme2.5_04984.1_g00003.1 | proteinase inhibitor II | 25168.62 | 0.333 | 5 | 0.123 | 0.001 |
| Sme2.5_03463.1_g00002.1 | protein PROLIFERA-like | 81201.48 | 0.104 | 6 | 0.540 | 0.014 |
| Sme2.5_00234.1_g00013.1 | calmodulin-related protein isoform 4 | 16875.90 | 0.617 | 1 | 0.629 | 0.003 |
| Sme2.5_05182.1_g00002.1 | Ribulose bisphosphate carboxylase small chain 3, chloroplastic | 20620.23 | 0.544 | 2 | 0.513 | 0.001 |
| Sme2.5_18962.1_g00001.1 | annexin D5-like | 35768.49 | 0.095 | 3 | 0.582 | 0.028 |
| Sme2.5_01185.1_g00005.1 | endoplasmin homolog | 92419.47 | 0.373 | 16 | 0.597 | 0.001 |
| Sme2.5_00146.1_g00009.1 | UDP-glucuronate decarboxylase 1 | 39063.07 | 0.493 | 3 | 0.616 | 0.001 |
| Sme2.5_00538.1_g00008.1 | 60S ribosomal protein L23a-like | 17457.84 | 0.266 | 2 | 0.446 | 0.003 |
| Sme2.5_00577.1_g00007.1 | N-alpha-acetyltransferase 16, NatA auxiliary subunit-like | 17406.05 | 0.196 | 3 | 0.562 | 0.019 |
| Sme2.5_02104.1_g00006.1 | uncharacterized protein LOC101260453 | 21674.39 | 0.111 | 1 | 0.193 | 0.001 |
| Sme2.5_02262.1_g00005.1 | uncharacterized protein LOC101250613 | 46238.49 | 0.253 | 9 | 0.420 | 0.001 |
| Sme2.5_08458.1_g00002.1 | 60S ribosomal protein L26-1-like | 16707.14 | 0.212 | 1 | 0.444 | 0.043 |
| Sme2.5_16412.1_g00001.1 | Kunitz-type enzyme inhibitor S9C11 | 21996.90 | 0.365 | 6 | 0.650 | 0.001 |
| Sme2.5_01494.1_g00003.1 | 60S ribosomal protein L19-2-like | 24901.74 | 0.299 | 2 | 0.516 | 0.025 |
| Sme2.5_00001.1_g00048.1 | cinnamic acid 4-hydroxylase | 58331.85 | 0.244 | 12 | 0.638 | 0.001 |
| Sme2.5_11618.1_g00003.1 | fasciclin-like arabinogalactan protein 2-like | 42408.78 | 0.086 | 3 | 0.400 | 0.001 |
| Sme2.5_00287.1_g00018.1 | uncharacterized protein LOC101256524 | 46399.81 | 0.055 | 2 | 0.498 | 0.006 |
| Sme2.5_02364.1_g00010.1 | H/ACA ribonucleoprotein complex subunit 4-like | 66778.28 | 0.229 | 10 | 0.381 | 0.001 |
| Sme2.5_00172.1_g00018.1 | beta-catenin-like protein 1-like | 58017.79 | 0.024 | 2 | 0.591 | 0.032 |
| Sme2.5_01509.1_g00012.1 | uncharacterized protein LOC101262922 | 76868.59 | 0.012 | 1 | 0.377 | 0.022 |
| Sme2.5_03682.1_g00010.1 | uncharacterized protein LOC101301900 | 81117.02 | 0.103 | 5 | 0.406 | 0.001 |
| Sme2.5_00557.1_g00002.1 | serine protease inhibitor 1-like | 24440.41 | 0.262 | 4 | 0.576 | 0.001 |
| Sme2.5_01731.1_g00002.1 | phospholipase A1-II 1-like isoform 1 | 45216.21 | 0.722 | 13 | 0.473 | 0.001 |
| Sme2.5_12564.1_g00002.1 | 60S acidic ribosomal protein P3-like | 12046.64 | 0.378 | 3 | 0.450 | 0.023 |
| Sme2.5_00813.1_g00006.1 | serrate RNA effector molecule-like | 92548.35 | 0.072 | 5 | 0.520 | 0.001 |
| Sme2.5_05570.1_g00003.1 | UDP-glucuronic acid decarboxylase 1-like | 39275.30 | 0.269 | 2 | 0.623 | 0.045 |
| Sme2.5_00020.1_g00016.1 | dioxygenase | 38246.35 | 0.367 | 10 | 0.518 | 0.001 |
| Sme2.5_00142.1_g00011.1 | pullulanase 1, chloroplastic-like | 109731.80 | 0.007 | 1 | 0.356 | 0.017 |
| Sme2.5_12551.1_g00002.1 | cycloartenol synthase | 34947.44 | 0.132 | 3 | 0.465 | 0.009 |
| Sme2.5_01441.1_g00007.1 | 40S ribosomal protein S6-like | 29900.16 | 0.336 | 3 | 0.581 | 0.001 |
| Sme2.5_03020.1_g00006.1 | DEAD-box ATP-dependent RNA helicase 56-like | 77372.46 | 0.126 | 5 | 0.634 | 0.001 |
| Sme2.5_01746.1_g00006.1 | putative cytochrome P450 | 58934.91 | 0.137 | 6 | 0.488 | 0.014 |
| Sme2.5_00989.1_g00005.1 | uncharacterized protein C167.05-like | 39152.82 | 0.167 | 3 | 0.663 | 0.001 |
| Sme2.5_13136.1_g00002.1 | unknown | 32007.07 | 0.051 | 2 | 0.402 | 0.001 |
| Sme2.5_02055.1_g00002.1 | probable fructose-bisphosphate aldolase 2, chloroplastic-like | 42266.70 | 0.345 | 8 | 0.572 | 0.001 |
| Sme2.5_01027.1_g00011.1 | Hop-interacting protein THI016 | 65690.37 | 0.177 | 7 | 0.584 | 0.045 |
| Sme2.5_00401.1_g00012.1 | signal recognition particle 43 kDa protein, chloroplastic-like | 41525.89 | 0.051 | 2 | 0.404 | 0.029 |
| Sme2.5_30574.1_g00001.1 | early nodulin-like protein 1-like | 19644.13 | 0.350 | 6 | 0.492 | 0.001 |
| Sme2.5_06695.1_g00003.1 | pathogenesis-related protein 10 | 17742.05 | 0.900 | 12 | 0.593 | 0.001 |
| Sme2.5_01339.1_g00003.1 | glutamyl-tRNA(Gln) amidotransferase subunit B, chloroplastic/mitochondrial-like | 79674.82 | 0.065 | 4 | 0.588 | 0.009 |
| Sme2.5_00733.1_g00012.1 | uncharacterized protein LOC101249817 | 189000.30 | 0.111 | 14 | 0.616 | 0.001 |
| Sme2.5_01489.1_g00004.1 | AGO1A | 118852.20 | 0.156 | 8 | 0.574 | 0.001 |
| Sme2.5_00048.1_g00024.1 | 28 kDa ribonucleoprotein, chloroplastic-like | 34786.98 | 0.256 | 6 | 0.651 | 0.001 |
| Sme2.5_00018.1_g00002.1 | cinnamoyl-CoA reductase | 30224.31 | 0.114 | 3 | 0.616 | 0.011 |
| Sme2.5_02902.1_g00005.1 | glyceraldehyde-3-phosphate dehydrogenase A, chloroplastic-like | 43062.47 | 0.471 | 11 | 0.601 | 0.001 |
| Sme2.5_00444.1_g00001.1 | uncharacterized protein LOC101252108 | 240985.30 | 0.078 | 12 | 0.588 | 0.001 |
| Sme2.5_00204.1_g00016.1 | uncharacterized protein LOC101264938 | 55005.16 | 0.160 | 4 | 0.575 | 0.020 |
| Sme2.5_01239.1_g00002.1 | ATP-citrate synthase alpha chain protein 3-like | 46908.01 | 0.182 | 4 | 0.652 | 0.020 |
| Sme2.5_05238.1_g00005.1 | 60S ribosomal protein L6-like | 25493.32 | 0.306 | 5 | 0.525 | 0.001 |
| Sme2.5_00551.1_g00006.1 | 14-3-3 protein 4 | 34114.17 | 0.298 | 3 | 0.657 | 0.024 |
| Sme2.5_02153.1_g00010.1 | ATP-dependent RNA helicase DBP2-like isoform 1 | 39029.94 | 0.143 | 3 | 0.628 | 0.046 |
| Sme2.5_00858.1_g00005.1 | glycine cleavage system H protein, mitochondrial-like isoform 1 | 17541.57 | 0.099 | 1 | 0.653 | 0.001 |
| Sme2.5_17229.1_g00001.1 | putative F-box protein PP2-B12-like | 20279.09 | 0.609 | 8 | 0.325 | 0.001 |
| Sme2.5_06441.1_g00003.1 | importin-5-like | 124467.90 | 0.031 | 3 | 0.619 | 0.020 |
| Sme2.5_00079.1_g00013.1 | deoxyuridine 5'-triphosphate nucleotidohydrolase | 18221.51 | 0.616 | 7 | 0.536 | 0.001 |
| Sme2.5_04544.1_g00001.1 | expansin9 precursor | 28711.31 | 0.035 | 1 | 0.631 | 0.004 |
| Sme2.5_00942.1_g00003.1 | ribosomal protein S14-like protein | 16373.64 | 0.367 | 1 | 0.619 | 0.042 |
| Sme2.5_06367.1_g00005.1 | GDSL esterase/lipase At1g29670-like | 24032.58 | 0.056 | 1 | 0.634 | 0.001 |
| Sme2.5_03742.1_g00003.1 | Glycosyl hydrolases family 17 protein | 44096.32 | 0.087 | 3 | 0.311 | 0.001 |
| Sme2.5_00925.1_g00002.1 | bifunctional dihydrofolate reductase-thymidylate synthase-like | 56301.24 | 0.174 | 6 | 0.660 | 0.001 |
| Sme2.5_00065.1_g00007.1 | aspartic proteinase oryzasin-1-like | 50820.41 | 0.239 | 8 | 0.504 | 0.001 |
| Sme2.5_00372.1_g00003.1 | ruBisCO large subunit-binding protein subunit beta, chloroplastic-like | 66207.71 | 0.320 | 10 | 0.553 | 0.001 |
| Sme2.5_02308.1_g00009.1 | tetraketide alpha-pyrone reductase 1-like | 30386.58 | 0.343 | 7 | 0.432 | 0.001 |
| Sme2.5_05137.1_g00003.1 | 14-3-3 protein 8 | 29706.61 | 0.308 | 5 | 0.626 | 0.041 |
| Sme2.5_00368.1_g00010.1 | 4-coumarate--CoA ligase-like 1-like | 61656.04 | 0.215 | 9 | 0.476 | 0.001 |
| Sme2.5_03352.1_g00006.1 | nuclear-pore anchor-like | 231317.80 | 0.053 | 9 | 0.602 | 0.002 |
| Sme2.5_12406.1_g00002.1 | GMP synthase [glutamine-hydrolyzing]-like | 59773.79 | 0.133 | 6 | 0.664 | 0.006 |
| Sme2.5_30033.1_g00001.1 | unknown | 22168.66 | 0.345 | 2 | 0.606 | 0.010 |
| Sme2.5_02518.1_g00007.1 | putative arginine/serine-rich protein-like | 47269.20 | 0.106 | 5 | 0.572 | 0.001 |
| Sme2.5_06227.1_g00004.1 | deoxyuridine 5'-triphosphate nucleotidohydrolase-like | 15687.20 | 0.827 | 8 | 0.655 | 0.001 |
| Sme2.5_01164.1_g00001.1 | omega-hydroxypalmitate O-feruloyl transferase-like | 42735.54 | 0.222 | 7 | 0.353 | 0.001 |
| Sme2.5_01772.1_g00003.1 | chaperone protein ClpB1-like | 101415.30 | 0.065 | 4 | 0.629 | 0.047 |
| Sme2.5_29237.1_g00001.1 | protein SGT1 homolog | 19968.19 | 0.251 | 4 | 0.645 | 0.004 |
| Sme2.5_01098.1_g00016.1 | sister chromatid cohesion protein PDS5 homolog B-like | 124102.60 | 0.040 | 4 | 0.619 | 0.042 |
| Sme2.5_00230.1_g00013.1 | uncharacterized protein LOC101254818 | 159514.50 | 0.014 | 2 | 0.481 | 0.012 |
| Sme2.5_00281.1_g00013.1 | ribosomal protein L3 | 44797.00 | 0.393 | 8 | 0.436 | 0.001 |
| Sme2.5_02142.1_g00005.1 | uncharacterized protein LOC101259467 | 51818.75 | 0.402 | 13 | 0.604 | 0.001 |
| Sme2.5_30505.1_g00001.1 | acyltransferase-like | 11395.81 | 0.275 | 2 | 0.543 | 0.049 |
| Sme2.5_01085.1_g00002.1 | probable leucine-rich repeat receptor-like protein kinase At1g35710-like | 71005.38 | 0.224 | 11 | 0.389 | 0.001 |
| Sme2.5_01887.1_g00007.1 | uncharacterized protein LOC101255308 | 143027.90 | 0.033 | 4 | 0.627 | 0.001 |
| Sme2.5_05872.1_g00005.1 | SUMO-activating enzyme subunit 2-like | 53644.02 | 0.111 | 5 | 0.604 | 0.015 |
| Sme2.5_00066.1_g00013.1 | glycine-rich protein precursor | 15485.18 | 0.144 | 1 | 0.527 | 0.003 |
| Sme2.5_00155.1_g00006.1 | uncharacterized protein LOC101260626 | 18251.51 | 0.133 | 2 | 0.348 | 0.016 |
| Sme2.5_01616.1_g00002.1 | probable cinnamyl alcohol dehydrogenase 6-like | 32370.95 | 0.059 | 2 | 0.501 | 0.006 |
| Sme2.5_08303.1_g00002.1 | phospholipase D alpha 1-like | 92791.53 | 0.122 | 7 | 0.653 | 0.001 |
| Sme2.5_00889.1_g00001.1 | Aqp2 protein | 30783.84 | 0.204 | 2 | 0.396 | 0.001 |
| Sme2.5_10995.1_g00001.1 | omega-hydroxypalmitate O-feruloyl transferase-like | 47840.38 | 0.211 | 5 | 0.540 | 0.047 |
| Sme2.5_01937.1_g00008.1 | xyloglucan endotransglucosylase-hydrolase XTH7 | 33377.36 | 0.205 | 4 | 0.565 | 0.001 |
| Sme2.5_05072.1_g00005.1 | phosphoribulokinase, chloroplastic-like | 47521.18 | 0.297 | 10 | 0.626 | 0.001 |
| Sme2.5_08818.1_g00004.1 | serine/arginine-rich splicing factor RSZ21A-like | 29554.90 | 0.190 | 5 | 0.594 | 0.001 |
| Sme2.5_00099.1_g00017.1 | hypothetical protein 111O18.18 | 64794.04 | 0.121 | 6 | 0.481 | 0.001 |
| Sme2.5_00210.1_g00007.1 | bifunctional aspartokinase/homoserine dehydrogenase, chloroplastic-like | 31671.28 | 0.094 | 2 | 0.596 | 0.017 |
| Sme2.5_01411.1_g00008.1 | phosphatidylinositide phosphatase SAC1-like | 127406.90 | 0.097 | 7 | 0.520 | 0.001 |
| Sme2.5_06581.1_g00002.1 | uncharacterized protein LOC101262658 | 43007.84 | 0.093 | 3 | 0.498 | 0.015 |
| Sme2.5_06157.1_g00003.1 | 60S ribosomal protein L19-2-like | 25150.85 | 0.306 | 2 | 0.310 | 0.001 |
| Sme2.5_00163.1_g00002.1 | protein argonaute 4-like | 105854.50 | 0.264 | 14 | 0.598 | 0.001 |
| Sme2.5_00789.1_g00009.1 | valine--tRNA ligase-like | 134613.10 | 0.077 | 7 | 0.458 | 0.001 |
| Sme2.5_00512.1_g00007.1 | bifunctional monodehydroascorbate reductase and carbonic anhydrase nectarin-3-like | 28298.46 | 0.586 | 9 | 0.540 | 0.001 |
| Sme2.5_00846.1_g00008.1 | eukaryotic initiation factor 4A-3-like | 45045.43 | 0.232 | 8 | 0.582 | 0.001 |
| Sme2.5_01484.1_g00002.1 | uncharacterized protein LOC101265598 isoform 1 | 52350.53 | 0.088 | 3 | 0.360 | 0.001 |
| Sme2.5_00588.1_g00013.1 | uncharacterized protein LOC101253861 | 27398.90 | 0.232 | 4 | 0.652 | 0.005 |
| Sme2.5_00346.1_g00019.1 | chalcone synthase-like | 44007.60 | 0.295 | 8 | 0.589 | 0.009 |
| Sme2.5_06072.1_g00001.1 | uncharacterized protein LOC101251468 | 23331.86 | 0.164 | 3 | 0.582 | 0.013 |
| Sme2.5_00088.1_g00007.1 | calmodulin isoform 1 | 9456.53 | 0.815 | 2 | 0.635 | 0.017 |
| Sme2.5_03095.1_g00004.1 | eukaryotic peptide chain release factor subunit 1-3-like | 49130.92 | 0.103 | 4 | 0.617 | 0.001 |
| Sme2.5_00140.1_g00009.1 | ArcA2 protein-like | 81059.13 | 0.177 | 4 | 0.639 | 0.004 |
| Sme2.5_00290.1_g00001.1 | cellulose synthase-like protein G1-like | 79250.81 | 0.122 | 7 | 0.564 | 0.012 |
| Sme2.5_06426.1_g00002.1 | peroxidase 40-like isoform 1 | 64534.19 | 0.065 | 3 | 0.443 | 0.007 |
| Sme2.5_00388.1_g00009.1 | LRR receptor-like serine/threonine-protein kinase FLS2-like | 45788.49 | 0.363 | 10 | 0.180 | 0.001 |
| Sme2.5_22373.1_g00002.1 | proteinase inhibitor type-2 CEVI57 precursor | 24139.72 | 0.114 | 2 | 0.412 | 0.014 |
| Sme2.5_01611.1_g00010.1 | H1 histone-like protein | 21647.19 | 0.095 | 2 | 0.340 | 0.002 |
| Sme2.5_06520.1_g00003.1 | uncharacterized protein LOC101263366 | 22320.27 | 0.514 | 8 | 0.608 | 0.001 |
| Sme2.5_04651.1_g00003.1 | mediator-associated protein 1-like | 43289.34 | 0.324 | 9 | 0.426 | 0.001 |
| Sme2.5_04937.1_g00006.1 | AGO4A | 98736.61 | 0.069 | 3 | 0.625 | 0.001 |
| Sme2.5_03391.1_g00008.1 | lignin-forming anionic peroxidase-like | 32972.67 | 0.147 | 3 | 0.481 | 0.001 |
| Sme2.5_02223.1_g00002.1 | selenoprotein H-like | 17212.35 | 0.196 | 3 | 0.451 | 0.001 |
| Sme2.5_00950.1_g00002.1 | transformer-SR ribonucleoprotein | 28928.77 | 0.170 | 2 | 0.531 | 0.011 |
| Sme2.5_02984.1_g00002.1 | polyubiquitin-like | 44423.86 | 0.016 | 1 | 0.387 | 0.001 |
| Sme2.5_00622.1_g00006.1 | chaperone protein dnaJ 6-like | 35315.73 | 0.167 | 4 | 0.568 | 0.021 |
| Sme2.5_07093.1_g00002.1 | 9-cis-epoxycarotenoid dioxygenase NCED6, chloroplastic-like | 57498.87 | 0.014 | 1 | 0.229 | 0.001 |
| Sme2.5_03583.1_g00003.1 | uncharacterized protein LOC101252226 | 146888.60 | 0.165 | 15 | 0.512 | 0.001 |
| Sme2.5_00210.1_g00012.1 | bifunctional aspartokinase/homoserine dehydrogenase, chloroplastic-like | 20631.45 | 0.384 | 5 | 0.598 | 0.013 |
| Sme2.5_06770.1_g00003.1 | putative nuclear matrix constituent protein 1-like protein-like | 134056.10 | 0.071 | 8 | 0.541 | 0.002 |
| Sme2.5_00292.1_g00003.1 | Histone deacetylase HDT1 | 30081.73 | 0.263 | 4 | 0.650 | 0.030 |
| Sme2.5_08706.1_g00002.1 | uncharacterized protein LOC101252351 | 96719.25 | 0.117 | 7 | 0.528 | 0.001 |
| Sme2.5_02552.1_g00001.1 | 26S proteasome non-ATPase regulatory subunit 13-like | 71809.01 | 0.078 | 4 | 0.628 | 0.001 |
| Sme2.5_04436.1_g00001.1 | LOW QUALITY PROTEIN: DNA-directed RNA polymerase E subunit 1 | 240993.00 | 0.053 | 9 | 0.608 | 0.010 |
| Sme2.5_04375.1_g00005.1 | tubulin beta-1 chain-like | 48482.96 | 0.409 | 1 | 0.322 | 0.002 |
| Sme2.5_10388.1_g00001.1 | probable tRNA threonylcarbamoyladenosine biosynthesis protein OSGEP-like | 33372.61 | 0.246 | 5 | 0.587 | 0.004 |
| Sme2.5_02983.1_g00003.1 | eukaryotic translation initiation factor 3 subunit L-like | 22649.59 | 0.103 | 2 | 0.417 | 0.033 |
| Sme2.5_05198.1_g00003.1 | AGO5 | 112537.10 | 0.113 | 8 | 0.617 | 0.002 |
| Sme2.5_01772.1_g00001.1 | glucan endo-1,3-beta-glucosidase-like protein 3-like | 19333.22 | 0.048 | 1 | 0.405 | 0.006 |
| Sme2.5_06963.1_g00004.1 | protein TIC 40, chloroplastic-like | 11050.26 | 0.273 | 2 | 0.588 | 0.009 |
| Sme2.5_01602.1_g00010.1 | importin subunit alpha-1a-like | 38485.92 | 0.314 | 7 | 0.656 | 0.001 |
| Sme2.5_04107.1_g00007.1 | patellin-4-like | 54566.21 | 0.168 | 6 | 0.474 | 0.025 |
| Sme2.5_05323.1_g00006.1 | glutamate-1-semialdehyde 2,1-aminomutase, chloroplastic | 20132.37 | 0.139 | 2 | 0.646 | 0.024 |
| Sme2.5_02262.1_g00003.1 | uncharacterized protein LOC101251491 | 74554.96 | 0.149 | 7 | 0.647 | 0.012 |
| Sme2.5_00563.1_g00024.1 | replication factor C subunit 4-like | 39892.07 | 0.093 | 3 | 0.651 | 0.019 |
| Sme2.5_06732.1_g00001.1 | probable histone H2A variant 3-like | 20463.06 | 0.183 | 1 | 0.635 | 0.003 |
| Sme2.5_03640.1_g00006.1 | 40S ribosomal protein S17-like | 16221.71 | 0.556 | 3 | 0.651 | 0.001 |
| Sme2.5_03213.1_g00011.1 | uncharacterized protein LOC101264258 isoform 1 | 38948.28 | 0.101 | 2 | 0.431 | 0.001 |
| Sme2.5_05426.1_g00003.1 | 33 kDa ribonucleoprotein, chloroplastic-like | 32195.51 | 0.201 | 4 | 0.645 | 0.017 |
| Sme2.5_01235.1_g00007.1 | chlorophyll a-b binding protein CP29.2, chloroplastic-like | 31230.17 | 0.361 | 7 | 0.650 | 0.001 |
| Sme2.5_00594.1_g00001.1 | 60S ribosomal protein L10 | 24698.94 | 0.250 | 3 | 0.521 | 0.001 |
| Sme2.5_01772.1_g00005.1 | 26S protease regulatory subunit 8 homolog A-like | 49304.71 | 0.405 | 12 | 0.628 | 0.001 |
| Sme2.5_14955.1_g00001.1 | uncharacterized protein LOC101263689 | 91161.18 | 0.046 | 1 | 0.400 | 0.001 |
| Sme2.5_00064.1_g00002.1 | T-complex protein 1 subunit zeta-like | 62244.22 | 0.254 | 5 | 0.601 | 0.001 |
| Sme2.5_00226.1_g00031.1 | 40S ribosomal protein S23-like | 15190.35 | 0.228 | 3 | 0.555 | 0.001 |
| Sme2.5_02632.1_g00003.1 | probable splicing factor 3A subunit 1-like | 141365.80 | 0.088 | 9 | 0.661 | 0.001 |
| Sme2.5_00584.1_g00004.1 | hypothetical protein VITISV_032012 | 41224.09 | 0.024 | 1 | 0.568 | 0.021 |
| Sme2.5_00151.1_g00009.1 | unnamed protein product | 12388.67 | 0.142 | 2 | 0.363 | 0.001 |
| Sme2.5_07665.1_g00001.1 | zerumbone synthase-like | 33466.47 | 0.535 | 10 | 0.616 | 0.001 |
| Sme2.5_01918.1_g00005.1 | apyrase-like | 47431.48 | 0.302 | 10 | 0.598 | 0.001 |
| Sme2.5_02811.1_g00002.1 | uncharacterized protein LOC101245558 | 113730.80 | 0.171 | 12 | 0.651 | 0.001 |
| Sme2.5_15649.1_g00003.1 | unknown | 7971.84 | 0.459 | 3 | 0.335 | 0.010 |
| Sme2.5_02926.1_g00008.1 | 60S ribosomal protein L18a-2-like | 21498.31 | 0.180 | 2 | 0.597 | 0.034 |
| Sme2.5_06171.1_g00007.1 | U6 snRNA-associated Sm-like protein LSm3-like isoform 1 | 21085.98 | 0.106 | 2 | 0.440 | 0.002 |
| Sme2.5_00396.1_g00018.1 | serine/arginine-rich splicing factor RS2Z32-like isoform 1 | 40515.85 | 0.093 | 3 | 0.592 | 0.001 |
| Sme2.5_05641.1_g00001.1 | villin-2-like | 189746.50 | 0.062 | 3 | 0.548 | 0.015 |
| Sme2.5_00018.1_g00025.1 | uncharacterized protein LOC101262625 | 38249.85 | 0.750 | 17 | 0.644 | 0.001 |
| Sme2.5_00048.1_g00007.1 | uncharacterized protein LOC101255979 | 83829.65 | 0.166 | 5 | 0.667 | 0.024 |
| Sme2.5_09245.1_g00002.1 | MAR-binding filament-like protein 1 | 96795.12 | 0.068 | 5 | 0.590 | 0.001 |
| Sme2.5_00925.1_g00001.1 | diaminopimelate decarboxylase 1, chloroplastic-like | 55339.61 | 0.200 | 8 | 0.630 | 0.001 |
| Sme2.5_02018.1_g00002.1 | uncharacterized protein LOC101267115 isoform 1 | 45328.25 | 0.035 | 2 | 0.315 | 0.010 |
| Sme2.5_02308.1_g00006.1 | 40S ribosomal protein S26-2-like | 15000.93 | 0.070 | 1 | 0.513 | 0.003 |
| Sme2.5_05918.1_g00004.1 | ferredoxin-1, chloroplastic-like | 15554.39 | 0.147 | 2 | 0.628 | 0.001 |
| Sme2.5_03276.1_g00004.1 | trypsin proteinase inhibitor precursor | 25387.60 | 0.330 | 5 | 0.164 | 0.001 |
| Sme2.5_02233.1_g00003.1 | ribulose bisphosphate carboxylase/oxygenase (chloroplast) | 7899.84 | 0.278 | 1 | 0.445 | 0.001 |
| Sme2.5_01002.1_g00003.1 | NAD(P)H dehydrogenase B1, mitochondrial-like | 65941.69 | 0.089 | 4 | 0.423 | 0.001 |
| Sme2.5_07601.1_g00002.1 | hypothetical protein VITISV_035070 | 47141.44 | 0.017 | 1 | 0.449 | 0.003 |
| Sme2.5_03301.1_g00002.1 | DEAD-box ATP-dependent RNA helicase 53-like | 68666.22 | 0.090 | 4 | 0.405 | 0.001 |
| Sme2.5_04335.1_g00001.1 | sterol reductase | 66567.09 | 0.211 | 10 | 0.624 | 0.001 |
| Sme2.5_00785.1_g00009.1 | 60S ribosomal protein L36-2-like | 12071.96 | 0.287 | 1 | 0.587 | 0.002 |
| Sme2.5_00066.1_g00007.1 | unknown | 23808.48 | 0.112 | 2 | 0.442 | 0.006 |
| Sme2.5_00276.1_g00004.1 | LOW QUALITY PROTEIN: DNA replication licensing factor mcm2-like | 108486.10 | 0.164 | 13 | 0.506 | 0.001 |
| Sme2.5_00001.1_g00041.1 | 60S ribosomal protein L10 | 23511.46 | 0.215 | 2 | 0.591 | 0.001 |
| Sme2.5_03619.1_g00002.1 | uncharacterized protein LOC101266727 | 109496.30 | 0.056 | 5 | 0.497 | 0.001 |
| Sme2.5_03330.1_g00004.1 | dolichyl-diphosphooligosaccharide--protein glycosyltransferase subunit STT3A-like | 91125.47 | 0.031 | 2 | 0.611 | 0.041 |
| Sme2.5_02208.1_g00002.1 | Eukaryotic translation initiation factor 3 subunit A | 112740.40 | 0.238 | 19 | 0.575 | 0.001 |
| Sme2.5_01800.1_g00006.1 | laccase-4-like | 61692.60 | 0.136 | 5 | 0.452 | 0.001 |
| Sme2.5_01069.1_g00001.1 | chloroplast ferredoxin I | 15915.67 | 0.299 | 3 | 0.615 | 0.016 |
| Sme2.5_01085.1_g00005.1 | cytochrome P450 84A1-like | 59557.42 | 0.042 | 2 | 0.433 | 0.040 |
| Sme2.5_04205.1_g00001.1 | ABC transporter G family member 5-like | 100481.20 | 0.008 | 1 | 0.246 | 0.022 |
| Sme2.5_00115.1_g00006.1 | perakine reductase-like | 38558.09 | 0.087 | 2 | 0.590 | 0.009 |
| Sme2.5_08023.1_g00001.1 | interactor of constitutive active ROPs 3-like isoform 1 | 74822.09 | 0.092 | 5 | 0.622 | 0.003 |
| Sme2.5_00381.1_g00010.1 | ruvB-like 1-like | 49323.92 | 0.080 | 3 | 0.508 | 0.045 |
| Sme2.5_05142.1_g00002.1 | sucrose synthase-like | 91913.37 | 0.308 | 15 | 0.500 | 0.001 |
| Sme2.5_00912.1_g00012.1 | LO4 | 136470.90 | 0.033 | 3 | 0.516 | 0.003 |
| Sme2.5_09062.1_g00002.1 | uncharacterized protein LOC544064 | 74843.78 | 0.613 | 28 | 0.594 | 0.001 |
| Sme2.5_00431.1_g00002.1 | polyribonucleotide nucleotidyltransferase 1, chloroplastic-like | 177616.90 | 0.054 | 7 | 0.657 | 0.011 |
| Sme2.5_00059.1_g00019.1 | splicing factor 3A subunit 3-like | 59928.09 | 0.071 | 3 | 0.628 | 0.046 |
| Sme2.5_04993.1_g00002.1 | chloroplast rubisco activase | 47191.84 | 0.492 | 13 | 0.655 | 0.001 |
| Sme2.5_00310.1_g00016.1 | uncharacterized protein At5g48480-like | 17072.45 | 0.627 | 7 | 0.415 | 0.001 |
| Sme2.5_29276.1_g00001.1 | eukaryotic translation initiation factor 3 subunit J-like | 20024.32 | 0.278 | 4 | 0.645 | 0.001 |
| Sme2.5_06085.1_g00007.1 | storekeeper protein | 44889.66 | 0.306 | 13 | 0.562 | 0.001 |
| Sme2.5_01286.1_g00007.1 | PI-PLC X domain-containing protein At5g67130-like | 44917.86 | 0.117 | 4 | 0.655 | 0.020 |
| Sme2.5_00236.1_g00003.1 | 60S ribosomal protein L8-like | 28436.92 | 0.215 | 2 | 0.610 | 0.002 |
| Sme2.5_00048.1_g00028.1 | uncharacterized protein LOC101250105 | 63267.26 | 0.227 | 12 | 0.498 | 0.001 |
| Sme2.5_01741.1_g00011.1 | putative vesicle-associated membrane protein 726-like | 31320.23 | 0.078 | 1 | 0.646 | 0.022 |
| Sme2.5_06629.1_g00004.1 | uncharacterized protein LOC101256345 isoform 1 | 39649.34 | 0.159 | 4 | 0.530 | 0.001 |
| Sme2.5_00088.1_g00019.1 | 40S ribosomal protein S3a-like | 33114.47 | 0.463 | 5 | 0.492 | 0.001 |
| Sme2.5_24001.1_g00001.1 | putative glutamine synthase 2 | 34376.68 | 0.178 | 2 | 0.605 | 0.010 |
| Sme2.5_00076.1_g00009.1 | 7-dehydrocholesterol reductase-like | 50740.01 | 0.046 | 2 | 0.480 | 0.001 |
| Sme2.5_04309.1_g00005.1 | HMG1/2-like protein-like isoform 2 | 15804.77 | 0.486 | 2 | 0.344 | 0.015 |
| Sme2.5_01730.1_g00004.1 | uncharacterized protein LOC101265655 isoform 1 | 28515.32 | 0.153 | 3 | 0.264 | 0.001 |
| Sme2.5_02418.1_g00011.1 | T-complex protein 1 subunit epsilon-like | 65781.66 | 0.156 | 5 | 0.629 | 0.033 |
| Sme2.5_00529.1_g00002.1 | dnaJ homolog subfamily C member 2-like | 74257.29 | 0.040 | 3 | 0.447 | 0.015 |
| Sme2.5_00132.1_g00008.1 | uncharacterized protein LOC543757 | 116494.20 | 0.101 | 8 | 0.526 | 0.008 |
| Sme2.5_09582.1_g00001.1 | uridine 5'-monophosphate synthase-like | 41168.91 | 0.357 | 10 | 0.619 | 0.001 |
| Sme2.5_01635.1_g00012.1 | proline iminopeptidase-like | 28944.77 | 0.081 | 3 | 0.600 | 0.034 |
| Sme2.5_05872.1_g00003.1 | 26S protease regulatory subunit S10B homolog B-like | 44224.03 | 0.288 | 8 | 0.635 | 0.001 |
| Sme2.5_04909.1_g00002.1 | acid phosphatase 1-like | 20675.12 | 0.146 | 2 | 0.603 | 0.036 |
| Sme2.5_30819.1_g00001.1 | osmotin-like protein precursor | 15399.24 | 0.203 | 2 | 0.394 | 0.004 |
| Sme2.5_05227.1_g00002.1 | probable nucleolar protein 5-2-like | 62915.94 | 0.297 | 12 | 0.562 | 0.001 |
| Sme2.5_06540.1_g00006.1 | ATP synthase CF1 alpha subunit | 19199.71 | 0.161 | 3 | 0.550 | 0.001 |
| Sme2.5_00225.1_g00034.1 | non-specific lipid-transfer protein-like protein At2g13820-like | 19099.32 | 0.059 | 1 | 0.111 | 0.034 |
| Sme2.5_00014.1_g00016.1 | histone H1 | 28785.72 | 0.309 | 7 | 0.163 | 0.001 |
| Sme2.5_08703.1_g00001.1 | 4-coumarate--CoA ligase 1-like | 59744.78 | 0.235 | 11 | 0.561 | 0.001 |
| Sme2.5_01135.1_g00011.1 | putative histone H1/H5 domain family protein | 19517.64 | 0.441 | 7 | 0.383 | 0.001 |
| Sme2.5_02974.1_g00001.1 | high mobility group B protein 1-like isoform 1 | 20285.04 | 0.517 | 6 | 0.370 | 0.001 |
| Sme2.5_06050.1_g00003.1 | 30S ribosomal protein S13, chloroplastic-like | 21416.49 | 0.137 | 3 | 0.529 | 0.011 |
| Sme2.5_00027.1_g00021.1 | DNA replication licensing factor MCM6-like | 99936.15 | 0.106 | 8 | 0.613 | 0.014 |
| Sme2.5_01270.1_g00007.1 | splicing factor 3B subunit 3-like | 133325.10 | 0.092 | 8 | 0.562 | 0.001 |
| Sme2.5_01952.1_g00004.1 | 60S ribosomal protein L5-like | 34877.01 | 0.118 | 3 | 0.542 | 0.001 |
| Sme2.5_02476.1_g00006.1 | WEB family protein At5g16730, chloroplastic-like isoform 1 | 110171.10 | 0.206 | 18 | 0.499 | 0.001 |
| Sme2.5_02852.1_g00002.1 | putative cinnamyl alcohol dehydrogenase | 39574.81 | 0.297 | 8 | 0.663 | 0.001 |
| Sme2.5_03999.1_g00002.1 | zeatin O-glucosyltransferase-like | 51204.60 | 0.047 | 2 | 0.596 | 0.033 |
| Sme2.5_03712.1_g00004.1 | endo-1,4-beta-glucanase precursor | 52259.68 | 0.066 | 3 | 0.574 | 0.024 |
| Sme2.5_00127.1_g00018.1 | putative L24 ribosomal protein | 16708.16 | 0.212 | 1 | 0.348 | 0.001 |
| Sme2.5_02309.1_g00002.1 | pectinesterase/pectinesterase inhibitor 18-like | 59778.20 | 0.236 | 8 | 0.571 | 0.001 |
| Sme2.5_00216.1_g00007.1 | probable glutamate--tRNA ligase, cytoplasmic-like | 88528.72 | 0.129 | 8 | 0.620 | 0.001 |
| Sme2.5_02902.1_g00002.1 | uncharacterized protein LOC101266113 | 52326.86 | 0.063 | 2 | 0.529 | 0.011 |
| Sme2.5_00899.1_g00005.1 | mitochondrial small heat shock protein | 44376.36 | 0.098 | 3 | 0.460 | 0.015 |
| Sme2.5_05345.1_g00001.1 | argonaute1-2, partial | 125052.30 | 0.229 | 17 | 0.569 | 0.001 |
| Sme2.5_05864.1_g00001.1 | fasciclin-like arabinogalactan protein 11-like | 25795.19 | 0.106 | 2 | 0.443 | 0.016 |
| Sme2.5_04458.1_g00001.1 | pathogenesis related protein isoform b1 | 17229.18 | 0.553 | 4 | 0.582 | 0.001 |
| Sme2.5_00983.1_g00002.1 | isoleucine--tRNA ligase, cytoplasmic-like | 106960.70 | 0.084 | 7 | 0.644 | 0.001 |
| Sme2.5_02262.1_g00006.1 | uncharacterized protein LOC101250613 | 31072.98 | 0.445 | 9 | 0.458 | 0.001 |
| Sme2.5_00581.1_g00006.1 | unknown | 44977.24 | 0.406 | 14 | 0.611 | 0.001 |
| Sme2.5_00746.1_g00001.1 | uncharacterized protein LOC101268581 | 69155.50 | 0.226 | 11 | 0.512 | 0.001 |
| Sme2.5_02560.1_g00005.1 | patellin-5-like | 64371.88 | 0.467 | 19 | 0.527 | 0.001 |
| Sme2.5_00798.1_g00007.1 | uncharacterized protein LOC101251433 | 50197.94 | 0.047 | 2 | 0.505 | 0.025 |
| Sme2.5_00179.1_g00007.1 | glyceraldehyde-3-phosphate dehydrogenase B, chloroplastic-like | 48522.01 | 0.416 | 8 | 0.593 | 0.001 |
| Sme2.5_09577.1_g00002.1 | DNA-directed RNA polymerase II 15.1 kDa polypeptide, putative, expressed | 22966.94 | 0.075 | 1 | 0.373 | 0.022 |
| Sme2.5_00264.1_g00023.1 | 40S ribosomal protein S6-like | 28582.65 | 0.321 | 2 | 0.559 | 0.014 |
| Sme2.5_08170.1_g00002.1 | ATP-dependent Clp protease proteolytic subunit-related protein 1, chloroplastic-like | 130904.60 | 0.022 | 2 | 0.631 | 0.049 |
| Sme2.5_00563.1_g00008.1 | uncharacterized protein LOC101256330 | 78158.07 | 0.032 | 2 | 0.574 | 0.007 |
| Sme2.5_00669.1_g00006.1 | geranylgeranyl diphosphate reductase, chloroplastic-like | 51700.47 | 0.274 | 9 | 0.483 | 0.001 |
| Sme2.5_07192.1_g00004.1 | uncharacterized protein LOC101267523 | 195336.30 | 0.049 | 5 | 0.582 | 0.024 |
| Sme2.5_07116.1_g00002.1 | Tubulin beta-1 chain | 46917.05 | 0.438 | 1 | 0.338 | 0.014 |
| Sme2.5_03699.1_g00003.1 | arginine/serine-rich-splicing factor RSP31-like | 33478.97 | 0.117 | 3 | 0.468 | 0.003 |
| Sme2.5_00125.1_g00021.1 | magnesium-protoporphyrin O-methyltransferase-like | 35760.36 | 0.195 | 6 | 0.617 | 0.024 |
| Sme2.5_01346.1_g00008.1 | uncharacterized protein At5g22580-like isoform 1 | 12104.20 | 0.434 | 4 | 0.576 | 0.001 |
| Sme2.5_00006.1_g00009.1 | proteinase inhibitor IIa | 16943.50 | 0.456 | 5 | 0.146 | 0.001 |
| Sme2.5_00039.1_g00019.1 | tubulin alpha chain-like | 48448.84 | 0.548 | 4 | 0.631 | 0.001 |
| Sme2.5_03603.1_g00006.1 | uncharacterized protein LOC101246887 | 61892.76 | 0.099 | 4 | 0.623 | 0.003 |
| Sme2.5_13579.1_g00001.1 | peroxidase 3-like | 34698.50 | 0.312 | 9 | 0.401 | 0.001 |
| Sme2.5_02622.1_g00007.1 | heat shock protein 90-like | 88932.06 | 0.263 | 12 | 0.560 | 0.001 |
| Sme2.5_01754.1_g00007.1 | arginine--tRNA ligase, cytoplasmic-like | 42866.15 | 0.095 | 4 | 0.532 | 0.001 |
| Sme2.5_04411.1_g00001.1 | uncharacterized protein LOC101250539 | 18676.34 | 0.209 | 4 | 0.481 | 0.002 |
| Sme2.5_24166.1_g00001.1 | FACT complex subunit SSRP1-like | 12218.90 | 0.429 | 4 | 0.634 | 0.010 |
| Sme2.5_03336.1_g00005.1 | 29 kDa ribonucleoprotein B, chloroplastic | 32903.16 | 0.224 | 5 | 0.622 | 0.021 |
| Sme2.5_12578.1_g00001.1 | 12S seed storage protein CRU2-like | 38872.12 | 0.301 | 8 | 0.543 | 0.001 |
| Sme2.5_00536.1_g00016.1 | uncharacterized protein LOC101251994 | 124522.00 | 0.075 | 7 | 0.588 | 0.008 |
| Sme2.5_01638.1_g00006.1 | PGR5-like protein 1A, chloroplastic-like | 38798.77 | 0.117 | 3 | 0.649 | 0.042 |
| Sme2.5_03432.1_g00008.1 | 60S ribosomal protein L24-like | 19818.88 | 0.272 | 5 | 0.458 | 0.001 |
| Sme2.5_06875.1_g00002.1 | uridine kinase-like protein 1, chloroplastic-like isoform 2 | 53139.25 | 0.148 | 2 | 0.636 | 0.014 |
| Sme2.5_00406.1_g00012.1 | 60S ribosomal protein L7A-like | 29400.55 | 0.252 | 7 | 0.570 | 0.001 |
| Sme2.5_01015.1_g00005.1 | zinc finger CCCH domain-containing protein 19-like | 89567.24 | 0.056 | 3 | 0.661 | 0.022 |
| Sme2.5_01240.1_g00004.1 | sterol reductase | 66415.78 | 0.388 | 17 | 0.630 | 0.001 |
| Sme2.5_02975.1_g00005.1 | polyribonucleotide nucleotidyltransferase 2, mitochondrial-like | 111130.30 | 0.040 | 4 | 0.628 | 0.046 |
| Sme2.5_24838.1_g00001.1 | methionine sulfoxide reducatase | 9728.64 | 0.429 | 3 | 0.653 | 0.001 |
| Sme2.5_04411.1_g00004.1 | DEAD-box ATP-dependent RNA helicase 37-like isoform 1 | 65204.40 | 0.085 | 4 | 0.432 | 0.001 |
| Sme2.5_02703.1_g00006.1 | zinc finger CCCH domain-containing protein 14-like | 32995.43 | 0.210 | 3 | 0.635 | 0.001 |
| Sme2.5_01462.1_g00018.1 | actin-7-like | 41967.98 | 0.610 | 5 | 0.614 | 0.001 |
| Sme2.5_08172.1_g00001.1 | peptidyl-prolyl cis-trans isomerase FKBP53-like | 55929.66 | 0.128 | 5 | 0.555 | 0.001 |
| Sme2.5_00550.1_g00018.1 | uncharacterized protein LOC101257864 | 19463.89 | 0.041 | 1 | 0.250 | 0.001 |
| Sme2.5_05287.1_g00005.1 | GDP-mannose 3',5'-epimerase | 42899.10 | 0.322 | 4 | 0.482 | 0.001 |
| Sme2.5_04541.1_g00004.1 | uncharacterized protein LOC101250056 | 245610.30 | 0.044 | 3 | 0.630 | 0.049 |
