# Supplementary material for: Comparative proteomic analysis of eggplant (Solanum melongena L.) heterostylous pistil development
Source: PLoS One. 2017 Jun 6;12(6):e0179018. doi: 10.1371/journal.pone.0179018 (PMC5460878; doi:10.1371/journal.pone.0179018)
Supplement: S5 Table — (DOCX) [file pone.0179018.s010.docx]

**Table S5 Downregulated proteins in pistils of S-morph flowers during maturity with a 1.5-fold change compared with developmental stage**

| **Protein_ID** | **Description** | **Mass** | **Coverage** | **Peptide** | **Fold change** | **Qvalue** |
| --- | --- | --- | --- | --- | --- | --- |
| Sme2.5_00225.1_g00030.1 | chloroplast pigment-binding protein CP24 | 27201.09 | 0.349 | 6 | 0.557 | 0.001 |
| Sme2.5_01519.1_g00005.1 | heat shock cognate 70 kDa protein 2-like | 76379.75 | 0.563 | 10 | 0.575 | 0.001 |
| Sme2.5_00179.1_g00004.1 | probable protein phosphatase 2C 27-like | 67361.47 | 0.151 | 8 | 0.467 | 0.001 |
| Sme2.5_02661.1_g00004.1 | UDP-glucose:glycoprotein glucosyltransferase-like | 198307.20 | 0.058 | 8 | 0.544 | 0.003 |
| Sme2.5_00239.1_g00020.1 | U-box domain-containing protein 72-like | 57214.92 | 0.103 | 4 | 0.519 | 0.022 |
| Sme2.5_01338.1_g00002.1 | ADP, ATP carrier protein | 41673.49 | 0.302 | 5 | 0.621 | 0.001 |
| Sme2.5_24492.1_g00002.1 | sulfur | 36086.98 | 0.239 | 6 | 0.545 | 0.031 |
| Sme2.5_00226.1_g00008.1 | deacetylase-like protein | 33633.09 | 0.149 | 5 | 0.385 | 0.002 |
| Sme2.5_01907.1_g00017.1 | peroxisomal (S)-2-hydroxy-acid oxidase GLO5 isoform 1 | 40867.33 | 0.448 | 8 | 0.564 | 0.001 |
| Sme2.5_00406.1_g00017.1 | uncharacterized protein LOC101251618 isoform 2 | 39956.94 | 0.248 | 8 | 0.458 | 0.001 |
| Sme2.5_00427.1_g00011.1 | uncharacterized protein LOC101244815 | 76902.07 | 0.011 | 1 | 0.100 | 0.023 |
| Sme2.5_00280.1_g00001.1 | 40S ribosomal protein S3-3-like | 26376.14 | 0.631 | 12 | 0.394 | 0.001 |
| Sme2.5_05137.1_g00005.1 | ketol-acid reductoisomerase, chloroplastic-like | 63615.31 | 0.159 | 4 | 0.408 | 0.001 |
| Sme2.5_00323.1_g00009.1 | 14-3-3 protein | 45127.58 | 0.187 | 4 | 0.547 | 0.020 |
| Sme2.5_00183.1_g00015.1 | U5 small nuclear ribonucleoprotein 200 kDa helicase-like | 248023.50 | 0.061 | 12 | 0.651 | 0.002 |
| Sme2.5_00747.1_g00009.1 | caffeic acid 3-O-methyltransferase-like | 39886.98 | 0.507 | 12 | 0.642 | 0.001 |
| Sme2.5_00690.1_g00004.1 | FAM10 family protein At4g22670-like | 42402.76 | 0.272 | 6 | 0.565 | 0.001 |
| Sme2.5_04790.1_g00002.1 | PSII 47kDa protein | 78067.33 | 0.020 | 1 | 0.462 | 0.002 |
| Sme2.5_03239.1_g00006.1 | dimethylallyltransferase | 39369.07 | 0.189 | 6 | 0.559 | 0.001 |
| Sme2.5_00080.1_g00004.1 | uncharacterized GPI-anchored protein At1g27950-like | 25539.65 | 0.085 | 2 | 0.308 | 0.007 |
| Sme2.5_00039.1_g00016.1 | KH domain-containing protein At4g18375-like | 73899.44 | 0.085 | 6 | 0.394 | 0.006 |
| Sme2.5_03475.1_g00009.1 | nucleolar protein 56-like | 62323.45 | 0.216 | 8 | 0.366 | 0.019 |
| Sme2.5_00512.1_g00006.1 | ruBisCO large subunit-binding protein subunit alpha, chloroplastic-like | 62339.15 | 0.447 | 17 | 0.491 | 0.001 |
| Sme2.5_03359.1_g00007.1 | uncharacterized protein LOC101268824 | 13780.10 | 0.500 | 3 | 0.558 | 0.001 |
| Sme2.5_01868.1_g00001.1 | ruvB-like 2-like | 52108.84 | 0.070 | 3 | 0.462 | 0.028 |
| Sme2.5_04479.1_g00005.1 | protein TIC 62, chloroplastic-like | 53490.73 | 0.461 | 17 | 0.600 | 0.001 |
| Sme2.5_03425.1_g00011.1 | oligopeptidase A-like | 93233.63 | 0.219 | 15 | 0.623 | 0.001 |
| Sme2.5_00676.1_g00001.1 | 60S ribosomal protein L7-4-like | 27859.18 | 0.396 | 2 | 0.433 | 0.004 |
| Sme2.5_01102.1_g00001.1 | cullin-associated NEDD8-dissociated protein 1-like | 222514.00 | 0.038 | 6 | 0.448 | 0.006 |
| Sme2.5_00106.1_g00004.1 | 40S ribosomal protein SA-like | 32637.27 | 0.358 | 3 | 0.516 | 0.013 |
| Sme2.5_03566.1_g00005.1 | adenylosuccinate lyase-like | 54119.85 | 0.048 | 2 | 0.357 | 0.002 |
| Sme2.5_06815.1_g00001.1 | spermidine synthase | 45811.67 | 0.259 | 8 | 0.469 | 0.001 |
| Sme2.5_10833.1_g00002.1 | transketolase, chloroplastic-like | 80662.78 | 0.374 | 14 | 0.507 | 0.001 |
| Sme2.5_02386.1_g00003.1 | carbonic anhydrase, partial | 35093.85 | 0.208 | 6 | 0.530 | 0.013 |
| Sme2.5_00864.1_g00011.1 | uncharacterized protein LOC101258386 | 92906.82 | 0.052 | 4 | 0.442 | 0.002 |
| Sme2.5_09172.1_g00003.1 | leucine--tRNA ligase, cytoplasmic-like | 115150.70 | 0.184 | 10 | 0.580 | 0.027 |
| Sme2.5_03252.1_g00006.1 | heat shock protein 83-like | 81455.33 | 0.349 | 5 | 0.417 | 0.001 |
| Sme2.5_00034.1_g00013.1 | transcription elongation factor SPT6-like | 186224.40 | 0.031 | 5 | 0.547 | 0.003 |
| Sme2.5_02996.1_g00005.1 | uncharacterized protein LOC101246515 | 104188.80 | 0.084 | 7 | 0.595 | 0.013 |
| Sme2.5_04649.1_g00002.1 | ER membrane protein complex subunit 1-like | 109791.30 | 0.059 | 5 | 0.557 | 0.002 |
| Sme2.5_01489.1_g00008.1 | CASP-like protein RCOM_1206790-like | 19354.34 | 0.155 | 3 | 0.309 | 0.032 |
| Sme2.5_06238.1_g00003.1 | heat shock cognate protein 80 | 84351.99 | 0.418 | 1 | 0.358 | 0.001 |
| Sme2.5_00106.1_g00014.1 | glyceraldehyde-3-phosphate dehydrogenase, cytosolic-like | 38419.97 | 0.573 | 6 | 0.594 | 0.001 |
| Sme2.5_00909.1_g00007.1 | uncharacterized protein LOC101251176 | 89841.66 | 0.140 | 9 | 0.536 | 0.002 |
| Sme2.5_06059.1_g00002.1 | DNA damage-binding protein 1 | 42411.52 | 0.024 | 1 | 0.397 | 0.014 |
| Sme2.5_00488.1_g00017.1 | 40S ribosomal protein S24-2-like isoform 1 | 15970.84 | 0.341 | 2 | 0.547 | 0.003 |
| Sme2.5_05225.1_g00004.1 | asparagine--tRNA ligase, cytoplasmic 1 | 67139.50 | 0.276 | 15 | 0.458 | 0.001 |
| Sme2.5_00750.1_g00006.1 | eugenol synthase 1 | 22610.75 | 0.292 | 5 | 0.615 | 0.001 |
| Sme2.5_05121.1_g00002.1 | 14-3-3 protein | 29333.59 | 0.729 | 7 | 0.586 | 0.001 |
| Sme2.5_03582.1_g00003.1 | proliferation-associated protein 2G4-like | 44958.26 | 0.282 | 7 | 0.574 | 0.001 |
| Sme2.5_09039.1_g00001.1 | DEAD-box ATP-dependent RNA helicase 3, chloroplastic-like | 80942.76 | 0.031 | 2 | 0.323 | 0.041 |
| Sme2.5_13401.1_g00004.1 | predicted protein | 34821.65 | 0.282 | 8 | 0.635 | 0.001 |
| Sme2.5_04919.1_g00006.1 | uncharacterized protein LOC101245729 | 28089.05 | 0.473 | 3 | 0.557 | 0.001 |
| Sme2.5_00193.1_g00008.1 | ATP synthase subunit gamma, mitochondrial-like | 38591.34 | 0.328 | 8 | 0.594 | 0.003 |
| Sme2.5_01559.1_g00002.1 | Histone H1 | 30859.06 | 0.268 | 6 | 0.100 | 0.001 |
| Sme2.5_00883.1_g00003.1 | unknown protein DS12 from 2D-PAGE of leaf, chloroplastic-like | 55425.64 | 0.164 | 8 | 0.528 | 0.003 |
| Sme2.5_01945.1_g00009.1 | phosphoglycerate kinase precursor | 50571.96 | 0.687 | 18 | 0.633 | 0.001 |
| Sme2.5_01448.1_g00008.1 | uncharacterized protein LOC101261442 | 92633.23 | 0.042 | 3 | 0.585 | 0.034 |
| Sme2.5_00650.1_g00013.1 | DNA replication licensing factor mcm4-like | 98128.02 | 0.116 | 9 | 0.546 | 0.024 |
| Sme2.5_01166.1_g00004.1 | tripeptidyl-peptidase 2-like | 25387.48 | 0.217 | 4 | 0.565 | 0.040 |
| Sme2.5_00499.1_g00025.1 | 60S ribosomal protein L6-like | 25441.33 | 0.314 | 5 | 0.320 | 0.001 |
| Sme2.5_09735.1_g00002.1 | DNA-directed RNA polymerases I and III subunit RPAC1-like | 43602.60 | 0.062 | 2 | 0.438 | 0.022 |
| Sme2.5_06238.1_g00001.1 | heat shock protein 90-1 | 81118.22 | 0.424 | 2 | 0.425 | 0.007 |
| Sme2.5_01775.1_g00002.1 | DNA replication licensing factor mcm5-A-like | 82403.40 | 0.091 | 5 | 0.348 | 0.005 |
| Sme2.5_05121.1_g00005.1 | phospho-2-dehydro-3-deoxyheptonate aldolase 2, chloroplastic | 60074.43 | 0.267 | 8 | 0.568 | 0.001 |
| Sme2.5_00502.1_g00011.1 | 14-3-3 protein | 9760.183 | 0.093 | 1 | 0.476 | 0.027 |
| Sme2.5_04224.1_g00007.1 | phospholipase A1-IIgamma-like | 45100.01 | 0.186 | 3 | 0.517 | 0.050 |
| Sme2.5_03348.1_g00003.1 | 50S ribosomal protein L1, chloroplastic-like | 37346.82 | 0.264 | 7 | 0.589 | 0.001 |
| Sme2.5_09577.1_g00003.1 | dihydrolipoyl dehydrogenase-like | 64676.02 | 0.155 | 6 | 0.568 | 0.012 |
| Sme2.5_30393.1_g00001.1 | predicted protein | 9834.22 | 0.182 | 2 | 0.342 | 0.007 |
| Sme2.5_02268.1_g00007.1 | activator of 90 kDa heat shock protein ATPase homolog | 39359.33 | 0.493 | 12 | 0.517 | 0.001 |
| Sme2.5_06540.1_g00005.1 | ATP synthase CF1 alpha chain | 32186.68 | 0.322 | 8 | 0.403 | 0.001 |
| Sme2.5_10126.1_g00002.1 | acyl-[acyl-carrier-protein] desaturase, chloroplastic-like | 49098.11 | 0.187 | 5 | 0.537 | 0.004 |
| Sme2.5_01918.1_g00003.1 | 40S ribosomal protein S15-like | 17241.40 | 0.318 | 3 | 0.511 | 0.002 |
| Sme2.5_03319.1_g00005.1 | probable methionine--tRNA ligase-like | 32377.18 | 0.136 | 4 | 0.477 | 0.027 |
| Sme2.5_03836.1_g00005.1 | ubiquitin extension protein | 17859.54 | 0.391 | 1 | 0.100 | 0.001 |
| Sme2.5_00343.1_g00015.1 | ras-related protein RABC2a-like | 21749.30 | 0.056 | 1 | 0.438 | 0.004 |
| Sme2.5_06059.1_g00001.1 | DNA damage-binding protein 1 | 88031.03 | 0.046 | 3 | 0.518 | 0.013 |
| Sme2.5_03745.1_g00004.1 | ATP synthase gamma chain, chloroplastic-like | 41617.86 | 0.244 | 7 | 0.547 | 0.001 |
| Sme2.5_04984.1_g00003.1 | proteinase inhibitor II | 25168.62 | 0.333 | 5 | 0.289 | 0.001 |
| Sme2.5_00601.1_g00009.1 | translocon-associated protein subunit beta-like | 32086.73 | 0.153 | 3 | 0.624 | 0.023 |
| Sme2.5_00234.1_g00013.1 | calmodulin-related protein isoform 4 | 16875.90 | 0.617 | 1 | 0.614 | 0.009 |
| Sme2.5_05182.1_g00002.1 | Ribulose bisphosphate carboxylase small chain 3, chloroplastic | 20620.23 | 0.544 | 2 | 0.350 | 0.001 |
| Sme2.5_05701.1_g00004.1 | uncharacterized protein LOC101263810 | 75627.96 | 0.018 | 1 | 0.100 | 0.041 |
| Sme2.5_00845.1_g00014.1 | adipocyte plasma membrane-associated protein-like, partial | 12336.21 | 0.091 | 1 | 0.344 | 0.001 |
| Sme2.5_01185.1_g00005.1 | endoplasmin homolog | 92419.47 | 0.373 | 16 | 0.449 | 0.001 |
| Sme2.5_06391.1_g00003.1 | cyclic nucleotide-gated ion channel 1-like | 13069.66 | 0.088 | 1 | 0.100 | 0.020 |
| Sme2.5_00146.1_g00009.1 | UDP-glucuronate decarboxylase 1 | 39063.07 | 0.493 | 3 | 0.521 | 0.009 |
| Sme2.5_00538.1_g00008.1 | 60S ribosomal protein L23a-like | 17457.84 | 0.266 | 2 | 0.455 | 0.023 |
| Sme2.5_00577.1_g00007.1 | N-alpha-acetyltransferase 16, NatA auxiliary subunit-like | 17406.05 | 0.196 | 3 | 0.454 | 0.026 |
| Sme2.5_02104.1_g00006.1 | uncharacterized protein LOC101260453 | 21674.39 | 0.111 | 1 | 0.100 | 0.001 |
| Sme2.5_02262.1_g00005.1 | uncharacterized protein LOC101250613 | 46238.49 | 0.253 | 9 | 0.415 | 0.001 |
| Sme2.5_08458.1_g00002.1 | 60S ribosomal protein L26-1-like | 16707.14 | 0.212 | 1 | 0.317 | 0.038 |
| Sme2.5_00186.1_g00019.1 | eukaryotic translation initiation factor 3 subunit E-like | 51311.00 | 0.249 | 8 | 0.638 | 0.028 |
| Sme2.5_16412.1_g00001.1 | Kunitz-type enzyme inhibitor S9C11 | 21996.90 | 0.365 | 6 | 0.589 | 0.001 |
| Sme2.5_01494.1_g00003.1 | 60S ribosomal protein L19-2-like | 24901.74 | 0.299 | 2 | 0.335 | 0.023 |
| Sme2.5_00026.1_g00018.1 | ribosomal protein PETRP-like | 15530.16 | 0.422 | 3 | 0.506 | 0.001 |
| Sme2.5_00043.1_g00024.1 | heat shock protein 70 isoform 3 | 71726.28 | 0.608 | 9 | 0.527 | 0.001 |
| Sme2.5_02700.1_g00001.1 | elongation factor 2-like isoform 1 | 94848.84 | 0.444 | 27 | 0.488 | 0.001 |
| Sme2.5_00001.1_g00048.1 | cinnamic acid 4-hydroxylase | 58331.85 | 0.244 | 12 | 0.530 | 0.001 |
| Sme2.5_11618.1_g00003.1 | fasciclin-like arabinogalactan protein 2-like | 42408.78 | 0.086 | 3 | 0.515 | 0.005 |
| Sme2.5_09948.1_g00002.1 | 60S ribosomal protein L27a-3-like | 16468.95 | 0.236 | 2 | 0.559 | 0.019 |
| Sme2.5_00079.1_g00001.1 | uncharacterized protein LOC101244722 | 21014.85 | 0.219 | 3 | 0.500 | 0.002 |
| Sme2.5_01379.1_g00003.1 | ISPH protein | 50489.55 | 0.223 | 8 | 0.599 | 0.007 |
| Sme2.5_02364.1_g00010.1 | H/ACA ribonucleoprotein complex subunit 4-like | 66778.28 | 0.229 | 10 | 0.235 | 0.001 |
| Sme2.5_03718.1_g00001.1 | elongation factor 1-gamma 2-like isoform 2 | 47324.01 | 0.413 | 14 | 0.386 | 0.001 |
| Sme2.5_00414.1_g00004.1 | argininosuccinate synthase, chloroplastic-like | 55725.94 | 0.210 | 9 | 0.433 | 0.001 |
| Sme2.5_01509.1_g00012.1 | uncharacterized protein LOC101262922 | 76868.59 | 0.012 | 1 | 0.100 | 0.003 |
| Sme2.5_02268.1_g00004.1 | 60S ribosomal protein L21-2-like | 18730.14 | 0.354 | 1 | 0.448 | 0.021 |
| Sme2.5_03682.1_g00010.1 | uncharacterized protein LOC101301900 | 81117.02 | 0.103 | 5 | 0.504 | 0.015 |
| Sme2.5_01731.1_g00002.1 | phospholipase A1-II 1-like isoform 1 | 45216.21 | 0.722 | 13 | 0.375 | 0.001 |
| Sme2.5_00813.1_g00006.1 | serrate RNA effector molecule-like | 92548.35 | 0.072 | 5 | 0.351 | 0.001 |
| Sme2.5_00021.1_g00020.1 | proliferating cell nuclear antigen | 21157.42 | 0.640 | 3 | 0.543 | 0.007 |
| Sme2.5_00020.1_g00016.1 | dioxygenase | 38246.35 | 0.367 | 10 | 0.442 | 0.001 |
| Sme2.5_00216.1_g00021.1 | eukaryotic translation initiation factor 3 subunit C-like | 82578.41 | 0.108 | 7 | 0.479 | 0.002 |
| Sme2.5_02777.1_g00010.1 | NAD-dependent malic enzyme 62 kDa isoform, mitochondrial-like | 120868.60 | 0.106 | 8 | 0.608 | 0.014 |
| Sme2.5_00026.1_g00001.1 | glyceraldehyde 3-phosphate dehydrogenase | 38609.81 | 0.521 | 5 | 0.305 | 0.001 |
| Sme2.5_00842.1_g00002.1 | 40S ribosomal protein S15a | 14819.88 | 0.562 | 7 | 0.536 | 0.001 |
| Sme2.5_00036.1_g00006.1 | carbamoyl-phosphate synthase small chain-like | 48434.07 | 0.168 | 6 | 0.657 | 0.001 |
| Sme2.5_00142.1_g00011.1 | pullulanase 1, chloroplastic-like | 109731.80 | 0.007 | 1 | 0.233 | 0.010 |
| Sme2.5_00165.1_g00018.1 | checkpoint serine/threonine-protein kinase BUB1-like | 99139.29 | 0.098 | 4 | 0.464 | 0.003 |
| Sme2.5_12551.1_g00002.1 | cycloartenol synthase | 34947.44 | 0.132 | 3 | 0.339 | 0.001 |
| Sme2.5_01441.1_g00007.1 | 40S ribosomal protein S6-like | 29900.16 | 0.336 | 3 | 0.381 | 0.005 |
| Sme2.5_01862.1_g00006.1 | heat shock 70 kDa protein 15-like | 90100.78 | 0.448 | 11 | 0.652 | 0.001 |
| Sme2.5_03522.1_g00004.1 | squamous cell carcinoma antigen recognized by T-cells 3-like | 103965.40 | 0.059 | 6 | 0.479 | 0.027 |
| Sme2.5_01746.1_g00006.1 | putative cytochrome P450 | 58934.91 | 0.137 | 6 | 0.132 | 0.024 |
| Sme2.5_02951.1_g00008.1 | 14-3-3 protein | 31651.94 | 0.345 | 3 | 0.562 | 0.004 |
| Sme2.5_09579.1_g00003.1 | polyadenylate-binding protein 4-like isoform 1 | 43673.53 | 0.231 | 7 | 0.521 | 0.001 |
| Sme2.5_13136.1_g00002.1 | unknown | 32007.07 | 0.051 | 2 | 0.124 | 0.025 |
| Sme2.5_00016.1_g00027.1 | cell division cycle protein 48 homolog | 85883.50 | 0.355 | 2 | 0.510 | 0.015 |
| Sme2.5_00745.1_g00010.1 | 14-3-3-like protein 16R | 47299.16 | 0.377 | 3 | 0.415 | 0.001 |
| Sme2.5_02055.1_g00002.1 | probable fructose-bisphosphate aldolase 2, chloroplastic-like | 42266.70 | 0.345 | 8 | 0.512 | 0.001 |
| Sme2.5_01027.1_g00011.1 | Hop-interacting protein THI016 | 65690.37 | 0.177 | 7 | 0.388 | 0.002 |
| Sme2.5_06588.1_g00001.1 | lysine--tRNA ligase-like | 86927.57 | 0.232 | 16 | 0.502 | 0.001 |
| Sme2.5_00401.1_g00012.1 | signal recognition particle 43 kDa protein, chloroplastic-like | 41525.89 | 0.051 | 2 | 0.339 | 0.049 |
| Sme2.5_30574.1_g00001.1 | early nodulin-like protein 1-like | 19644.13 | 0.350 | 6 | 0.592 | 0.001 |
| Sme2.5_00025.1_g00027.1 | uncharacterized protein At5g49945-like | 55852.32 | 0.130 | 5 | 0.531 | 0.006 |
| Sme2.5_13120.1_g00001.1 | aspartic proteinase nepenthesin-2-like | 99505.24 | 0.060 | 3 | 0.484 | 0.009 |
| Sme2.5_01339.1_g00003.1 | glutamyl-tRNA(Gln) amidotransferase subunit B, chloroplastic/mitochondrial-like | 79674.82 | 0.065 | 4 | 0.457 | 0.026 |
| Sme2.5_00733.1_g00012.1 | uncharacterized protein LOC101249817 | 189000.30 | 0.111 | 14 | 0.631 | 0.001 |
| Sme2.5_01489.1_g00004.1 | AGO1A | 118852.20 | 0.156 | 8 | 0.414 | 0.001 |
| Sme2.5_01835.1_g00009.1 | ATP-dependent Clp protease proteolytic subunit 4, chloroplastic-like | 29362.88 | 0.302 | 6 | 0.568 | 0.015 |
| Sme2.5_10884.1_g00005.1 | 5-methyltetrahydropteroyltriglutamate--homocysteine methyltransferase-like | 53736.51 | 0.368 | 9 | 0.591 | 0.001 |
| Sme2.5_00048.1_g00024.1 | 28 kDa ribonucleoprotein, chloroplastic-like | 34786.98 | 0.256 | 6 | 0.647 | 0.001 |
| Sme2.5_00018.1_g00002.1 | cinnamoyl-CoA reductase | 30224.31 | 0.114 | 3 | 0.427 | 0.046 |
| Sme2.5_02902.1_g00005.1 | glyceraldehyde-3-phosphate dehydrogenase A, chloroplastic-like | 43062.47 | 0.471 | 11 | 0.410 | 0.001 |
| Sme2.5_00444.1_g00001.1 | uncharacterized protein LOC101252108 | 240985.30 | 0.078 | 12 | 0.552 | 0.001 |
| Sme2.5_01239.1_g00002.1 | ATP-citrate synthase alpha chain protein 3-like | 46908.01 | 0.182 | 4 | 0.465 | 0.006 |
| Sme2.5_05238.1_g00005.1 | 60S ribosomal protein L6-like | 25493.32 | 0.306 | 5 | 0.324 | 0.002 |
| Sme2.5_00495.1_g00010.1 | fructose-1,6-bisphosphatase, chloroplastic-like | 44554.25 | 0.206 | 6 | 0.584 | 0.006 |
| Sme2.5_00065.1_g00022.1 | 1,2-dihydroxy-3-keto-5-methylthiopentene dioxygenase 2-like | 23574.49 | 0.250 | 3 | 0.363 | 0.006 |
| Sme2.5_00551.1_g00006.1 | 14-3-3 protein 4 | 34114.17 | 0.298 | 3 | 0.480 | 0.008 |
| Sme2.5_00121.1_g00010.1 | uncharacterized protein LOC101255522 isoform 1 | 60610.32 | 0.147 | 7 | 0.438 | 0.001 |
| Sme2.5_00022.1_g00018.1 | uncharacterized protein LOC101244470 | 73935.80 | 0.098 | 6 | 0.412 | 0.003 |
| Sme2.5_00287.1_g00013.1 | ankyrin-like protein | 55657.50 | 0.053 | 3 | 0.456 | 0.048 |
| Sme2.5_00238.1_g00011.1 | glutamine--tRNA ligase-like | 91542.12 | 0.144 | 9 | 0.553 | 0.009 |
| Sme2.5_05621.1_g00013.1 | transketolase, chloroplastic-like | 79995.29 | 0.418 | 15 | 0.550 | 0.001 |
| Sme2.5_17229.1_g00001.1 | putative F-box protein PP2-B12-like | 20279.09 | 0.609 | 8 | 0.394 | 0.001 |
| Sme2.5_00789.1_g00007.1 | 50S ribosomal protein L5, chloroplastic-like | 29669.63 | 0.256 | 6 | 0.578 | 0.026 |
| Sme2.5_00519.1_g00012.1 | signal recognition particle 54 kDa protein, chloroplastic-like isoform 1 | 62192.63 | 0.138 | 6 | 0.447 | 0.001 |
| Sme2.5_00079.1_g00013.1 | deoxyuridine 5'-triphosphate nucleotidohydrolase | 18221.51 | 0.616 | 7 | 0.587 | 0.001 |
| Sme2.5_00729.1_g00014.1 | uncharacterized protein LOC101245270 | 47471.27 | 0.060 | 2 | 0.308 | 0.039 |
| Sme2.5_02232.1_g00007.1 | biotin carboxylase 1, chloroplastic-like | 63253.22 | 0.283 | 3 | 0.440 | 0.003 |
| Sme2.5_04544.1_g00001.1 | expansin9 precursor | 28711.31 | 0.035 | 1 | 0.497 | 0.021 |
| Sme2.5_00942.1_g00003.1 | ribosomal protein S14-like protein | 16373.64 | 0.367 | 1 | 0.307 | 0.002 |
| Sme2.5_00145.1_g00001.1 | peroxisomal (S)-2-hydroxy-acid oxidase GLO1 | 47887.72 | 0.450 | 10 | 0.527 | 0.001 |
| Sme2.5_00146.1_g00020.1 | very-long-chain enoyl-CoA reductase-like | 56723.31 | 0.036 | 2 | 0.336 | 0.013 |
| Sme2.5_00310.1_g00014.1 | 60S ribosomal protein L27a-3-like | 16430.90 | 0.372 | 4 | 0.529 | 0.001 |
| Sme2.5_00843.1_g00004.1 | ATPase family AAA domain-containing protein 3-B-like | 70103.04 | 0.087 | 5 | 0.448 | 0.006 |
| Sme2.5_00925.1_g00002.1 | bifunctional dihydrofolate reductase-thymidylate synthase-like | 56301.24 | 0.174 | 6 | 0.523 | 0.020 |
| Sme2.5_00065.1_g00007.1 | aspartic proteinase oryzasin-1-like | 50820.41 | 0.239 | 8 | 0.499 | 0.001 |
| Sme2.5_06364.1_g00001.1 | 60S ribosomal protein L34-like isoform 1 | 15618.94 | 0.294 | 2 | 0.450 | 0.007 |
| Sme2.5_00372.1_g00003.1 | ruBisCO large subunit-binding protein subunit beta, chloroplastic-like | 66207.71 | 0.320 | 10 | 0.578 | 0.001 |
| Sme2.5_00756.1_g00008.1 | uncharacterized protein OsI_027940-like | 20407.82 | 0.240 | 3 | 0.506 | 0.037 |
| Sme2.5_02308.1_g00009.1 | tetraketide alpha-pyrone reductase 1-like | 30386.58 | 0.343 | 7 | 0.403 | 0.001 |
| Sme2.5_05137.1_g00003.1 | 14-3-3 protein 8 | 29706.61 | 0.308 | 5 | 0.395 | 0.001 |
| Sme2.5_02324.1_g00006.1 | eukaryotic translation initiation factor 1A-like | 22957.35 | 0.105 | 2 | 0.648 | 0.013 |
| Sme2.5_03963.1_g00009.1 | uncharacterized protein LOC101268824 | 65720.73 | 0.165 | 5 | 0.637 | 0.009 |
| Sme2.5_00019.1_g00025.1 | carbamoyl-phosphate synthase large chain-like | 112971.70 | 0.104 | 8 | 0.626 | 0.018 |
| Sme2.5_00368.1_g00010.1 | 4-coumarate--CoA ligase-like 1-like | 61656.04 | 0.215 | 9 | 0.154 | 0.001 |
| Sme2.5_05117.1_g00001.1 | threonine--tRNA ligase, mitochondrial-like | 153879.30 | 0.112 | 13 | 0.575 | 0.001 |
| Sme2.5_03352.1_g00006.1 | nuclear-pore anchor-like | 231317.80 | 0.053 | 9 | 0.554 | 0.001 |
| Sme2.5_12406.1_g00002.1 | GMP synthase [glutamine-hydrolyzing]-like | 59773.79 | 0.133 | 6 | 0.487 | 0.020 |
| Sme2.5_01143.1_g00009.1 | patellin-3-like | 54889.16 | 0.256 | 9 | 0.615 | 0.005 |
| Sme2.5_00423.1_g00019.1 | ATP-dependent zinc metalloprotease FTSH 2, chloroplastic-like | 62404.73 | 0.258 | 7 | 0.660 | 0.029 |
| Sme2.5_01441.1_g00006.1 | chloroplast polyphenol oxidase precursor | 57950.85 | 0.454 | 14 | 0.570 | 0.001 |
| Sme2.5_30033.1_g00001.1 | unknown | 22168.66 | 0.345 | 2 | 0.393 | 0.002 |
| Sme2.5_02518.1_g00007.1 | putative arginine/serine-rich protein-like | 47269.20 | 0.106 | 5 | 0.424 | 0.001 |
| Sme2.5_06466.1_g00001.1 | methionine aminopeptidase 2B-like isoform 1 | 48733.52 | 0.139 | 5 | 0.641 | 0.012 |
| Sme2.5_01183.1_g00023.1 | heat shock cognate 70 kDa protein-like | 71480.22 | 0.547 | 7 | 0.655 | 0.002 |
| Sme2.5_02961.1_g00005.1 | 4,5-DOPA dioxygenase extradiol-like protein-like | 78296.94 | 0.135 | 6 | 0.517 | 0.025 |
| Sme2.5_02370.1_g00005.1 | xanthine dehydrogenase 1-like | 149730.60 | 0.267 | 24 | 0.474 | 0.001 |
| Sme2.5_01164.1_g00001.1 | omega-hydroxypalmitate O-feruloyl transferase-like | 42735.54 | 0.222 | 7 | 0.405 | 0.001 |
| Sme2.5_00456.1_g00007.1 | uncharacterized protein LOC101259218 | 55622.05 | 0.703 | 21 | 0.609 | 0.001 |
| Sme2.5_00016.1_g00017.1 | transmembrane 9 superfamily member 4-like | 76660.04 | 0.123 | 5 | 0.516 | 0.001 |
| Sme2.5_00142.1_g00014.1 | malate dehydrogenase | 48914.95 | 0.228 | 8 | 0.599 | 0.001 |
| Sme2.5_26015.1_g00001.1 | molecular chaperone Hsp90-1 | 76046.63 | 0.425 | 5 | 0.399 | 0.001 |
| Sme2.5_00281.1_g00013.1 | ribosomal protein L3 | 44797.00 | 0.393 | 8 | 0.174 | 0.001 |
| Sme2.5_02142.1_g00005.1 | uncharacterized protein LOC101259467 | 51818.75 | 0.402 | 13 | 0.476 | 0.001 |
| Sme2.5_01887.1_g00007.1 | uncharacterized protein LOC101255308 | 143027.90 | 0.033 | 4 | 0.585 | 0.007 |
| Sme2.5_05872.1_g00005.1 | SUMO-activating enzyme subunit 2-like | 53644.02 | 0.111 | 5 | 0.620 | 0.007 |
| Sme2.5_00066.1_g00013.1 | glycine-rich protein precursor | 15485.18 | 0.144 | 1 | 0.580 | 0.001 |
| Sme2.5_08303.1_g00002.1 | phospholipase D alpha 1-like | 92791.53 | 0.122 | 7 | 0.446 | 0.001 |
| Sme2.5_00889.1_g00001.1 | Aqp2 protein | 30783.84 | 0.204 | 2 | 0.422 | 0.023 |
| Sme2.5_00606.1_g00004.1 | dehydrin-like protein | 24181.16 | 0.563 | 8 | 0.403 | 0.001 |
| Sme2.5_05072.1_g00005.1 | phosphoribulokinase, chloroplastic-like | 47521.18 | 0.297 | 10 | 0.491 | 0.001 |
| Sme2.5_08818.1_g00004.1 | serine/arginine-rich splicing factor RSZ21A-like | 29554.90 | 0.190 | 5 | 0.480 | 0.013 |
| Sme2.5_04102.1_g00020.1 | 60S ribosomal protein L8 | 29811.72 | 0.207 | 2 | 0.424 | 0.007 |
| Sme2.5_00230.1_g00002.1 | long chain acyl-CoA synthetase 4-like | 78035.80 | 0.106 | 7 | 0.627 | 0.002 |
| Sme2.5_29964.1_g00001.1 | staphylococcal nuclease domain-containing protein 1-like | 37244.14 | 0.439 | 10 | 0.553 | 0.001 |
| Sme2.5_00099.1_g00017.1 | hypothetical protein 111O18.18 | 64794.04 | 0.121 | 6 | 0.386 | 0.001 |
| Sme2.5_01411.1_g00008.1 | phosphatidylinositide phosphatase SAC1-like | 127406.90 | 0.097 | 7 | 0.439 | 0.001 |
| Sme2.5_00163.1_g00002.1 | protein argonaute 4-like | 105854.50 | 0.264 | 14 | 0.443 | 0.001 |
| Sme2.5_01629.1_g00004.1 | actin-related protein 7-like isoform 1 | 54601.27 | 0.107 | 2 | 0.361 | 0.004 |
| Sme2.5_03623.1_g00010.1 | probable aquaporin PIP-type pTOM75-like | 30971.93 | 0.188 | 1 | 0.628 | 0.001 |
| Sme2.5_00789.1_g00009.1 | valine--tRNA ligase-like | 134613.10 | 0.077 | 7 | 0.179 | 0.002 |
| Sme2.5_00048.1_g00027.1 | indole-3-glycerol phosphate synthase, chloroplastic-like isoform 1 | 39726.97 | 0.249 | 8 | 0.514 | 0.002 |
| Sme2.5_00430.1_g00008.1 | uncharacterized protein LOC101265516 | 71811.72 | 0.014 | 1 | 0.100 | 0.030 |
| Sme2.5_00696.1_g00013.1 | UDP-glucose glucosyltransferase | 55911.77 | 0.245 | 10 | 0.433 | 0.001 |
| Sme2.5_00846.1_g00008.1 | eukaryotic initiation factor 4A-3-like | 45045.43 | 0.232 | 8 | 0.438 | 0.001 |
| Sme2.5_02777.1_g00009.1 | hypothetical protein VITISV_022809 | 181671.50 | 0.049 | 6 | 0.643 | 0.001 |
| Sme2.5_00886.1_g00015.1 | uncharacterized protein LOC101253888 | 54752.73 | 0.094 | 4 | 0.563 | 0.033 |
| Sme2.5_01946.1_g00002.1 | T-complex protein 1 subunit eta-like | 62759.46 | 0.326 | 14 | 0.600 | 0.001 |
| Sme2.5_06553.1_g00002.1 | reticulon-like protein B1-like | 32649.49 | 0.052 | 1 | 0.493 | 0.036 |
| Sme2.5_00034.1_g00009.1 | splicing factor 3B subunit 1-like | 88052.37 | 0.122 | 7 | 0.599 | 0.002 |
| Sme2.5_00108.1_g00014.1 | ly200 protein | 15727.59 | 0.135 | 1 | 0.263 | 0.016 |
| Sme2.5_00346.1_g00019.1 | chalcone synthase-like | 44007.60 | 0.295 | 8 | 0.261 | 0.016 |
| Sme2.5_06072.1_g00001.1 | uncharacterized protein LOC101251468 | 23331.86 | 0.164 | 3 | 0.601 | 0.007 |
| Sme2.5_00133.1_g00001.1 | T-complex protein 1 subunit beta-like | 112967.10 | 0.144 | 11 | 0.623 | 0.001 |
| Sme2.5_03095.1_g00004.1 | eukaryotic peptide chain release factor subunit 1-3-like | 49130.92 | 0.103 | 4 | 0.464 | 0.005 |
| Sme2.5_05119.1_g00003.1 | 26S protease regulatory subunit 7 homolog A-like | 56179.62 | 0.364 | 15 | 0.634 | 0.001 |
| Sme2.5_00502.1_g00014.1 | phosphoenolpyruvate carboxylase, housekeeping isozyme-like | 64132.31 | 0.349 | 7 | 0.524 | 0.001 |
| Sme2.5_00140.1_g00009.1 | ArcA2 protein-like | 81059.13 | 0.177 | 4 | 0.648 | 0.006 |
| Sme2.5_06588.1_g00003.1 | 60S ribosomal protein L9-1-like | 37687.69 | 0.473 | 6 | 0.453 | 0.001 |
| Sme2.5_14763.1_g00003.1 | probable fructose-bisphosphate aldolase 2, chloroplastic-like | 42149.69 | 0.356 | 5 | 0.577 | 0.014 |
| Sme2.5_00290.1_g00001.1 | cellulose synthase-like protein G1-like | 79250.81 | 0.122 | 7 | 0.424 | 0.006 |
| Sme2.5_06426.1_g00002.1 | peroxidase 40-like isoform 1 | 64534.19 | 0.065 | 3 | 0.516 | 0.014 |
| Sme2.5_00388.1_g00009.1 | LRR receptor-like serine/threonine-protein kinase FLS2-like | 45788.49 | 0.363 | 10 | 0.100 | 0.001 |
| Sme2.5_22373.1_g00002.1 | proteinase inhibitor type-2 CEVI57 precursor | 24139.72 | 0.114 | 2 | 0.581 | 0.007 |
| Sme2.5_04651.1_g00003.1 | mediator-associated protein 1-like | 43289.34 | 0.324 | 9 | 0.336 | 0.001 |
| Sme2.5_05511.1_g00003.1 | probable phenylalanine--tRNA ligase beta subunit-like | 90610.24 | 0.084 | 4 | 0.511 | 0.041 |
| Sme2.5_06032.1_g00002.1 | uncharacterized protein LOC101261541 | 54226.99 | 0.086 | 4 | 0.598 | 0.003 |
| Sme2.5_04937.1_g00006.1 | AGO4A | 98736.61 | 0.069 | 3 | 0.595 | 0.009 |
| Sme2.5_05102.1_g00004.1 | eukaryotic translation initiation factor 3 subunit I-like | 38053.38 | 0.248 | 8 | 0.567 | 0.001 |
| Sme2.5_06932.1_g00003.1 | ferredoxin--NADP reductase, leaf isozyme, chloroplastic-like | 40377.22 | 0.543 | 14 | 0.664 | 0.001 |
| Sme2.5_02223.1_g00002.1 | selenoprotein H-like | 17212.35 | 0.196 | 3 | 0.354 | 0.004 |
| Sme2.5_02588.1_g00006.1 | serine/threonine-protein phosphatase 6 regulatory ankyrin repeat subunit C-like | 25347.86 | 0.105 | 2 | 0.579 | 0.030 |
| Sme2.5_00347.1_g00010.1 | glutamine synthetase | 39317.67 | 0.272 | 3 | 0.590 | 0.009 |
| Sme2.5_04997.1_g00001.1 | ribulose-1,5-bisphosphate carboxylase/oxygenase large subunit | 14436.36 | 0.057 | 1 | 0.100 | 0.013 |
| Sme2.5_01494.1_g00004.1 | isoamylase isoform 3 | 72413.38 | 0.073 | 4 | 0.591 | 0.035 |
| Sme2.5_02984.1_g00002.1 | polyubiquitin-like | 44423.86 | 0.016 | 1 | 0.100 | 0.020 |
| Sme2.5_11776.1_g00001.1 | polyphenol oxidase F, chloroplastic-like | 62932.33 | 0.093 | 2 | 0.471 | 0.002 |
| Sme2.5_01260.1_g00004.1 | elongation factor Tu, mitochondrial-like | 49373.48 | 0.356 | 4 | 0.570 | 0.001 |
| Sme2.5_00059.1_g00010.1 | SPM1 protein | 46124.21 | 0.087 | 3 | 0.562 | 0.024 |
| Sme2.5_00565.1_g00014.1 | 2-Cys peroxiredoxin BAS1-like, chloroplastic-like | 29560.17 | 0.330 | 4 | 0.536 | 0.001 |
| Sme2.5_03583.1_g00003.1 | uncharacterized protein LOC101252226 | 146888.60 | 0.165 | 15 | 0.507 | 0.001 |
| Sme2.5_12240.1_g00001.1 | uncharacterized protein LOC101306013 | 114772.30 | 0.028 | 2 | 0.100 | 0.005 |
| Sme2.5_00210.1_g00012.1 | bifunctional aspartokinase/homoserine dehydrogenase, chloroplastic-like | 20631.45 | 0.384 | 5 | 0.510 | 0.010 |
| Sme2.5_06770.1_g00003.1 | putative nuclear matrix constituent protein 1-like protein-like | 134056.10 | 0.071 | 8 | 0.503 | 0.025 |
| Sme2.5_00118.1_g00007.1 | alpha-glucosidase | 61176.01 | 0.117 | 5 | 0.515 | 0.049 |
| Sme2.5_00292.1_g00003.1 | Histone deacetylase HDT1 | 30081.73 | 0.263 | 4 | 0.553 | 0.002 |
| Sme2.5_01561.1_g00008.1 | exportin-1 isoform 3 | 173375.60 | 0.067 | 9 | 0.618 | 0.018 |
| Sme2.5_00439.1_g00003.1 | GTP-binding nuclear protein Ran2 | 26467.31 | 0.188 | 4 | 0.597 | 0.001 |
| Sme2.5_02187.1_g00008.1 | eukaryotic translation initiation factor 3 subunit D-like isoform 1 | 65608.69 | 0.078 | 4 | 0.574 | 0.021 |
| Sme2.5_08706.1_g00002.1 | uncharacterized protein LOC101252351 | 96719.25 | 0.117 | 7 | 0.540 | 0.002 |
| Sme2.5_02552.1_g00001.1 | 26S proteasome non-ATPase regulatory subunit 13-like | 71809.01 | 0.078 | 4 | 0.466 | 0.010 |
| Sme2.5_04436.1_g00001.1 | LOW QUALITY PROTEIN: DNA-directed RNA polymerase E subunit 1 | 240993.00 | 0.053 | 9 | 0.450 | 0.002 |
| Sme2.5_04401.1_g00002.1 | protein HOTHEAD-like | 54469.65 | 0.216 | 8 | 0.397 | 0.001 |
| Sme2.5_04375.1_g00005.1 | tubulin beta-1 chain-like | 48482.96 | 0.409 | 1 | 0.267 | 0.014 |
| Sme2.5_00508.1_g00013.1 | pentatricopeptide repeat-containing protein At3g49240-like | 74221.21 | 0.129 | 6 | 0.519 | 0.032 |
| Sme2.5_01136.1_g00003.1 | ADP/ATP translocator-like | 50164.96 | 0.248 | 5 | 0.446 | 0.001 |
| Sme2.5_00381.1_g00011.1 | GcpE | 82442.27 | 0.262 | 16 | 0.536 | 0.001 |
| Sme2.5_08713.1_g00004.1 | UPA21 | 39601.35 | 0.249 | 8 | 0.658 | 0.001 |
| Sme2.5_05198.1_g00003.1 | AGO5 | 112537.10 | 0.113 | 8 | 0.501 | 0.001 |
| Sme2.5_01301.1_g00006.1 | pyruvate kinase, cytosolic isozyme-like | 63548.29 | 0.295 | 5 | 0.541 | 0.001 |
| Sme2.5_00121.1_g00009.1 | AGO4A | 106570.80 | 0.249 | 8 | 0.541 | 0.001 |
| Sme2.5_01374.1_g00009.1 | cytoplasmic ribosomal protein S13-like | 18548.42 | 0.472 | 7 | 0.482 | 0.001 |
| Sme2.5_05245.1_g00003.1 | protein TOC75-3, chloroplastic-like | 92842.33 | 0.183 | 11 | 0.527 | 0.001 |
| Sme2.5_01772.1_g00001.1 | glucan endo-1,3-beta-glucosidase-like protein 3-like | 19333.22 | 0.048 | 1 | 0.538 | 0.011 |
| Sme2.5_10132.1_g00002.1 | uncharacterized protein LOC101248248 | 76130.89 | 0.162 | 8 | 0.661 | 0.011 |
| Sme2.5_03722.1_g00005.1 | phosphoenolpyruvate carboxylase | 106816.00 | 0.288 | 4 | 0.468 | 0.027 |
| Sme2.5_00298.1_g00003.1 | protein SET-like isoform 1 | 33363.96 | 0.307 | 5 | 0.478 | 0.001 |
| Sme2.5_01754.1_g00006.1 | unknown | 8199.97 | 0.474 | 3 | 0.513 | 0.044 |
| Sme2.5_01602.1_g00010.1 | importin subunit alpha-1a-like | 38485.92 | 0.314 | 7 | 0.597 | 0.004 |
| Sme2.5_04107.1_g00007.1 | patellin-4-like | 54566.21 | 0.168 | 6 | 0.490 | 0.042 |
| Sme2.5_02516.1_g00012.1 | mitochondrial Rho GTPase 1-like | 76114.26 | 0.155 | 8 | 0.591 | 0.001 |
| Sme2.5_05323.1_g00006.1 | glutamate-1-semialdehyde 2,1-aminomutase, chloroplastic | 20132.37 | 0.139 | 2 | 0.380 | 0.001 |
| Sme2.5_06504.1_g00002.1 | alpha-1,4-glucan-protein synthase [UDP-forming]-like | 40360.98 | 0.100 | 1 | 0.100 | 0.029 |
| Sme2.5_04854.1_g00009.1 | pentatricopeptide repeat-containing protein At1g80270, mitochondrial | 71261.17 | 0.088 | 5 | 0.582 | 0.037 |
| Sme2.5_00115.1_g00014.1 | annexin D4-like | 53570.82 | 0.288 | 13 | 0.602 | 0.002 |
| Sme2.5_06732.1_g00001.1 | probable histone H2A variant 3-like | 20463.06 | 0.183 | 1 | 0.553 | 0.006 |
| Sme2.5_03640.1_g00006.1 | 40S ribosomal protein S17-like | 16221.71 | 0.556 | 3 | 0.536 | 0.001 |
| Sme2.5_05205.1_g00002.1 | photosystem II CP43 protein | 41639.18 | 0.046 | 2 | 0.551 | 0.001 |
| Sme2.5_05426.1_g00003.1 | 33 kDa ribonucleoprotein, chloroplastic-like | 32195.51 | 0.201 | 4 | 0.459 | 0.008 |
| Sme2.5_00001.1_g00046.1 | eukaryotic translation initiation factor 2 beta subunit-like | 30172.24 | 0.272 | 5 | 0.542 | 0.001 |
| Sme2.5_00423.1_g00017.1 | chloroplast protease | 12613.26 | 0.574 | 6 | 0.602 | 0.001 |
| Sme2.5_00171.1_g00011.1 | endoplasmin homolog | 89746.27 | 0.425 | 8 | 0.458 | 0.001 |
| Sme2.5_02964.1_g00002.1 | 40S ribosomal protein S4-like | 29946.29 | 0.428 | 6 | 0.557 | 0.001 |
| Sme2.5_01235.1_g00007.1 | chlorophyll a-b binding protein CP29.2, chloroplastic-like | 31230.17 | 0.361 | 7 | 0.452 | 0.001 |
| Sme2.5_00594.1_g00001.1 | 60S ribosomal protein L10 | 24698.94 | 0.250 | 3 | 0.214 | 0.001 |
| Sme2.5_01772.1_g00005.1 | 26S protease regulatory subunit 8 homolog A-like | 49304.71 | 0.405 | 12 | 0.501 | 0.001 |
| Sme2.5_14955.1_g00001.1 | uncharacterized protein LOC101263689 | 91161.18 | 0.046 | 1 | 0.347 | 0.007 |
| Sme2.5_00064.1_g00002.1 | T-complex protein 1 subunit zeta-like | 62244.22 | 0.254 | 5 | 0.407 | 0.001 |
| Sme2.5_00226.1_g00031.1 | 40S ribosomal protein S23-like | 15190.35 | 0.228 | 3 | 0.387 | 0.001 |
| Sme2.5_00007.1_g00010.1 | chaperonin CPN60-like 2, mitochondrial-like | 125882.60 | 0.117 | 9 | 0.660 | 0.021 |
| Sme2.5_00983.1_g00004.1 | transcription initiation factor IIF subunit alpha-like | 59736.05 | 0.078 | 3 | 0.470 | 0.002 |
| Sme2.5_10376.1_g00001.1 | acetate--CoA ligase ACS, chloroplastic/glyoxysomal-like | 85055.92 | 0.029 | 2 | 0.582 | 0.048 |
| Sme2.5_00584.1_g00004.1 | hypothetical protein VITISV_032012 | 41224.09 | 0.024 | 1 | 0.493 | 0.009 |
| Sme2.5_00151.1_g00009.1 | unnamed protein product | 12388.67 | 0.142 | 2 | 0.100 | 0.001 |
| Sme2.5_00219.1_g00014.1 | 26S proteasome non-ATPase regulatory subunit 11-like | 47318.02 | 0.251 | 9 | 0.509 | 0.001 |
| Sme2.5_13692.1_g00001.1 | ribulose-1,5-bisphosphate carboxylase/oxygenase large subunit | 12406.36 | 0.072 | 1 | 0.100 | 0.016 |
| Sme2.5_01918.1_g00005.1 | apyrase-like | 47431.48 | 0.302 | 10 | 0.572 | 0.001 |
| Sme2.5_00276.1_g00013.1 | glycine--tRNA ligase 1, mitochondrial-like | 81659.71 | 0.185 | 7 | 0.543 | 0.001 |
| Sme2.5_02811.1_g00002.1 | uncharacterized protein LOC101245558 | 113730.80 | 0.171 | 12 | 0.522 | 0.001 |
| Sme2.5_05178.1_g00004.1 | KH domain-containing protein At4g18375-like | 71269.78 | 0.108 | 5 | 0.544 | 0.013 |
| Sme2.5_15649.1_g00003.1 | unknown | 7971.84 | 0.459 | 3 | 0.508 | 0.010 |
| Sme2.5_02926.1_g00008.1 | 60S ribosomal protein L18a-2-like | 21498.31 | 0.180 | 2 | 0.510 | 0.049 |
| Sme2.5_00584.1_g00002.1 | 30S ribosomal protein S31, chloroplastic-like | 11783.15 | 0.232 | 3 | 0.432 | 0.003 |
| Sme2.5_08916.1_g00003.1 | probable glutathione S-transferase-like | 26830.52 | 0.220 | 1 | 0.469 | 0.029 |
| Sme2.5_00054.1_g00021.1 | ribosomal protein L25-like protein | 17274.62 | 0.333 | 3 | 0.483 | 0.017 |
| Sme2.5_05641.1_g00001.1 | villin-2-like | 189746.50 | 0.062 | 3 | 0.441 | 0.023 |
| Sme2.5_00018.1_g00025.1 | uncharacterized protein LOC101262625 | 38249.85 | 0.750 | 17 | 0.488 | 0.001 |
| Sme2.5_00085.1_g00025.1 | histone-lysine N-methyltransferase, H3 lysine-9 specific SUVH1-like | 149821.50 | 0.067 | 7 | 0.591 | 0.047 |
| Sme2.5_00625.1_g00009.1 | adenosylhomocysteinase-like isoform 1 | 98737.21 | 0.282 | 9 | 0.628 | 0.001 |
| Sme2.5_09068.1_g00004.1 | Late blight resistance protein, putative | 47162.57 | 0.012 | 1 | 0.579 | 0.037 |
| Sme2.5_00007.1_g00003.1 | WD-40 repeat-containing protein MSI4-like | 62118.68 | 0.123 | 5 | 0.594 | 0.003 |
| Sme2.5_01284.1_g00014.1 | phosphoenolpyruvate carboxykinase | 73914.05 | 0.245 | 6 | 0.541 | 0.001 |
| Sme2.5_09245.1_g00002.1 | MAR-binding filament-like protein 1 | 96795.12 | 0.068 | 5 | 0.636 | 0.005 |
| Sme2.5_01435.1_g00010.1 | serine/threonine-protein phosphatase 2A 65 kDa regulatory subunit A beta isoform-like | 71141.62 | 0.213 | 7 | 0.527 | 0.001 |
| Sme2.5_00925.1_g00001.1 | diaminopimelate decarboxylase 1, chloroplastic-like | 55339.61 | 0.200 | 8 | 0.549 | 0.001 |
| Sme2.5_04403.1_g00002.1 | ras-related protein RABC1-like isoform 1 | 21063.66 | 0.150 | 2 | 0.443 | 0.024 |
| Sme2.5_02018.1_g00002.1 | uncharacterized protein LOC101267115 isoform 1 | 45328.25 | 0.035 | 2 | 0.245 | 0.014 |
| Sme2.5_00043.1_g00017.1 | 2-oxoglutarate dehydrogenase, mitochondrial-like | 112591.8 | 0.158 | 12 | 0.596 | 0.001 |
| Sme2.5_02308.1_g00006.1 | 40S ribosomal protein S26-2-like | 15000.93 | 0.070 | 1 | 0.199 | 0.001 |
| Sme2.5_10667.1_g00002.1 | 40S ribosomal protein S2-2-like | 30294.27 | 0.453 | 9 | 0.487 | 0.001 |
| Sme2.5_06793.1_g00001.1 | uncharacterized protein LOC101250077 | 123333.40 | 0.162 | 14 | 0.538 | 0.002 |
| Sme2.5_02933.1_g00009.1 | chlorophyll a-b binding protein CP26, chloroplastic-like isoform 1 | 29763.51 | 0.384 | 8 | 0.623 | 0.001 |
| Sme2.5_00007.1_g00021.1 | glycylpeptide N-tetradecanoyltransferase 1-like | 49987.44 | 0.125 | 5 | 0.547 | 0.012 |
| Sme2.5_03276.1_g00004.1 | trypsin proteinase inhibitor precursor | 25387.60 | 0.330 | 5 | 0.259 | 0.001 |
| Sme2.5_02233.1_g00003.1 | ribulose bisphosphate carboxylase/oxygenase (chloroplast) | 7899.84 | 0.278 | 1 | 0.311 | 0.001 |
| Sme2.5_09935.1_g00001.1 | ribosome biogenesis regulatory protein homolog | 61521.38 | 0.040 | 2 | 0.491 | 0.009 |
| Sme2.5_02618.1_g00004.1 | brefeldin A resistance protein-like | 61025.70 | 0.420 | 16 | 0.571 | 0.001 |
| Sme2.5_00555.1_g00013.1 | uncharacterized protein LOC101265350 | 41860.93 | 0.248 | 6 | 0.610 | 0.027 |
| Sme2.5_09540.1_g00002.1 | mRNA binding protein precursor | 44031.38 | 0.275 | 8 | 0.649 | 0.002 |
| Sme2.5_00125.1_g00003.1 | calnexin-like protein precursor | 61569.78 | 0.571 | 23 | 0.383 | 0.001 |
| Sme2.5_02351.1_g00007.1 | chlorophyll a/b binding protein | 28353.21 | 0.366 | 2 | 0.491 | 0.008 |
| Sme2.5_00113.1_g00016.1 | nuclear RNA binding protein-like | 39488.42 | 0.421 | 11 | 0.506 | 0.001 |
| Sme2.5_01832.1_g00003.1 | Photosystem II 10 kDa polypeptide, chloroplastic | 14402.49 | 0.058 | 1 | 0.502 | 0.003 |
| Sme2.5_03301.1_g00002.1 | DEAD-box ATP-dependent RNA helicase 53-like | 68666.22 | 0.090 | 4 | 0.316 | 0.002 |
| Sme2.5_04520.1_g00002.1 | dolichyl-diphosphooligosaccharide--protein glycosyltransferase 48 kDa subunit-like | 49038.00 | 0.284 | 10 | 0.520 | 0.001 |
| Sme2.5_07159.1_g00004.1 | heat shock protein 83-like isoform 1 | 81197.36 | 0.320 | 2 | 0.416 | 0.001 |
| Sme2.5_08959.1_g00002.1 | ATP synthase subunit d, mitochondrial-like | 19807.05 | 0.494 | 8 | 0.650 | 0.001 |
| Sme2.5_08766.1_g00002.1 | uncharacterized protein LOC101260160 | 35480.52 | 0.330 | 8 | 0.647 | 0.001 |
| Sme2.5_00003.1_g00021.1 | protein RCC2-like | 57920.46 | 0.145 | 5 | 0.619 | 0.009 |
| Sme2.5_04335.1_g00001.1 | sterol reductase | 66567.09 | 0.211 | 10 | 0.514 | 0.002 |
| Sme2.5_00785.1_g00009.1 | 60S ribosomal protein L36-2-like | 12071.96 | 0.287 | 1 | 0.412 | 0.001 |
| Sme2.5_01889.1_g00006.1 | Impa1 | 61189.46 | 0.123 | 5 | 0.540 | 0.029 |
| Sme2.5_00276.1_g00004.1 | LOW QUALITY PROTEIN: DNA replication licensing factor mcm2-like | 108486.10 | 0.164 | 13 | 0.507 | 0.001 |
| Sme2.5_00341.1_g00012.1 | 26S proteasome non-ATPase regulatory subunit 1-like | 106576.00 | 0.246 | 16 | 0.555 | 0.001 |
| Sme2.5_00001.1_g00041.1 | 60S ribosomal protein L10 | 23511.46 | 0.215 | 2 | 0.373 | 0.001 |
| Sme2.5_00183.1_g00014.1 | proliferation-associated protein 2G4-like | 43133.41 | 0.238 | 5 | 0.396 | 0.001 |
| Sme2.5_00417.1_g00015.1 | adenine phosphoribosyltransferase 1, chloroplastic-like | 29751.83 | 0.573 | 11 | 0.606 | 0.001 |
| Sme2.5_07918.1_g00003.1 | glyceraldehyde-3-phosphate dehydrogenase B, chloroplastic-like | 50893.24 | 0.362 | 7 | 0.582 | 0.001 |
| Sme2.5_03619.1_g00002.1 | uncharacterized protein LOC101266727 | 109496.30 | 0.056 | 5 | 0.350 | 0.001 |
| Sme2.5_00940.1_g00015.1 | 40S ribosomal protein S16-like | 22450.26 | 0.266 | 2 | 0.542 | 0.016 |
| Sme2.5_03330.1_g00004.1 | dolichyl-diphosphooligosaccharide--protein glycosyltransferase subunit STT3A-like | 91125.47 | 0.031 | 2 | 0.446 | 0.046 |
| Sme2.5_02208.1_g00002.1 | Eukaryotic translation initiation factor 3 subunit A | 112740.40 | 0.238 | 19 | 0.454 | 0.001 |
| Sme2.5_04663.1_g00001.1 | putative pyruvate dehydrogenase E1 alpha subunit | 49287.75 | 0.211 | 7 | 0.608 | 0.002 |
| Sme2.5_09157.1_g00002.1 | peptidyl-prolyl cis-trans isomerase FKBP62-like | 151824.70 | 0.069 | 8 | 0.461 | 0.001 |
| Sme2.5_01800.1_g00006.1 | laccase-4-like | 61692.60 | 0.136 | 5 | 0.552 | 0.007 |
| Sme2.5_06569.1_g00005.1 | methionine gamma-lyase-like | 49470.42 | 0.287 | 10 | 0.633 | 0.001 |
| Sme2.5_01085.1_g00005.1 | cytochrome P450 84A1-like | 59557.42 | 0.042 | 2 | 0.230 | 0.011 |
| Sme2.5_07025.1_g00003.1 | histidine--tRNA ligase-like | 100715.40 | 0.132 | 10 | 0.552 | 0.010 |
| Sme2.5_01292.1_g00003.1 | histone H2B.2 | 15801.79 | 0.379 | 1 | 0.528 | 0.009 |
| Sme2.5_00423.1_g00008.1 | ribosomal protein L11-like protein | 20931.98 | 0.326 | 6 | 0.506 | 0.001 |
| Sme2.5_00115.1_g00006.1 | perakine reductase-like | 38558.09 | 0.087 | 2 | 0.593 | 0.043 |
| Sme2.5_33651.1_g00001.1 | chloroplast polyphenol oxidase precursor | 18337.44 | 0.748 | 8 | 0.460 | 0.001 |
| Sme2.5_00041.1_g00008.1 | eukaryotic translation initiation factor isoform 4G-1-like | 81168.93 | 0.065 | 3 | 0.511 | 0.045 |
| Sme2.5_27724.1_g00001.1 | uncharacterized protein LOC101252010 | 22926.35 | 0.206 | 3 | 0.639 | 0.001 |
| Sme2.5_05142.1_g00002.1 | sucrose synthase-like | 91913.37 | 0.308 | 15 | 0.166 | 0.001 |
| Sme2.5_06192.1_g00002.1 | uncharacterized protein LOC101258515 | 65000.24 | 0.145 | 8 | 0.532 | 0.001 |
| Sme2.5_13003.1_g00001.1 | phosphoenolpyruvate carboxylase, housekeeping isozyme-like | 47493.19 | 0.279 | 6 | 0.580 | 0.001 |
| Sme2.5_09062.1_g00002.1 | uncharacterized protein LOC544064 | 74843.78 | 0.613 | 28 | 0.469 | 0.001 |
| Sme2.5_00431.1_g00002.1 | polyribonucleotide nucleotidyltransferase 1, chloroplastic-like | 177616.90 | 0.054 | 7 | 0.429 | 0.001 |
| Sme2.5_03977.1_g00002.1 | rab GDP dissociation inhibitor alpha-like | 73012.12 | 0.256 | 4 | 0.466 | 0.001 |
| Sme2.5_00061.1_g00024.1 | uncharacterized protein LOC101249339 | 66931.78 | 0.160 | 7 | 0.605 | 0.002 |
| Sme2.5_03454.1_g00001.1 | probable sarcosine oxidase-like | 45726.07 | 0.169 | 6 | 0.455 | 0.014 |
| Sme2.5_01189.1_g00017.1 | dolichyl-diphosphooligosaccharide--protein glycosyltransferase subunit 2-like isoform 2 | 79801.09 | 0.182 | 11 | 0.631 | 0.001 |
| Sme2.5_04993.1_g00002.1 | chloroplast rubisco activase | 47191.84 | 0.492 | 13 | 0.627 | 0.001 |
| Sme2.5_00310.1_g00016.1 | uncharacterized protein At5g48480-like | 17072.45 | 0.627 | 7 | 0.344 | 0.001 |
| Sme2.5_29276.1_g00001.1 | eukaryotic translation initiation factor 3 subunit J-like | 20024.32 | 0.278 | 4 | 0.517 | 0.003 |
| Sme2.5_06085.1_g00007.1 | storekeeper protein | 44889.66 | 0.306 | 13 | 0.462 | 0.001 |
| Sme2.5_00502.1_g00010.1 | ketol-acid reductoisomerase, chloroplastic-like | 64064.53 | 0.283 | 9 | 0.612 | 0.001 |
| Sme2.5_03810.1_g00005.1 | 60S ribosomal protein L10a-1-like | 29122.57 | 0.119 | 3 | 0.560 | 0.007 |
| Sme2.5_05713.1_g00002.1 | Arginine/serine-rich splicing factor RSP41, putative | 46864.26 | 0.170 | 4 | 0.487 | 0.045 |
| Sme2.5_00236.1_g00003.1 | 60S ribosomal protein L8-like | 28436.92 | 0.215 | 2 | 0.486 | 0.005 |
| Sme2.5_06344.1_g00002.1 | pyruvate kinase isozyme A, chloroplastic-like | 68220.81 | 0.164 | 8 | 0.512 | 0.001 |
| Sme2.5_00048.1_g00028.1 | uncharacterized protein LOC101250105 | 63267.26 | 0.227 | 12 | 0.127 | 0.001 |
| Sme2.5_00720.1_g00006.1 | 30S ribosomal protein S5, chloroplastic-like | 33435.26 | 0.226 | 6 | 0.620 | 0.003 |
| Sme2.5_00043.1_g00021.1 | hypothetical protein ZEAMMB73_313798 | 11384.39 | 0.573 | 4 | 0.618 | 0.001 |
| Sme2.5_01741.1_g00011.1 | putative vesicle-associated membrane protein 726-like | 31320.23 | 0.078 | 1 | 0.439 | 0.007 |
| Sme2.5_04368.1_g00003.1 | ras-related protein RABE1a-like | 22618.37 | 0.254 | 4 | 0.546 | 0.010 |
| Sme2.5_29856.1_g00001.1 | T-complex protein 1 subunit alpha-like | 13692.23 | 0.427 | 4 | 0.456 | 0.028 |
| Sme2.5_06629.1_g00004.1 | uncharacterized protein LOC101256345 isoform 1 | 39649.34 | 0.159 | 4 | 0.611 | 0.002 |
| Sme2.5_00743.1_g00010.1 | ferredoxin-dependent glutamate synthase 1, chloroplastic-like | 42977.59 | 0.372 | 10 | 0.561 | 0.001 |
| Sme2.5_00088.1_g00019.1 | 40S ribosomal protein S3a-like | 33114.47 | 0.463 | 5 | 0.155 | 0.009 |
| Sme2.5_01027.1_g00013.1 | trigger factor-like protein TIG-like | 59789.79 | 0.309 | 12 | 0.654 | 0.016 |
| Sme2.5_14363.1_g00002.1 | riboflavin synthase-like | 31004.32 | 0.238 | 5 | 0.560 | 0.014 |
| Sme2.5_04309.1_g00005.1 | HMG1/2-like protein-like isoform 2 | 15804.77 | 0.486 | 2 | 0.100 | 0.013 |
| Sme2.5_00654.1_g00017.1 | dolichyl-diphosphooligosaccharide--protein glycosyltransferase subunit STT3B-like | 84423.59 | 0.066 | 4 | 0.562 | 0.025 |
| Sme2.5_03537.1_g00010.1 | chlorophyll a-b binding protein 4, chloroplastic | 25115.50 | 0.376 | 4 | 0.540 | 0.001 |
| Sme2.5_02418.1_g00011.1 | T-complex protein 1 subunit epsilon-like | 65781.66 | 0.156 | 5 | 0.441 | 0.030 |
| Sme2.5_04194.1_g00001.1 | GDSL esterase/lipase At1g29670-like | 36355.02 | 0.064 | 2 | 0.507 | 0.010 |
| Sme2.5_00132.1_g00008.1 | uncharacterized protein LOC543757 | 116494.20 | 0.101 | 8 | 0.386 | 0.045 |
| Sme2.5_09582.1_g00001.1 | uridine 5'-monophosphate synthase-like | 41168.91 | 0.357 | 10 | 0.442 | 0.001 |
| Sme2.5_01166.1_g00006.1 | tripeptidyl peptidase II, putative | 108060.80 | 0.193 | 13 | 0.532 | 0.001 |
| Sme2.5_01635.1_g00012.1 | proline iminopeptidase-like | 28944.77 | 0.081 | 3 | 0.283 | 0.009 |
| Sme2.5_18191.1_g00001.1 | RNA and export factor-binding protein 2-like isoform 2 | 22548.52 | 0.312 | 5 | 0.563 | 0.003 |
| Sme2.5_01055.1_g00009.1 | uncharacterized protein LOC101245871 | 36444.68 | 0.126 | 4 | 0.506 | 0.009 |
| Sme2.5_03353.1_g00001.1 | probable pre-mRNA-splicing factor ATP-dependent RNA helicase-like | 80881.40 | 0.061 | 3 | 0.353 | 0.027 |
| Sme2.5_00018.1_g00030.1 | phospholipase PLDa1 | 92548.15 | 0.157 | 9 | 0.614 | 0.001 |
| Sme2.5_05872.1_g00003.1 | 26S protease regulatory subunit S10B homolog B-like | 44224.03 | 0.288 | 8 | 0.418 | 0.001 |
| Sme2.5_02035.1_g00006.1 | 40S ribosomal protein S7-like protein | 22081.90 | 0.455 | 5 | 0.478 | 0.001 |
| Sme2.5_05227.1_g00002.1 | probable nucleolar protein 5-2-like | 62915.94 | 0.297 | 12 | 0.501 | 0.001 |
| Sme2.5_06540.1_g00006.1 | ATP synthase CF1 alpha subunit | 19199.71 | 0.161 | 3 | 0.407 | 0.001 |
| Sme2.5_09570.1_g00001.1 | ribosomal protein L7 | 28153.51 | 0.401 | 2 | 0.358 | 0.006 |
| Sme2.5_00225.1_g00034.1 | non-specific lipid-transfer protein-like protein At2g13820-like | 19099.32 | 0.059 | 1 | 0.100 | 0.013 |
| Sme2.5_03480.1_g00004.1 | T-complex protein 1 subunit gamma-like | 89532.08 | 0.232 | 16 | 0.636 | 0.001 |
| Sme2.5_08394.1_g00002.1 | CAAX prenyl protease 1 homolog | 48647.75 | 0.130 | 5 | 0.576 | 0.038 |
| Sme2.5_06988.1_g00002.1 | basic leucine zipper and W2 domain-containing protein 2-like | 47299.91 | 0.136 | 5 | 0.636 | 0.013 |
| Sme2.5_04335.1_g00005.1 | photosystem I subunit III precursor (chloroplast) | 25136.31 | 0.171 | 3 | 0.604 | 0.007 |
| Sme2.5_00014.1_g00016.1 | histone H1 | 28785.72 | 0.309 | 7 | 0.100 | 0.001 |
| Sme2.5_21543.1_g00001.1 | uncharacterized protein ycf39-like isoform 2 | 43404.47 | 0.149 | 6 | 0.663 | 0.013 |
| Sme2.5_08703.1_g00001.1 | 4-coumarate--CoA ligase 1-like | 59744.78 | 0.235 | 11 | 0.401 | 0.001 |
| Sme2.5_00832.1_g00002.1 | Photosystem II 22 kDa protein, chloroplastic | 29391.89 | 0.374 | 8 | 0.591 | 0.001 |
| Sme2.5_16965.1_g00001.1 | d-3-phosphoglycerate dehydrogenase, chloroplastic-like | 67367.26 | 0.336 | 13 | 0.596 | 0.001 |
| Sme2.5_01135.1_g00011.1 | putative histone H1/H5 domain family protein | 19517.64 | 0.441 | 7 | 0.358 | 0.001 |
| Sme2.5_02974.1_g00001.1 | high mobility group B protein 1-like isoform 1 | 20285.04 | 0.517 | 6 | 0.265 | 0.002 |
| Sme2.5_06050.1_g00003.1 | 30S ribosomal protein S13, chloroplastic-like | 21416.49 | 0.137 | 3 | 0.313 | 0.023 |
| Sme2.5_00027.1_g00021.1 | DNA replication licensing factor MCM6-like | 99936.15 | 0.106 | 8 | 0.519 | 0.003 |
| Sme2.5_00479.1_g00005.1 | eukaryotic translation initiation factor 3 subunit B-like | 82662.56 | 0.287 | 15 | 0.583 | 0.001 |
| Sme2.5_07446.1_g00003.1 | ribulose-1,5-bisphosphate carboxylase/oxygenase large subunit | 21375.91 | 0.117 | 1 | 0.442 | 0.01 |
| Sme2.5_01270.1_g00007.1 | splicing factor 3B subunit 3-like | 133325.10 | 0.092 | 8 | 0.452 | 0.003 |
| Sme2.5_01952.1_g00004.1 | 60S ribosomal protein L5-like | 34877.01 | 0.118 | 3 | 0.202 | 0.002 |
| Sme2.5_00175.1_g00014.1 | NADP-dependent D-sorbitol-6-phosphate dehydrogenase | 35087.98 | 0.181 | 5 | 0.660 | 0.001 |
| Sme2.5_00094.1_g00007.1 | oxysterol-binding protein-related protein 3A-like | 59755.85 | 0.127 | 5 | 0.528 | 0.003 |
| Sme2.5_02476.1_g00006.1 | WEB family protein At5g16730, chloroplastic-like isoform 1 | 110171.10 | 0.206 | 18 | 0.421 | 0.001 |
| Sme2.5_00025.1_g00010.1 | 60S ribosomal protein L3-like | 46132.53 | 0.251 | 3 | 0.528 | 0.047 |
| Sme2.5_03231.1_g00008.1 | Ribulose bisphosphate carboxylase small chain 8B, chloroplastic | 20647.18 | 0.494 | 1 | 0.347 | 0.004 |
| Sme2.5_02852.1_g00002.1 | putative cinnamyl alcohol dehydrogenase | 39574.81 | 0.297 | 8 | 0.633 | 0.001 |
| Sme2.5_01695.1_g00009.1 | zeatin O-glucosyltransferase-like | 52605.84 | 0.385 | 14 | 0.469 | 0.001 |
| Sme2.5_04260.1_g00006.1 | heterodimeric geranylgeranyl pyrophosphate synthase small subunit, chloroplastic-like isoform 1 | 34534.52 | 0.166 | 4 | 0.550 | 0.002 |
| Sme2.5_05063.1_g00001.1 | polygalacturonase QRT3-like | 104455.70 | 0.198 | 15 | 0.485 | 0.001 |
| Sme2.5_04721.1_g00004.1 | Putative gag-pol polyprotein, identical | 73041.68 | 0.034 | 1 | 0.100 | 0.047 |
| Sme2.5_02393.1_g00005.1 | zeatin O-xylosyltransferase-like | 61266.97 | 0.011 | 1 | 0.291 | 0.028 |
| Sme2.5_00216.1_g00007.1 | probable glutamate--tRNA ligase, cytoplasmic-like | 88528.72 | 0.129 | 8 | 0.427 | 0.001 |
| Sme2.5_01305.1_g00001.1 | annexin p34 | 49002.33 | 0.311 | 12 | 0.608 | 0.001 |
| Sme2.5_01323.1_g00007.1 | hypothetical protein ARALYDRAFT_484358 | 37452.59 | 0.147 | 4 | 0.474 | 0.032 |
| Sme2.5_09582.1_g00003.1 | eukaryotic translation initiation factor 2 subunit alpha-like | 32869.00 | 0.115 | 3 | 0.498 | 0.044 |
| Sme2.5_01102.1_g00002.1 | survival of motor neuron-related-splicing factor 30-like | 33202.64 | 0.084 | 2 | 0.497 | 0.038 |
| Sme2.5_04364.1_g00002.1 | 14-3-3 protein | 28989.43 | 0.602 | 4 | 0.646 | 0.027 |
| Sme2.5_00297.1_g00018.1 | FAM10 family protein At4g22670-like | 46449.96 | 0.468 | 13 | 0.563 | 0.001 |
| Sme2.5_04299.1_g00002.1 | Phosphoglucomutase, chloroplastic | 62346.38 | 0.089 | 4 | 0.659 | 0.029 |
| Sme2.5_05356.1_g00009.1 | puromycin-sensitive aminopeptidase-like | 99773.54 | 0.316 | 23 | 0.562 | 0.001 |
| Sme2.5_05345.1_g00001.1 | argonaute1-2, partial | 125052.30 | 0.229 | 17 | 0.373 | 0.001 |
| Sme2.5_05864.1_g00001.1 | fasciclin-like arabinogalactan protein 11-like | 25795.19 | 0.106 | 2 | 0.507 | 0.033 |
| Sme2.5_04458.1_g00001.1 | pathogenesis related protein isoform b1 | 17229.18 | 0.553 | 4 | 0.654 | 0.002 |
| Sme2.5_02450.1_g00007.1 | FtsH-like protein precursor | 76338.59 | 0.380 | 19 | 0.613 | 0.001 |
| Sme2.5_02903.1_g00005.1 | probable 6-phosphogluconolactonase 4, chloroplastic-like | 45861.00 | 0.141 | 5 | 0.639 | 0.020 |
| Sme2.5_00983.1_g00002.1 | isoleucine--tRNA ligase, cytoplasmic-like | 106960.70 | 0.084 | 7 | 0.425 | 0.003 |
| Sme2.5_01216.1_g00019.1 | d-3-phosphoglycerate dehydrogenase, chloroplastic-like | 60103.59 | 0.420 | 13 | 0.515 | 0.001 |
| Sme2.5_02262.1_g00006.1 | uncharacterized protein LOC101250613 | 31072.98 | 0.445 | 9 | 0.116 | 0.001 |
| Sme2.5_00581.1_g00006.1 | unknown | 44977.24 | 0.406 | 14 | 0.426 | 0.001 |
| Sme2.5_00746.1_g00001.1 | uncharacterized protein LOC101268581 | 69155.50 | 0.226 | 11 | 0.471 | 0.001 |
| Sme2.5_05147.1_g00002.1 | Linoleate 13S-lipoxygenase 2-1, chloroplastic | 91894.85 | 0.368 | 18 | 0.647 | 0.001 |
| Sme2.5_01435.1_g00004.1 | uncharacterized protein LOC101246632 | 134632.8 | 0.034 | 3 | 0.550 | 0.008 |
| Sme2.5_02560.1_g00005.1 | patellin-5-like | 64371.88 | 0.467 | 19 | 0.531 | 0.001 |
| Sme2.5_00551.1_g00001.1 | heat shock cognate 70 kDa protein 2-like | 75789.43 | 0.485 | 5 | 0.596 | 0.001 |
| Sme2.5_01531.1_g00010.1 | calcium-transporting ATPase 4, endoplasmic reticulum-type-like | 108128.90 | 0.118 | 10 | 0.650 | 0.001 |
| Sme2.5_00057.1_g00019.1 | glyoxisomal malate dehydrogenase | 38050.94 | 0.361 | 9 | 0.624 | 0.001 |
| Sme2.5_00827.1_g00002.1 | 26S proteasome regulatory subunit 4 homolog A-like | 89146.56 | 0.284 | 17 | 0.543 | 0.001 |
| Sme2.5_00798.1_g00007.1 | uncharacterized protein LOC101251433 | 50197.94 | 0.047 | 2 | 0.331 | 0.004 |
| Sme2.5_00954.1_g00007.1 | 40S ribosomal protein S20-1-like | 13839.47 | 0.154 | 2 | 0.601 | 0.001 |
| Sme2.5_00179.1_g00007.1 | glyceraldehyde-3-phosphate dehydrogenase B, chloroplastic-like | 48522.01 | 0.416 | 8 | 0.484 | 0.001 |
| Sme2.5_02324.1_g00008.1 | uncharacterized protein At4g01150, chloroplastic-like isoform 2 | 17951.45 | 0.124 | 2 | 0.574 | 0.002 |
| Sme2.5_04732.1_g00004.1 | thaumatin-like protein-like | 27264.07 | 0.040 | 1 | 0.370 | 0.044 |
| Sme2.5_06878.1_g00001.1 | cytochrome P450 704C1-like | 59002.25 | 0.060 | 3 | 0.291 | 0.007 |
| Sme2.5_00264.1_g00023.1 | 40S ribosomal protein S6-like | 28582.65 | 0.321 | 2 | 0.386 | 0.012 |
| Sme2.5_00125.1_g00014.1 | glycine--tRNA ligase 1, mitochondrial-like | 77075.01 | 0.221 | 9 | 0.557 | 0.001 |
| Sme2.5_09540.1_g00003.1 | Ran GTPase-activating protein 1 | 66004.99 | 0.147 | 7 | 0.605 | 0.002 |
| Sme2.5_00047.1_g00027.1 | exportin-2-like | 110347.20 | 0.072 | 6 | 0.590 | 0.001 |
| Sme2.5_00563.1_g00008.1 | uncharacterized protein LOC101256330 | 78158.07 | 0.032 | 2 | 0.517 | 0.038 |
| Sme2.5_00669.1_g00006.1 | geranylgeranyl diphosphate reductase, chloroplastic-like | 51700.47 | 0.274 | 9 | 0.261 | 0.001 |
| Sme2.5_07116.1_g00002.1 | Tubulin beta-1 chain | 46917.05 | 0.438 | 1 | 0.327 | 0.040 |
| Sme2.5_27818.1_g00001.1 | HMG-Y-related protein A-like | 18675.49 | 0.515 | 7 | 0.636 | 0.001 |
| Sme2.5_02159.1_g00009.1 | 3-isopropylmalate dehydratase-like | 55919.85 | 0.208 | 8 | 0.592 | 0.001 |
| Sme2.5_02584.1_g00007.1 | geraniol dehydrogenase 1-like | 39665.22 | 0.315 | 6 | 0.643 | 0.002 |
| Sme2.5_01610.1_g00007.1 | glucose-6-phosphate 1-dehydrogenase cytoplasmic isoform-like | 69830.66 | 0.130 | 8 | 0.541 | 0.001 |
| Sme2.5_03917.1_g00002.1 | AGO2A2 | 114111.60 | 0.114 | 9 | 0.559 | 0.001 |
| Sme2.5_10440.1_g00003.1 | probable phosphoribosylformylglycinamidine synthase, chloroplastic/mitochondrial-like | 159319.60 | 0.167 | 18 | 0.494 | 0.001 |
| Sme2.5_01346.1_g00008.1 | uncharacterized protein At5g22580-like isoform 1 | 12104.20 | 0.434 | 4 | 0.361 | 0.001 |
| Sme2.5_00276.1_g00012.1 | transaldolase-like protein | 49225.14 | 0.359 | 12 | 0.549 | 0.001 |
| Sme2.5_00006.1_g00009.1 | proteinase inhibitor IIa | 16943.50 | 0.456 | 5 | 0.109 | 0.005 |
| Sme2.5_00039.1_g00019.1 | tubulin alpha chain-like | 48448.84 | 0.548 | 4 | 0.539 | 0.001 |
| Sme2.5_03603.1_g00006.1 | uncharacterized protein LOC101246887 | 61892.76 | 0.099 | 4 | 0.423 | 0.004 |
| Sme2.5_01158.1_g00001.1 | NADPH--cytochrome P450 reductase-like | 130829.80 | 0.084 | 6 | 0.558 | 0.002 |
| Sme2.5_00226.1_g00005.1 | uncharacterized protein LOC101247099 | 101282.80 | 0.043 | 4 | 0.514 | 0.049 |
| Sme2.5_02500.1_g00006.1 | 14-3-3 protein 7 | 26434.23 | 0.293 | 4 | 0.532 | 0.018 |
| Sme2.5_05446.1_g00002.1 | ubiquitin-activating enzyme E1 1-like isoform 1 | 115872.40 | 0.249 | 16 | 0.491 | 0.001 |
| Sme2.5_05299.1_g00002.1 | 60S ribosomal protein L22-2-like | 14097.39 | 0.336 | 2 | 0.626 | 0.004 |
| Sme2.5_02622.1_g00007.1 | heat shock protein 90-like | 88932.06 | 0.263 | 12 | 0.301 | 0.001 |
| Sme2.5_01754.1_g00007.1 | arginine--tRNA ligase, cytoplasmic-like | 42866.15 | 0.095 | 4 | 0.334 | 0.001 |
| Sme2.5_00018.1_g00014.1 | putative proline--tRNA ligase C19C7.06-like | 167566.30 | 0.095 | 9 | 0.557 | 0.001 |
| Sme2.5_23559.1_g00001.1 | unknown | 17411.30 | 0.371 | 3 | 0.627 | 0.008 |
| Sme2.5_24166.1_g00001.1 | FACT complex subunit SSRP1-like | 12218.90 | 0.429 | 4 | 0.418 | 0.001 |
| Sme2.5_00427.1_g00012.1 | probable 26S proteasome non-ATPase regulatory subunit 3-like | 57119.86 | 0.378 | 14 | 0.650 | 0.007 |
| Sme2.5_03336.1_g00005.1 | 29 kDa ribonucleoprotein B, chloroplastic | 32903.16 | 0.224 | 5 | 0.555 | 0.042 |
| Sme2.5_00411.1_g00008.1 | nucleosome assembly protein 1-like protein 2 | 45208.88 | 0.217 | 7 | 0.544 | 0.001 |
| Sme2.5_10384.1_g00003.1 | fructokinase 3 | 46074.55 | 0.340 | 8 | 0.602 | 0.001 |
| Sme2.5_12578.1_g00001.1 | 12S seed storage protein CRU2-like | 38872.12 | 0.301 | 8 | 0.409 | 0.001 |
| Sme2.5_02824.1_g00003.1 | phosphoenolpyruvate carboxylase-like | 109846.40 | 0.260 | 6 | 0.561 | 0.024 |
| Sme2.5_00297.1_g00010.1 | elongation factor G, chloroplastic-like | 98734.60 | 0.173 | 12 | 0.604 | 0.002 |
| Sme2.5_00024.1_g00030.1 | clathrin light chain 1-like isoform 1 | 36166.80 | 0.197 | 6 | 0.594 | 0.013 |
| Sme2.5_00536.1_g00016.1 | uncharacterized protein LOC101251994 | 124522.00 | 0.075 | 7 | 0.579 | 0.033 |
| Sme2.5_24391.1_g00001.1 | protochlorophyllide reductase A, chloroplastic | 43392.39 | 0.185 | 4 | 0.468 | 0.027 |
| Sme2.5_00776.1_g00002.1 | caffeoyl-CoA O-methyltransferase 6-like | 27700.24 | 0.147 | 2 | 0.479 | 0.007 |
| Sme2.5_01638.1_g00006.1 | PGR5-like protein 1A, chloroplastic-like | 38798.77 | 0.117 | 3 | 0.447 | 0.006 |
| Sme2.5_05323.1_g00005.1 | glutamate-1-semialdehyde 2,1-aminomutase, chloroplastic | 37172.05 | 0.473 | 9 | 0.584 | 0.003 |
| Sme2.5_03432.1_g00008.1 | 60S ribosomal protein L24-like | 19818.88 | 0.272 | 5 | 0.304 | 0.001 |
| Sme2.5_04836.1_g00003.1 | uncharacterized protein LOC101248476 | 20493.19 | 0.310 | 5 | 0.414 | 0.005 |
| Sme2.5_01862.1_g00005.1 | heat shock 70 kDa protein 15-like | 76238.50 | 0.365 | 4 | 0.659 | 0.038 |
| Sme2.5_00406.1_g00012.1 | 60S ribosomal protein L7A-like | 29400.55 | 0.252 | 7 | 0.414 | 0.007 |
| Sme2.5_04796.1_g00004.1 | uncharacterized protein LOC101256605 | 145971.30 | 0.307 | 35 | 0.612 | 0.001 |
| Sme2.5_00014.1_g00037.1 | Chain M, Localization Of The Large Subunit Ribosomal Proteins Into A 5.5 A Cryo-Em Map Of Triticum Aestivum Translating 80s Ribosome | 15158.07 | 0.514 | 6 | 0.657 | 0.002 |
| Sme2.5_01240.1_g00004.1 | sterol reductase | 66415.78 | 0.388 | 17 | 0.501 | 0.001 |
| Sme2.5_00006.1_g00031.1 | plastidic glucose transporter 4-like isoform 1 | 82346.68 | 0.047 | 3 | 0.654 | 0.010 |
| Sme2.5_04411.1_g00004.1 | DEAD-box ATP-dependent RNA helicase 37-like isoform 1 | 65204.40 | 0.085 | 4 | 0.359 | 0.001 |
| Sme2.5_00191.1_g00010.1 | uncharacterized protein LOC101247392 | 16998.04 | 0.562 | 6 | 0.639 | 0.002 |
| Sme2.5_02703.1_g00006.1 | zinc finger CCCH domain-containing protein 14-like | 32995.43 | 0.210 | 3 | 0.509 | 0.006 |
| Sme2.5_01462.1_g00018.1 | actin-7-like | 41967.98 | 0.610 | 5 | 0.588 | 0.001 |
| Sme2.5_08172.1_g00001.1 | peptidyl-prolyl cis-trans isomerase FKBP53-like | 55929.66 | 0.128 | 5 | 0.247 | 0.023 |
| Sme2.5_00085.1_g00024.1 | magnesium-protoporphyrin IX monomethyl ester [oxidative] cyclase, chloroplastic-like | 47744.57 | 0.106 | 3 | 0.272 | 0.001 |
| Sme2.5_04205.1_g00006.1 | protein disulfide-isomerase-like | 49309.43 | 0.261 | 7 | 0.650 | 0.024 |
| Sme2.5_02473.1_g00006.1 | annexin P38 | 44046.84 | 0.436 | 15 | 0.588 | 0.001 |
| Sme2.5_00024.1_g00013.1 | monothiol glutaredoxin-S17-like | 51555.06 | 0.153 | 5 | 0.617 | 0.004 |
| Sme2.5_00029.1_g00002.1 | nicotinate phosphoribosyltransferase-like | 66377.43 | 0.063 | 3 | 0.351 | 0.022 |
| Sme2.5_03689.1_g00007.1 | 30S ribosomal protein S1, chloroplastic-like | 45332.51 | 0.267 | 10 | 0.450 | 0.001 |
| Sme2.5_05287.1_g00005.1 | GDP-mannose 3',5'-epimerase | 42899.10 | 0.322 | 4 | 0.506 | 0.001 |
| Sme2.5_00950.1_g00009.1 | vacuolar protein sorting-associated protein 35B-like | 102083.40 | 0.028 | 3 | 0.651 | 0.029 |
| Sme2.5_00232.1_g00001.1 | Luminal-binding protein 5 | 73542.99 | 0.596 | 9 | 0.485 | 0.001 |
