# Supplementary material for: Comparative proteomic analysis of eggplant (Solanum melongena L.) heterostylous pistil development
Source: PLoS One. 2017 Jun 6;12(6):e0179018. doi: 10.1371/journal.pone.0179018 (PMC5460878; doi:10.1371/journal.pone.0179018)
Supplement: S6 Table — (DOCX) [file pone.0179018.s011.docx]

**Table S6. DEPs between S-morph and L-morph flowers enriched in each pathway during development.**

| **No.** | **Pathway** | **Proteins** |
| --- | --- | --- |
| 1 | Phenylpropanoid biosynthesis | Sme2.5_04891.1_g00002.1, Sme2.5_00368.1_g00010.1, Sme2.5_00468.1_g00005.1, Sme2.5_14644.1_g00002.1, Sme2.5_00745.1_g00004.1, Sme2.5_09669.1_g00005.1, Sme2.5_05614.1_g00005.1, Sme2.5_12240.1_g00001.1 |
| 2 | Cyanoamino acid metabolism | Sme2.5_00468.1_g00005.1, Sme2.5_14644.1_g00002.1, Sme2.5_09669.1_g00005.1, Sme2.5_05614.1_g00005.1, Sme2.5_12240.1_g00001.1 |
| 3 | Metabolic pathways (no map in kegg database) | Sme2.5_04401.1_g00002.1, Sme2.5_03497.1_g00004.1, Sme2.5_04891.1_g00002.1, Sme2.5_31478.1_g00001.1, Sme2.5_07446.1_g00003.1, Sme2.5_00368.1_g00010.1, Sme2.5_03231.1_g00008.1, Sme2.5_00468.1_g00005.1, Sme2.5_04260.1_g00006.1, Sme2.5_28714.1_g00002.1, Sme2.5_07601.1_g00002.1, Sme2.5_06878.1_g00001.1, Sme2.5_14644.1_g00002.1, Sme2.5_09858.1_g00002.1, Sme2.5_00346.1_g00019.1, Sme2.5_00345.1_g00027.1, Sme2.5_00745.1_g00004.1, Sme2.5_10015.1_g00002.1, Sme2.5_09669.1_g00005.1, Sme2.5_03454.1_g00001.1, Sme2.5_05614.1_g00005.1, Sme2.5_12240.1_g00001.1 |
| 4 | Starch and sucrose metabolism | Sme2.5_00468.1_g00005.1, Sme2.5_14644.1_g00002.1, Sme2.5_09669.1_g00005.1, Sme2.5_05614.1_g00005.1, Sme2.5_12240.1_g00001.1 |
| 5 | Glycine, serine and threonine metabolism | Sme2.5_04401.1_g00002.1, Sme2.5_00345.1_g00027.1, Sme2.5_03454.1_g00001.1 |
| 6 | Glyoxylate and dicarboxylate metabolism | Sme2.5_07446.1_g00003.1, Sme2.5_03231.1_g00008.1, Sme2.5_09858.1_g00002.1 |
| 7 | Nitrogen metabolism | Sme2.5_03906.1_g00010.1, Sme2.5_00512.1_g00007.1 |
| 8 | alpha-Linolenic acid metabolism | Sme2.5_07601.1_g00002.1, Sme2.5_00572.1_g00010.1 |
| 9 | Other glycan degradation (no map in kegg database) | Sme2.5_00468.1_g00005.1, Sme2.5_03184.1_g00003.1, Sme2.5_00275.1_g00003.1 |
| 10 | Biosynthesis of secondary metabolites (no map in kegg database) | Sme2.5_03497.1_g00004.1, Sme2.5_04891.1_g00002.1, Sme2.5_00368.1_g00010.1, Sme2.5_00468.1_g00005.1, Sme2.5_04260.1_g00006.1, Sme2.5_07601.1_g00002.1, Sme2.5_14644.1_g00002.1, Sme2.5_00346.1_g00019.1, Sme2.5_00345.1_g00027.1, Sme2.5_00745.1_g00004.1, Sme2.5_09669.1_g00005.1, Sme2.5_05614.1_g00005.1, Sme2.5_12240.1_g00001.1 |
| 11 | Carbon fixation in photosynthetic organisms | Sme2.5_07446.1_g00003.1, Sme2.5_03231.1_g00008.1, Sme2.5_09858.1_g00002.1 |
| 12 | Terpenoid backbone biosynthesis | Sme2.5_00468.1_g00005.1, Sme2.5_04260.1_g00006.1 |
| 13 | Cutin, suberine and wax biosynthesis | Sme2.5_03184.1_g00003.1, Sme2.5_06878.1_g00001.1 |
| 14 | Glycerolipid metabolism | Sme2.5_00468.1_g00005.1, Sme2.5_12240.1_g00001.1 |
| 15 | Amino sugar and nucleotide sugar metabolism | Sme2.5_31478.1_g00001.1, Sme2.5_28714.1_g00002.1, Sme2.5_10015.1_g00002.1 |
| 16 | Fatty acid degradation | Sme2.5_03497.1_g00004.1, Sme2.5_07601.1_g00002.1 |
| 17 | Circadian rhythm - plant | Sme2.5_00346.1_g00019.1 |
| 18 | Lysine degradation | Sme2.5_03454.1_g00001.1 |
| 19 | Base excision repair | Sme2.5_04309.1_g00005.1 |
| 20 | Peroxisome | Sme2.5_03184.1_g00003.1, Sme2.5_03454.1_g00001.1 |
| 21 | Ubiquinone and other terpenoid-quinone biosynthesis | Sme2.5_00368.1_g00010.1 |
| 22 | Phenylalanine metabolism | Sme2.5_00368.1_g00010.1 |
| 23 | Degradation of aromatic compounds (no map in kegg database) | Sme2.5_03497.1_g00004.1 |
| 24 | Flavonoid biosynthesis | Sme2.5_00346.1_g00019.1 |
| 25 | Carbon metabolism (no map in kegg database) | Sme2.5_03497.1_g00004.1, Sme2.5_07446.1_g00003.1, Sme2.5_03231.1_g00008.1, Sme2.5_09858.1_g00002.1 |
| 26 | Plant-pathogen interaction | Sme2.5_01085.1_g00002.1, Sme2.5_08282.1_g00001.1 |
| 27 | Alanine, aspartate and glutamate metabolism | Sme2.5_00345.1_g00027.1 |
| 28 | Porphyrin and chlorophyll metabolism | Sme2.5_00468.1_g00005.1 |
| 29 | Fatty acid metabolism (no map in kegg database) | Sme2.5_07601.1_g00002.1 |
| 30 | Tyrosine metabolism | Sme2.5_03497.1_g00004.1 |
| 31 | Glutathione metabolism | Sme2.5_03854.1_g00004.1 |
| 32 | Cysteine and methionine metabolism | Sme2.5_00345.1_g00027.1 |
| 33 | Endocytosis | Sme2.5_04696.1_g00006.1 |
| 34 | Protein processing in endoplasmic reticulum | Sme2.5_01810.1_g00004.1 |
| 35 | Glycolysis / Gluconeogenesis | Sme2.5_03497.1_g00004.1 |
