# Supplementary material for: Comparative proteomic analysis of eggplant (Solanum melongena L.) heterostylous pistil development
Source: PLoS One. 2017 Jun 6;12(6):e0179018. doi: 10.1371/journal.pone.0179018 (PMC5460878; doi:10.1371/journal.pone.0179018)
Supplement: S7 Table — (DOCX) [file pone.0179018.s012.docx]

**Table S7. DEPs between S-morph and L-morph flowers enriched in each pathway during maturity.**

| **No.** | **Pathway** | **Proteins** |
| --- | --- | --- |
| 1 | Ribosome | Sme2.5_01984.1_g00005.1, Sme2.5_00179.1_g00004.1, Sme2.5_00076.1_g00003.1, Sme2.5_01374.1_g00009.1, Sme2.5_06227.1_g00005.1, Sme2.5_00942.1_g00003.1, Sme2.5_00310.1_g00014.1, Sme2.5_06364.1_g00001.1, Sme2.5_00676.1_g00001.1, Sme2.5_01952.1_g00004.1, Sme2.5_00594.1_g00001.1, Sme2.5_00151.1_g00009.1, Sme2.5_00281.1_g00013.1, Sme2.5_00265.1_g00010.1, Sme2.5_00343.1_g00001.1, Sme2.5_30393.1_g00001.1, Sme2.5_02308.1_g00006.1, Sme2.5_01918.1_g00003.1, Sme2.5_03836.1_g00005.1, Sme2.5_02632.1_g00002.1, Sme2.5_00108.1_g00014.1, Sme2.5_01494.1_g00003.1, Sme2.5_00026.1_g00018.1, Sme2.5_09948.1_g00002.1, Sme2.5_06588.1_g00003.1, Sme2.5_00079.1_g00001.1, Sme2.5_00940.1_g00015.1, Sme2.5_02268.1_g00004.1, Sme2.5_15806.1_g00001.1, Sme2.5_00423.1_g00008.1, Sme2.5_00036.1_g00030.1, Sme2.5_00014.1_g00037.1, Sme2.5_00088.1_g00019.1 |
| 2 | Starch and sucrose metabolism | Sme2.5_09773.1_g00001.1, Sme2.5_02083.1_g00006.1, Sme2.5_00188.1_g00004.1, Sme2.5_12729.1_g00004.1, Sme2.5_00086.1_g00011.1, Sme2.5_00086.1_g00012.1, Sme2.5_10801.1_g00001.1, Sme2.5_00223.1_g00004.1, Sme2.5_01618.1_g00012.1, Sme2.5_01764.1_g00007.1, Sme2.5_03252.1_g00002.1, Sme2.5_14644.1_g00002.1, Sme2.5_04720.1_g00004.1, Sme2.5_05142.1_g00002.1, Sme2.5_05614.1_g00005.1, Sme2.5_00188.1_g00003.1, Sme2.5_07124.1_g00003.1, Sme2.5_01674.1_g00010.1, Sme2.5_12240.1_g00001.1 |
| 3 | Pentose and glucuronate interconversions | Sme2.5_09773.1_g00001.1, Sme2.5_02083.1_g00006.1, Sme2.5_00188.1_g00004.1, Sme2.5_10801.1_g00001.1, Sme2.5_00223.1_g00004.1, Sme2.5_01618.1_g00012.1, Sme2.5_03252.1_g00002.1, Sme2.5_00188.1_g00003.1 |
| 4 | Phenylpropanoid biosynthesis | Sme2.5_30554.1_g00001.1, Sme2.5_12729.1_g00004.1, Sme2.5_01764.1_g00007.1, Sme2.5_14644.1_g00002.1, Sme2.5_00001.1_g00048.1, Sme2.5_02369.1_g00001.1, Sme2.5_04720.1_g00004.1, Sme2.5_00776.1_g00002.1, Sme2.5_02584.1_g00008.1, Sme2.5_05614.1_g00005.1, Sme2.5_12240.1_g00001.1 |
| 5 | Cyanoamino acid metabolism | Sme2.5_12729.1_g00004.1, Sme2.5_01764.1_g00007.1, Sme2.5_14644.1_g00002.1, Sme2.5_04720.1_g00004.1, Sme2.5_05614.1_g00005.1, Sme2.5_12240.1_g00001.1 |
| 6 | Flavonoid biosynthesis | Sme2.5_00188.1_g00020.1, Sme2.5_00015.1_g00020.1, Sme2.5_00001.1_g00048.1, Sme2.5_00776.1_g00002.1, Sme2.5_01638.1_g00005.1 |
| 7 | Proteasome | Sme2.5_02098.1_g00007.1, Sme2.5_03722.1_g00006.1, Sme2.5_02533.1_g00006.1, Sme2.5_06455.1_g00005.1, Sme2.5_02824.1_g00005.1 |
| 8 | Sesquiterpenoid and triterpenoid biosynthesis | Sme2.5_21139.1_g00001.1 |
| 9 | Stilbenoid, diarylheptanoid and gingerol biosynthesis | Sme2.5_00001.1_g00048.1, Sme2.5_00776.1_g00002.1 |
| 10 | Isoquinoline alkaloid biosynthesis | Sme2.5_25992.1_g00001.1, Sme2.5_11776.1_g00001.1 |
| 11 | Metabolic pathways (no map in kegg database) | Sme2.5_09773.1_g00001.1, Sme2.5_02083.1_g00006.1, Sme2.5_00065.1_g00022.1, Sme2.5_00188.1_g00020.1, Sme2.5_00188.1_g00004.1, Sme2.5_00188.1_g00008.1, Sme2.5_03722.1_g00005.1, Sme2.5_02187.1_g00002.1, Sme2.5_01431.1_g00003.1, Sme2.5_07288.1_g00002.1, Sme2.5_25992.1_g00001.1, Sme2.5_30554.1_g00001.1, Sme2.5_12729.1_g00004.1, Sme2.5_00086.1_g00011.1, Sme2.5_00086.1_g00012.1, Sme2.5_10801.1_g00001.1, Sme2.5_00223.1_g00004.1, Sme2.5_02955.1_g00005.1, Sme2.5_01618.1_g00012.1, Sme2.5_04696.1_g00001.1, Sme2.5_00377.1_g00016.1, Sme2.5_01764.1_g00007.1, Sme2.5_03252.1_g00002.1, Sme2.5_14644.1_g00002.1, Sme2.5_00015.1_g00020.1, Sme2.5_00097.1_g00005.1, Sme2.5_00001.1_g00048.1, Sme2.5_07653.1_g00001.1, Sme2.5_02369.1_g00001.1, Sme2.5_01701.1_g00006.1, Sme2.5_13015.1_g00001.1, Sme2.5_01826.1_g00003.1, Sme2.5_00499.1_g00004.1, Sme2.5_00188.1_g00007.1, Sme2.5_04720.1_g00004.1, Sme2.5_00162.1_g00020.1, Sme2.5_00776.1_g00002.1, Sme2.5_03911.1_g00003.1, Sme2.5_02584.1_g00008.1, Sme2.5_00026.1_g00001.1, Sme2.5_05142.1_g00002.1, Sme2.5_11776.1_g00001.1, Sme2.5_05614.1_g00005.1, Sme2.5_00813.1_g00013.1, Sme2.5_00188.1_g00003.1, Sme2.5_01638.1_g00005.1, Sme2.5_07124.1_g00003.1, Sme2.5_01674.1_g00010.1, Sme2.5_05293.1_g00002.1, Sme2.5_12240.1_g00001.1, Sme2.5_01731.1_g00001.1, Sme2.5_05365.1_g00004.1, Sme2.5_00118.1_g00007.1 |
| 12 | Taurine and hypotaurine metabolism | Sme2.5_00499.1_g00004.1 |
| 13 | Terpenoid backbone biosynthesis | Sme2.5_21139.1_g00001.1, Sme2.5_01764.1_g00007.1, Sme2.5_04720.1_g00004.1 |
| 14 | Anthocyanin biosynthesis | Sme2.5_02148.1_g00009.1 |
| 15 | Glycerolipid metabolism | Sme2.5_01764.1_g00007.1, Sme2.5_04720.1_g00004.1, Sme2.5_12240.1_g00001.1 |
| 16 | Ubiquinone and other terpenoid-quinone biosynthesis | Sme2.5_00001.1_g00048.1, Sme2.5_00813.1_g00013.1 |
| 17 | Valine, leucine and isoleucine biosynthesis | Sme2.5_07653.1_g00001.1, Sme2.5_00162.1_g00020.1 |
| 18 | Protein export | Sme2.5_00377.1_g00016.1, Sme2.5_00232.1_g00001.1 |
| 19 | Phenylalanine metabolism | Sme2.5_00001.1_g00048.1, Sme2.5_00776.1_g00002.1 |
| 20 | Linoleic acid metabolism | Sme2.5_23355.1_g00001.1 |
| 21 | Carbon fixation in photosynthetic organisms | Sme2.5_03722.1_g00005.1, Sme2.5_02955.1_g00005.1, Sme2.5_04696.1_g00001.1, Sme2.5_00026.1_g00001.1 |
| 22 | alpha-Linolenic acid metabolism | Sme2.5_13015.1_g00001.1, Sme2.5_01731.1_g00001.1 |
| 23 | Riboflavin metabolism | Sme2.5_01701.1_g00006.1 |
| 24 | Selenocompound metabolism | Sme2.5_00097.1_g00005.1 |
| 25 | Monoterpenoid biosynthesis | Sme2.5_15018.1_g00001.1 |
| 26 | Zeatin biosynthesis | Sme2.5_02393.1_g00005.1 |
| 27 | Biosynthesis of secondary metabolites (no map in kegg database) | Sme2.5_00188.1_g00020.1, Sme2.5_01431.1_g00003.1, Sme2.5_25992.1_g00001.1, Sme2.5_30554.1_g00001.1, Sme2.5_12729.1_g00004.1, Sme2.5_02955.1_g00005.1, Sme2.5_00377.1_g00016.1, Sme2.5_01764.1_g00007.1, Sme2.5_14644.1_g00002.1, Sme2.5_00015.1_g00020.1, Sme2.5_00097.1_g00005.1, Sme2.5_00001.1_g00048.1, Sme2.5_07653.1_g00001.1, Sme2.5_02369.1_g00001.1, Sme2.5_01701.1_g00006.1, Sme2.5_13015.1_g00001.1, Sme2.5_00499.1_g00004.1, Sme2.5_04720.1_g00004.1, Sme2.5_00162.1_g00020.1, Sme2.5_00776.1_g00002.1, Sme2.5_02584.1_g00008.1, Sme2.5_00026.1_g00001.1, Sme2.5_11776.1_g00001.1, Sme2.5_15018.1_g00001.1, Sme2.5_05614.1_g00005.1, Sme2.5_00813.1_g00013.1, Sme2.5_01638.1_g00005.1, Sme2.5_12240.1_g00001.1, Sme2.5_01731.1_g00001.1 |
| 28 | Ascorbate and aldarate metabolism | Sme2.5_03911.1_g00003.1, Sme2.5_05293.1_g00002.1 |
| 29 | Cutin, suberine and wax biosynthesis | Sme2.5_05314.1_g00003.1, Sme2.5_15018.1_g00001.1 |
| 30 | RNA polymerase | Sme2.5_02187.1_g00002.1 |
| 31 | Porphyrin and chlorophyll metabolism | Sme2.5_01764.1_g00007.1, Sme2.5_04720.1_g00004.1 |
| 32 | beta-Alanine metabolism | Sme2.5_01431.1_g00003.1, Sme2.5_00499.1_g00004.1 |
| 33 | Oxidative phosphorylation | Sme2.5_00188.1_g00008.1, Sme2.5_00188.1_g00007.1, Sme2.5_05365.1_g00004.1 |
| 34 | Phagosome | Sme2.5_00864.1_g00008.1, Sme2.5_00125.1_g00003.1 |
| 35 | Other glycan degradation (no map in kegg database) | Sme2.5_02324.1_g00010.1, Sme2.5_01764.1_g00007.1, Sme2.5_04720.1_g00004.1 |
| 36 | N-Glycan biosynthesis | Sme2.5_00118.1_g00007.1 |
| 37 | [Base excision repair](file:///C:\Users\LAK\AppData\Local\Temp\Rar$EXa0.418\zd_13-VS-qc_13_fc1.5_map\map03410.html) | Sme2.5_04309.1_g00005.1 |
| 38 | [Amino sugar and nucleotide sugar metabolism](file:///C:\Users\LAK\AppData\Local\Temp\Rar$EXa0.418\zd_13-VS-qc_13_fc1.5_map\map00520.html) | Sme2.5_00086.1_g00011.1, Sme2.5_00086.1_g00012.1, Sme2.5_10801.1_g00001.1, Sme2.5_07124.1_g00003.1 |
| 39 | [Glycine, serine and threonine metabolism](file:///C:\Users\LAK\AppData\Local\Temp\Rar$EXa0.418\zd_13-VS-qc_13_fc1.5_map\map00260.html) | Sme2.5_07653.1_g00001.1, Sme2.5_00162.1_g00020.1 |
| 40 | [Pentose phosphate pathway](file:///C:\Users\LAK\AppData\Local\Temp\Rar$EXa0.418\zd_13-VS-qc_13_fc1.5_map\map00030.html) | Sme2.5_02955.1_g00005.1, Sme2.5_01196.1_g00005.1 |
| 41 | [Tyrosine metabolism](file:///C:\Users\LAK\AppData\Local\Temp\Rar$EXa0.418\zd_13-VS-qc_13_fc1.5_map\map00350.html) | Sme2.5_25992.1_g00001.1, Sme2.5_11776.1_g00001.1 |
| 42 | [Butanoate metabolism](file:///C:\Users\LAK\AppData\Local\Temp\Rar$EXa0.418\zd_13-VS-qc_13_fc1.5_map\map00650.html) | Sme2.5_00499.1_g00004.1 |
| 43 | [Galactose metabolism](file:///C:\Users\LAK\AppData\Local\Temp\Rar$EXa0.418\zd_13-VS-qc_13_fc1.5_map\map00052.html) | Sme2.5_10801.1_g00001.1, Sme2.5_01674.1_g00010.1 |
| 44 | [Pyruvate metabolism](file:///C:\Users\LAK\AppData\Local\Temp\Rar$EXa0.418\zd_13-VS-qc_13_fc1.5_map\map00620.html) | Sme2.5_03722.1_g00005.1, Sme2.5_04696.1_g00001.1, Sme2.5_00377.1_g00016.1 |
| 45 | [Propanoate metabolism](file:///C:\Users\LAK\AppData\Local\Temp\Rar$EXa0.418\zd_13-VS-qc_13_fc1.5_map\map00640.html) | Sme2.5_01431.1_g00003.1, Sme2.5_00377.1_g00016.1 |
| 46 | Degradation of aromatic compounds (no map in kegg database) | Sme2.5_00001.1_g00048.1 |
| 47 | [Pyrimidine metabolism](file:///C:\Users\LAK\AppData\Local\Temp\Rar$EXa0.418\zd_13-VS-qc_13_fc1.5_map\map00240.html) | Sme2.5_02187.1_g00002.1, Sme2.5_07288.1_g00002.1 |
| 48 | [Endocytosis](file:///C:\Users\LAK\AppData\Local\Temp\Rar$EXa0.418\zd_13-VS-qc_13_fc1.5_map\map04144.html) | Sme2.5_02324.1_g00010.1, Sme2.5_00701.1_g00012.1, Sme2.5_12877.1_g00001.1, Sme2.5_00377.1_g00016.1 |
| 49 | [Arginine and proline metabolism](file:///C:\Users\LAK\AppData\Local\Temp\Rar$EXa0.418\zd_13-VS-qc_13_fc1.5_map\map00330.html) | Sme2.5_01635.1_g00012.1 |
| 50 | [Cysteine and methionine metabolism](file:///C:\Users\LAK\AppData\Local\Temp\Rar$EXa0.418\zd_13-VS-qc_13_fc1.5_map\map00270.html) | Sme2.5_00065.1_g00022.1, Sme2.5_00097.1_g00005.1 |
| 51 | [Fatty acid biosynthesis](file:///C:\Users\LAK\AppData\Local\Temp\Rar$EXa0.418\zd_13-VS-qc_13_fc1.5_map\map00061.html) | Sme2.5_00377.1_g00016.1 |
| 52 | [Glycerophospholipid metabolism](file:///C:\Users\LAK\AppData\Local\Temp\Rar$EXa0.418\zd_13-VS-qc_13_fc1.5_map\map00564.html) | Sme2.5_01731.1_g00001.1 |
| 53 | [Protein processing in endoplasmic reticulum](file:///C:\Users\LAK\AppData\Local\Temp\Rar$EXa0.418\zd_13-VS-qc_13_fc1.5_map\map04141.html) | Sme2.5_00377.1_g00016.1, Sme2.5_00125.1_g00003.1, Sme2.5_00118.1_g00007.1, Sme2.5_00232.1_g00001.1 |
| 54 | Biosynthesis of amino acids (no map in kegg database) | Sme2.5_01431.1_g00003.1, Sme2.5_02955.1_g00005.1, Sme2.5_00097.1_g00005.1, Sme2.5_07653.1_g00001.1, Sme2.5_00162.1_g00020.1, Sme2.5_00026.1_g00001.1 |
| 55 | [Phenylalanine, tyrosine and tryptophan biosynthesis](file:///C:\Users\LAK\AppData\Local\Temp\Rar$EXa0.418\zd_13-VS-qc_13_fc1.5_map\map00400.html) | Sme2.5_01431.1_g00003.1 |
| 56 | [Plant hormone signal transduction](file:///C:\Users\LAK\AppData\Local\Temp\Rar$EXa0.418\zd_13-VS-qc_13_fc1.5_map\map04075.html) | Sme2.5_00225.1_g00038.1, Sme2.5_15018.1_g00001.1 |
| 57 | [Alanine, aspartate and glutamate metabolism](file:///C:\Users\LAK\AppData\Local\Temp\Rar$EXa0.418\zd_13-VS-qc_13_fc1.5_map\map00250.html) | Sme2.5_00499.1_g00004.1 |
| 58 | [Plant-pathogen interaction](file:///C:\Users\LAK\AppData\Local\Temp\Rar$EXa0.418\zd_13-VS-qc_13_fc1.5_map\map04626.html) | Sme2.5_00076.1_g00003.1, Sme2.5_08282.1_g00001.1, Sme2.5_00225.1_g00038.1 |
| 59 | [Photosynthesis](file:///C:\Users\LAK\AppData\Local\Temp\Rar$EXa0.418\zd_13-VS-qc_13_fc1.5_map\map00195.html) | Sme2.5_01826.1_g00003.1 |
| 60 | [Peroxisome](file:///C:\Users\LAK\AppData\Local\Temp\Rar$EXa0.418\zd_13-VS-qc_13_fc1.5_map\map04146.html) | Sme2.5_05314.1_g00003.1, Sme2.5_15018.1_g00001.1 |
| 61 | [ABC transporters](file:///C:\Users\LAK\AppData\Local\Temp\Rar$EXa0.418\zd_13-VS-qc_13_fc1.5_map\map02010.html) | Sme2.5_00813.1_g00013.1 |
| 62 | Fatty acid metabolism (no map in kegg database) | Sme2.5_00377.1_g00016.1 |
| 63 | [Fructose and mannose metabolism](file:///C:\Users\LAK\AppData\Local\Temp\Rar$EXa0.418\zd_13-VS-qc_13_fc1.5_map\map00051.html) | Sme2.5_02955.1_g00005.1 |
| 64 | [Glutathione metabolism](file:///C:\Users\LAK\AppData\Local\Temp\Rar$EXa0.418\zd_13-VS-qc_13_fc1.5_map\map00480.html) | Sme2.5_00341.1_g00020.1 |
| 65 | Carbon metabolism (no map in kegg database) | Sme2.5_03722.1_g00005.1, Sme2.5_01431.1_g00003.1, Sme2.5_02955.1_g00005.1, Sme2.5_04696.1_g00001.1, Sme2.5_07653.1_g00001.1, Sme2.5_00162.1_g00020.1, Sme2.5_00026.1_g00001.1 |
| 66 | [Spliceosome](file:///C:\Users\LAK\AppData\Local\Temp\Rar$EXa0.418\zd_13-VS-qc_13_fc1.5_map\map03040.html) | Sme2.5_01984.1_g00017.1, Sme2.5_00396.1_g00018.1, Sme2.5_05314.1_g00003.1, Sme2.5_08226.1_g00002.1 |
| 67 | [Valine, leucine and isoleucine degradation](file:///C:\Users\LAK\AppData\Local\Temp\Rar$EXa0.418\zd_13-VS-qc_13_fc1.5_map\map00280.html) | Sme2.5_01431.1_g00003.1 |
| 68 | [Glycolysis / Gluconeogenesis](file:///C:\Users\LAK\AppData\Local\Temp\Rar$EXa0.418\zd_13-VS-qc_13_fc1.5_map\map00010.html) | Sme2.5_02955.1_g00005.1, Sme2.5_00026.1_g00001.1 |
| 69 | [Purine metabolism](file:///C:\Users\LAK\AppData\Local\Temp\Rar$EXa0.418\zd_13-VS-qc_13_fc1.5_map\map00230.html) | Sme2.5_02187.1_g00002.1 |
